# Surgical care in district hospitals in sub-Saharan Africa: a scoping review

**DOI:** 10.1136/bmjopen-2020-042862

**Published:** 2021-03-25

**Authors:** Zineb Bentounsi, Sharaf Sheik-Ali, Grace Drury, Chris Lavy

**Affiliations:** 1 Nuffield Department of Orthopaedics, Rheumatology and Musculoskeletal Sciences, University of Oxford, Oxford, UK; 2 Oxford University Hospitals NHS Foundation Trust, Oxford, UK

**Keywords:** surgery, health services administration & management, anaesthetics

## Abstract

**Objective:**

To provide a general overview of the reported current surgical capacity and delivery in order to advance current knowledge and suggest targets for further development and research within the region of sub-Saharan Africa.

**Design:**

Scoping review.

**Setting:**

District hospitals in sub-Saharan Africa.

**Data sources:**

PubMed and Ovid EMBASE from January 2000 to December 2019.

**Study selection:**

Studies were included if they contained information about types of surgical procedures performed, number of operations per year, types of anaesthesia delivered, cadres of surgical/anaesthesia providers and/or patients’ outcomes.

**Results:**

The 52 articles included in analysis provided information about 16 countries. District hospitals were a group of diverse institutions ranging from 21 to 371 beds. The three most frequently reported procedures were caesarean section, laparotomy and hernia repair, but a wide range of orthopaedics, plastic surgery and neurosurgery procedures were also mentioned. The number of operations performed per year per district hospital ranged from 239 to 5233. The most mentioned anaesthesia providers were non-physician clinicians trained in anaesthesia. They deliver mainly general and spinal anaesthesia. Depending on countries, articles referred to different surgical care providers: specialist surgeons, medical officers and non-physician clinicians. 15 articles reported perioperative complications among which surgical site infection was the most frequent. Fifteen articles reported perioperative deaths of which the leading causes were sepsis, haemorrhage and anaesthesia complications.

**Conclusion:**

District hospitals play a significant role in sub-Saharan Africa, providing both emergency and elective surgeries. Most procedures are done under general or spinal anaesthesia, often administered by non-physician clinicians. Depending on countries, surgical care may be provided by medical officers, specialist surgeons and/or non-physician clinicians. Research on safety, quality and volume of surgical and anaesthesia care in this setting is scarce, and more attention to these questions is required.

**Strengths and limitations of this study**

- This is the first review (of which we are aware) to systematically assess the literature for the scope, volume and quality of surgery in district hospitals in sub-Saharan Africa.
- Article selection and data extraction were conducted independently by two authors.
- Our search was conducted in English, French and Portuguese, and work in other languages may have been omitted.
- This study is prone to publication bias as it includes only data from published literature, and findings that were never published will have been omitted.
- There was a lot of heterogeneity in how articles reported surgical volume and quality.

## Introduction

The burden of surgical disease in sub-Saharan Africa is estimated at 38 disability adjusted life years per 1000 population. District hospitals bear a large burden of this surgical care delivery.^1^ According to The WHO, district hospitals are defined as the first level of healthcare facilities that provide in-patient surgery and anaesthesia.^2^ These hospitals mainly serve rural populations and face challenges in terms of workforce, infrastructure and other essential resources. In 2010, it was estimated that there was only 1 surgeon for 25 million people in rural sub-Saharan Africa^3^ and although training programmes in the region are increasing the number of surgeons, the main burden in several countries remains on non-physician clinicians to manage surgical cases.^4^

District hospitals offer a unique opportunity to improve access to surgical care in sub-Saharan Africa as they are the first point of access to surgical care for patients and relieve referral hospitals from undue burden, allowing them to focus on complicated cases. Delivering timely surgical care in district hospitals has been shown to be highly cost effective,^5^ which should provide encouragement for governments and global health programmes to invest in them. The Lancet Commission on Global Surgery,^6^ which defines scalable solutions for the provision of quality surgical and anaesthesia care for all, reinforces this emphasis on district hospitals and echoes other authors’ calls to consider district hospitals as a central component in strengthening surgical care in sub-Saharan Africa.^7 8^

We considered a systematic review of district hospital surgery; however, there were a limited number of existing studies in this field and a wide range of methodology used. Therefore, we chose to change our review methodology and undertake a scoping review of surgical care delivery in district hospitals in sub-Saharan Africa. Our aim is to provide a general overview of the reported current surgical capacity and delivery in order to advance current knowledge and suggest targets for further development and research within the region of sub-Saharan Africa.

## Methods

This scoping review follows the five-step methodology designed by Arksey and O’Malley^9^ that offers a rigorous framework for reporting findings. We have also considered the recommendations of Levac *et al*^10^ that supplement this framework.

### Identifying the research question

To guide our search of the literature, we defined the following questions:

#### Primary outcome

In district hospitals in sub-Saharan Africa, what types of surgical procedures are provided?

#### Secondary outcomes

In district hospitals in sub-Saharan Africa:

- How many surgical procedures are performed per year?
- Are there any measures of quality, safety or outcomes of surgery?
- Who provides anaesthesia and/or surgery?
- What type of anaesthesia is delivered?

### Identifying relevant studies

We searched PubMed and Ovid EMBASE on 18 December 2019 to retrieve literature about surgery in district hospitals in sub-Saharan Africa. The search strategy combined the exploded thesaurus terms ‘surgery’, ‘surgical procedures, operative’ and ‘specialties, surgical’ with the exploded thesaurus terms ‘hospitals, district’ and ‘Africa south of the Sahara’. We searched references in English, French and Portuguese. We limited the search to references published after 1 January 2000 in order to provide a recent overview and, therefore, studies published before 2000 were not relevant for this purpose. We also searched Google Scholar and the Cochrane Library with the same search terms, but they did not contribute any new relevant references.

### Study selection

The most important inclusion criterion was the setting: a district-level hospital in sub-Saharan Africa. We included articles that provided information about either type of surgical procedures performed, number of operations per year, types of anaesthesia delivered, cadres of surgical/anaesthesia providers and/or patient outcomes. We excluded articles if the setting was unclear or was not a district hospital and if they were strictly about costs or burden of disease. We excluded articles if the full text could not be retrieved. We included all surgical procedures except those typically done in outpatient departments, such as dental and ophthalmic procedures.

ZB and GD independently screened the abstracts using the web application Rayyan,^11^ resolving any conflicts by dialogue between ZB, GD and CL. The process of selection followed the Preferred Reporting of Items for Systematic Reviews and Meta-Analyses (PRISMA) Statement.

### Charting the data

Z.B. developed the data charting form after consultation with all other authors. The form was piloted and then updated iteratively during the extraction process (see online supplemental file 1). The form contained 20 fields of data entry covering information about articles (eg, name of first author, year of publication, research method) and information about district hospitals, surgical and anaesthesia providers, surgical procedures as well as other relevant information. ZB and SS-A extracted the data independently.

10.1136/bmjopen-2020-042862.supp1 Supplementary data

### Collating, summarising and reporting the results

SS-A produced descriptive numerical summaries of surgical volume, types of surgical procedures and types of anaesthesia using Excel. ZB performed a thematic analysis in NVivo^12^ with surgical providers, quality of care and surgical outcomes as themes. We reported results to answer the primary and secondary outcomes of the review. All authors discussed and reported implications for research and policy.

## Results

In this section, we first present characteristics of the included articles and an analysis of the district hospitals. Thereafter, we present our findings on the scope and volume of surgery, types of anaesthesia, surgical and anaesthesia providers and finally safety and quality of care.

### Study selection

**Figure 1:**
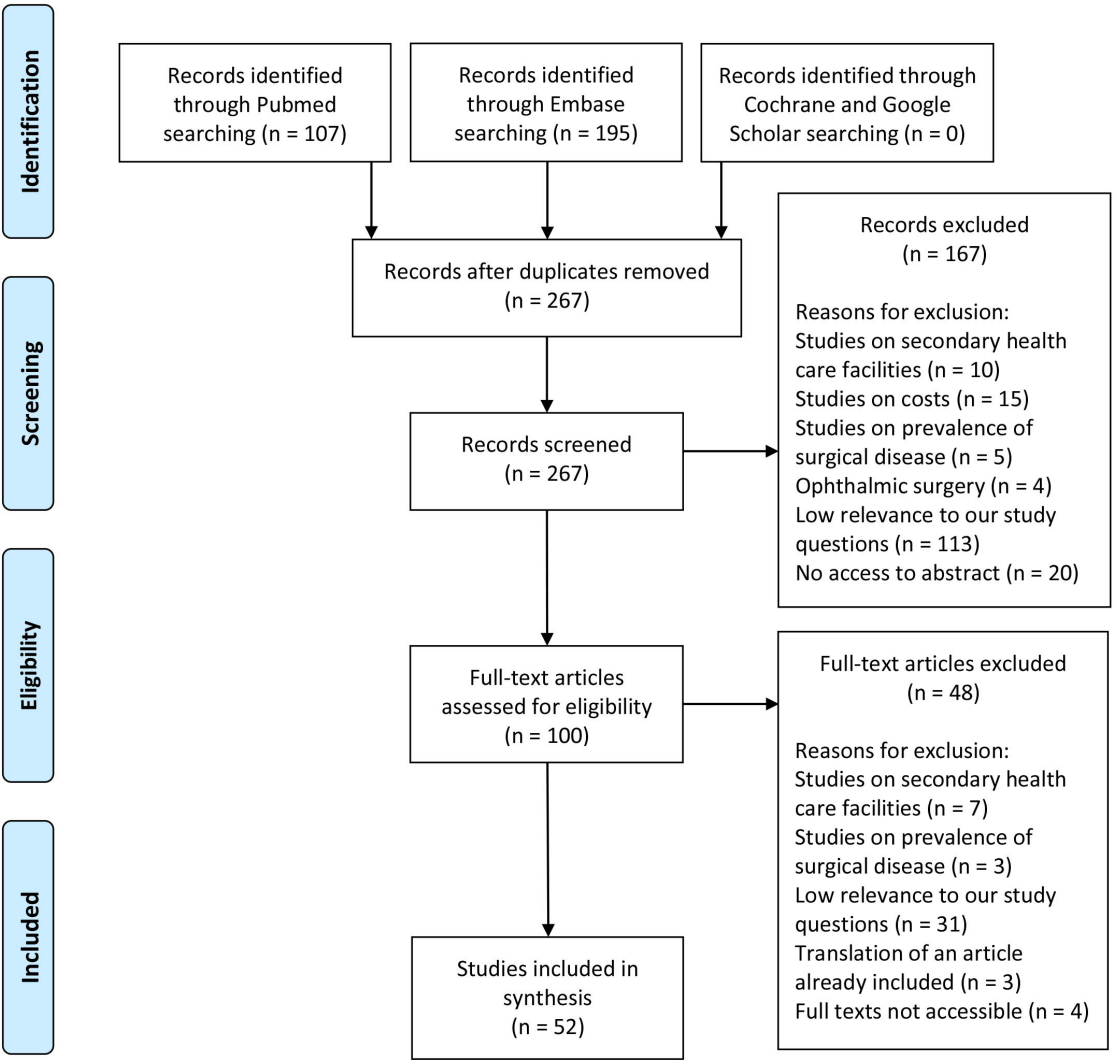
PRISMA diagram illustrating the selection process. PRISMA, Preferred Reporting of Items for Systematic Reviews and Meta-Analyses.

We identified 302 articles and after removing duplicates and 267 articles remained. We screened the titles and abstracts, and, using our criteria noted above, excluded 167 articles. Then, we assessed full texts of the remaining 100 articles and excluded 48. Therefore, we included 52 articles for analysis. A PRISMA diagram is presented in figure 1.

### Study characteristics

Thirty three per cent of articles (17 articles) were cross-sectional surveys, 17% were prospective studies (nine articles) and 11.5% (six articles) were based on retrospective review of hospital records (table 1). Ninety-three per cent of articles (48 articles) described the surgical care provided by staff based on the hospital, and the rest (four articles) reported external support from nongovernmental organisations (NGO) or visiting surgical teams^13–17^

**Table 1:** Summary of studies selected

First author	Year of publication	Study setting	Study design	Objective
Abdullah *et al*^30^	2011	Ghana	Mixed-methods	Assess surgical and obstetrical capacity
Cheelo *et al*^59^	2018	Zambia	Cross-sectional survey	Assess surgical capacity
Choo *et al*^61^	2013	Ghana	Cross- sectional survey	Appraise quality improvement activities
Choo *et al*^24^	2011	Ghana	Qualitative methods	Explore the training of medical officers providing surgery
Compaoré *et al*^31^	2014	Burkina Faso	Cross-sectional survey	Assess the readiness to provide caesarean sections
Damien *et al*^32^	2011	Ghana	Review of hospital records	Evaluate surgical volume
De Brouwere *et al*^56^	2009	Senegal	Mixed-methods	Evaluate the task shifting policy
Dossche *et al*^14^	2014	Burkina Faso	Case series	Analyse technical problems and post-operative complications
Dresser *et al*^58^	2017	Uganda	Review of hospital records	Evaluate the management of surgical patients by non-physician emergency clinicians
Fehr *et al*^25^	2006	Tanzania	Prospective cohort study	Identify risk factors for surgical site infection
Fehr *et al*^65^	2006	Tanzania	Prospective cohort study	Evaluate peri-operative antimicrobial prophylaxis
Fenton *et al*^39^	2003	Malawi	Prospective cohort study	Identify risk factors associated with maternal death after caesarean section
Gajewski *et al*^33^	2017	Malawi	Baseline and follow-up patients’ surveys	Compare surgical outcomes between central and district hospitals
Gajewski *et al*^18^	2019	Malawi, Tanzania, Zambia	Mixed-methods	Investigate anaesthesia capacity
Gajewski *et al*^34^	2018	Malawi	Qualitative methods	Explore providers’ perspectives on obstacles to surgery
Gajewski *et al*^35^	2019	Malawi	Randomised control trial	Evaluate surgical training programme for clinical officers
Galukande *et al*^19^	2010	Uganda, Tanzania, Mozambique	Review of hospital records	Assess the scope of surgery provided
Grimes *et al*^21^	2012	Sub-Saharan Africa	Systematic review	Identify the met and unmet needs of surgical disease
Gyedu *et al*^36^	2018	Ghana	Cross-sectional survey	Evaluate the operation rate in Ghana
Hall *et al*^37^	2015	Ghana	Review of hospital records	Evaluate the impact of a newly placed obstetrician on health outcomes
Harfouche *et al*^38^	2015	Malawi	Prospective cohort study	Assess the quality of emergency caesarean sections
Henry *et al*^22^	2015	Sub-Saharan Africa	Consensus meeting +interviews	Identify a package of essential surgical operations
Henry *et al*^55^	2015	Malawi	Cross-sectional survey	Assess surgical capacity
Kenfack *et al*^66^	2012	Cameroon	Case series	Describe the management of ectopic pregnancy
Koigi-Kamau *et al*^62^	2005	Kenya	Prospective cohort study	Describe the incidence of post-caesarean wound infection
Lavy *et al*^67^	2007	Malawi	Review of hospital records	Estimate surgical volume
LeBrun *et al*^20^	2014	Ethiopia, Liberia, Rwanda, Uganda	Cross- sectional survey	Assess surgical capacity
Lofgren *et al*^40^	2015	Uganda	Cross-sectional survey	Investigate indications, interventions and peri-operative mortality
Lonnée Lonn^66^	2018	Zimbabwe	Cross-sectional survey	Describe anaesthesia care for caesarean sections
Luo *et al*^45^	2018	Ghana	Qualitative methods	Explore the impact of a newly placed obstetrician in a district hospital
McCord^46^	2012	Ghana	Commentary	Describe surgical care in district hospitals in Ghana
McCord *et al*^57^	2009	Tanzania	Review of hospital records	Evaluate the quality of emergency obstetric surgery done by assistant medical officers
Mehtsun *et al*^47^	2012	Ghana	Observational study	Assess surgical workload of medical officers
Mpirimbanyi *et al*^17^	2017	Rwanda	Cross-sectional survey	Describe the spectrum, management and patient outcomes of emergency surgery
Muhirwa *et al*^15^	2016	Rwanda	Cohort study	Describe the presentation, management and outcomes of surgical patients
Ngaroua *et al*^26^	2017	Cameroon	Case series	Describe the management of urethral stenosis
Nkurunziza *et al*^16^	2019	Rwanda	Cross-sectional survey	Estimate the prevalence of surgical site infection after caesarean section
Nordberg *et al*^49^	2002	Kenya	Cross-sectional survey	Estimate the surgical volume
Notrica *et al*^50^	2011	Rwanda	Cross-sectional survey	Assess surgical and anaesthesia infrastructure
Ottaway *et al*^41^	2018	Namibia	Prospective Observational Study	Determine the prevalence of anaesthetic adverse events
Ouédraogo *et al*^42^	2015	Burkina Faso	Prospective Observational Study	Describe task shifting in obstetric care
Ouédraogo *et al*^43^	2015	Burkina Faso	Case series	Describe prognosis of patients undergoing caesarean sections
Petroze *et al*^51^	2012	Rwanda	Cross-sectional survey	Evaluate the ratio of caesarean sections to total procedures as a marker of trauma capacity
Petroze *et al*^52^	2012	Rwanda	Mixed-methods	Assess emergency and essential surgical capacity
Ramos *et al*^13^	2013	Ethiopia	Case series	Describe thyroid surgery cases
Rutgers and VanEygen^53^	2008	Zimbabwe	Cross-sectional survey	Describe the mortality associated with caesarean sections
Sani *et al*^54^	2009	Niger	Cross-sectional survey	Assess surgical care provided by general practitioners
Sherman *et al*^23^	2011	Liberia	Cross-sectional survey	Assess emergency and essential surgical care
Smiley *et al*^48^	2019	Ghana	Surveys+process mapping	Evaluate patients and staff perception of quality of care + analyse peri-operative process
Stewart *et al*^63^	2015	Ghana	Cross-sectional survey	Assess trauma capacity
Van Amelsfoort *et al*^29^	2010	Malawi	Mixed-methods	Describe the training and experiences of clinical officers
Van den Akker *et al*^64^	2009	Malawi	Mixed-methods	Evaluate the impact of audits in reducing the incidence of uterine ruptures

Of the 52 articles included for analysis, covering 16 countries, 3 articles presented data from multiple countries^18–20^ and 2 gave a regional perspective.^21 22^ Ghana, Malawi and Rwanda were the three countries most represented in the articles selected, partly because of research projects in collaboration with researchers from high-income countries (table 2).

**Table 2:** Distribution of articles per country

Country	Number of articles
Burkina Faso	3
Cameroon	2
Ethiopia	2
Ghana	11
Kenya	2
Liberia	2
Malawi	10
Mozambique	1
Namibia	1
Niger	1
Rwanda	7
Senegal	1
Sub Saharan Africa (regional perspective)	2
Tanzania	5
Uganda	4
Zambia	2
Zimbabwe	2

### Characteristics of district hospitals

District hospitals described in this review included a variety of institutions (table 3). In our review, the two smallest district hospitals had 21 beds, reported in Liberia and Ghana by Sherman *et al*^23^ and Choo *et al,*^24^ respectively. The biggest district hospital had 371 beds, reported by Fehr *et al* in Tanzania.^25^ District hospitals served rural or urban populations, with some serving both groups (for example, if they were situated on the edge of towns). Although most were owned by the government, mission-linked district hospitals also featured in several papers. On some occasions, hospitals received support from visiting surgeons or NGOs, for example, in Rwanda, government-owned district hospitals received support from partners in health.^15^

**Table 3:** Distribution of articles per size of district hospitals

Size of district hospitals (in number of beds)	Number of articles
<20	0
20–50	2
51–100	8
101–300	9
>300	2
Not specified	33

### Scope of surgical operations

The majority of articles mentioned obstetrics/gynaecology and general surgery operations (table 4). Indeed, the three most frequently reported procedures were caesarean section, laparotomy and hernia repair. However, a wide range of other surgical procedures were mentioned across the literature (online supplemental file 2). These were mainly in orthopaedics, plastic surgery or neurosurgery and may have involved visiting specialists (table 4). District hospitals reported in the literature handled both emergency procedures (such as caesarean sections, appendicectomies and treatment of burns) and elective procedures (such as hernia repairs) and some even more complex procedures such as direct vision internal urethrotomy^26^ and hip replacement.^14^ Despite the high prevalence of traumatic brain injury in sub-Saharan Africa^27^ and the fact that cranial burr holes are included in the WHO book ‘Surgical Care at the District Hospital’,^28^ only three articles mentioned burr holes.

10.1136/bmjopen-2020-042862.supp2 Supplementary data

**Table 4:** Distribution of articles per surgical operations

Surgical procedures	Number of articles
General surgery	
Hernia repair	26
Laparotomy	21
Appendicectomy	10
Abscess	7
Hydrocele repair	7
Thyroid surgery	5
Colectomy	4
Chest tube insertion	4
Prostatectomy	4
Cricothyroidotomy	3
Colostomy	2
Lipoma	2
Tracheostomy	2
Fibroma	1
Urethrotomy	1
Vascular repair	1
Bladder stone surgery	1
Obstetrics and gynaecology	
Caesarean section	30
Salpingectomy (ectopic pregnancy)	10
Hysterectomy	9
Evacuation of retained products of conception	7
Tubal ligation	5
Fistula repair	3
Cervical tear repair	2
Breast surgery	1
Orthopaedics	
Amputation	10
Open fracture repair	8
Closed fracture repair	5
Arthrotomy	3
Osteotomy	2
Total hip replacement	1
Bipolar hemiarthroplasty	1
Neurosurgery	
Major neurosurgery	1
Burr holes	3
Plastics	
Skin grafting	7
Debridement	5
Burn	4
Cleft lip/palate surgery	3
Contracture release	2
Escharotomy	1

### Surgical volume

Only 13 articles provided enough data to be able to estimate the number of operations per year per district hospital performed. In some cases, there was not enough information to discriminate between major and minor operations, therefore, the choice was made to present all operations combined. Using a simple formula, the number of operations, period of years and number of district hospitals, an average of the number of operations per district hospital per year was calculated (table 5). Galukande *et al*^19^ and LeBrun *et al*^20^ contributed several reports on different district hospitals while van Amelsfoort *et al*^29^ contributed two reports on the same district hospital during two different time periods.

**Table 5:** Surgical volume (all reported cases) in number of operations per year per district hospital

First author	Study setting	Total number of operations	Years	Number of district hospitals	Estimated number of operations per district hospital per year (to the nearest unit)
Abdullah*^30^	Ghana	–	1	10	774
Damien^32^	Ghana	1391	5	1	278
Galukande	Tanzania	980	1	1	980
Galukande	Tanzania	2045	1	1	2045
Galukande	Mozambique	601	1	1	601
Galukande	Mozambique	256	1	1	256
Galukande	Uganda	1484	1	1	1484
Galukande	Uganda	248	1	1	248
Galukande	Uganda	239	12	1	239
Galukande	Uganda	1835	1	1	1835
Gyedu	Ghana	143 750	1	48	2995
Lofgren †	Uganda	–	–	2	5018
Lavy^67^	Malawi	25 053	1	21	1193
LeBrun‡	Uganda	–	1	1	1296
LeBrun‡	Rwanda	–	1	1	2052
LeBrun‡	Liberia	–	1	1	696
LeBrun‡	Ethiopia	–	1	1	538
Nordberg*	Kenya	–	1	5	5233
Notrica‡	Rwanda	–	–	–	2052
Ottaway	Namibia	737	0.58	4	316
Petroze	Rwanda	71 432	1	41	1742
Sani	Niger	544	1	3	181
Van Amelsfoort	Malawi	19 644	1	17	1156
Van Amelsfoort	Malawi	18 524	1	17	1090

*The article reported numbers for both major and minor operations and we calculated the total number of procedures.

†This article reported that the number of major operations was estimated over a 4 month period while the number of minor operations was estimated over a 3 month period.

‡Reported in the article as average number per year per district hospital.

The surgical volume of district hospitals varied from circa 200 to circa 5000 operations per year, with the majority of hospitals performing between 1001 and 5000 operations per year (tables 5 and 6). Clear limitations to the use of these data are the sample size and selection bias. Of the 13 studies, only 6 analysed more than five district hospitals in a given area. These data, therefore, cannot solely be used as a general indicator for the productivity of district hospitals in the countries represented.

**Table 6:** Number of publications reporting the number of operations per year per district hospital

Number of operations on average per district hospital over 1 year	Number of publications
<100	0
100–500	6
501–1000	5
1001–5000	11
>5000	2

### Types of anaesthesia

Out of 52 articles, 16 contained information about the type of anaesthesia used in the district hospitals studied. General anaesthesia was mentioned by 15 articles^13 15 17–19 21 30–38^ and spinal anaesthesia by 10 articles.^14 17 23 24 39–44^ Local anaesthesia and regional blocks were mentioned by four articles.^15 17 23 40^

### Surgical and anaesthesia providers

In each country, different cadres of healthcare professionals are responsible for providing surgical care in district hospitals (table 7). In Ethiopia, Ramos *et al* reported the presence of a specialist surgeon.^13^ In Ghana,^24 30 36 37 45–48^ Kenya,^49^ Rwanda^15–17 50–52^ and Zimbabwe^53^ medical officers worked alongside specialist surgeons or obstetrician/gynaecologists to provide surgical care. In Namibia^41^ and Niger,^54^ the presence of medical officers as sole surgical providers was reported. Non-physician clinicians provided surgical care in Burkina Faso,^14 31 42^ Liberia,^23^ Malawi,^33–35 39 55^ Senegal,^56^ Tanzania,^18 57^ Uganda^40 58^ and Zambia.^59^

**Table 7:** Number of publications reporting surgical providers

Surgical providers	Number of publications
Specialist surgeon/obstetrician	5
Specialist surgeon/obstetrician & medical officers	9
Medical officers	8
Medical officers and non-physician clinicians	4
Specialist surgeon/obstetrician and non-physician clinicians	1
Non-physician clinicians	5
Specialists and medical officers and non-physician clinicians	8

Similarly, different cadres performed anaesthesia in different countries.

Articles reported the presence of a physician anaesthetist in district hospitals in Burkina Faso^14^ and Ghana.^48^ Medical officers providing anaesthesia on their own were reported in Namibia.^41^ Otherwise, the most mentioned anaesthesia providers are non-physician clinicians trained in anaesthesia (table 8). Their presence is reported in Burkina Faso,^31^ Ethiopia,^13^ Ghana,^24^ Liberia,^23^ Malawi,^20 28^ Rwanda,^51 56^ Senegal,^56^ Tanzania,^18^ Uganda,^40^ Zambia^59^ and Zimbabwe.^57 60^ There are also untrained non-physician clinicians providing anaesthesia in Liberia,^23^ Malawi,^20 28^ Rwanda,^51 56^ Zimbabwe^57 60^ alongside trained personnel.

**Table 8:** Number of publications reporting anaesthesia providers

Anaesthesia providers	Number of publications
Physician anaesthetists	2
Medical officers	1
Medical officer and non-physician clinicians	1
Non-physician clinicians (trained)	10
Non-physician clinicians (trained and untrained)	6

### Quality of care and surgical outcomes

#### Quality of care

Of all articles in our review, only nine articles evaluated the quality of care as part of their study. Choo *et al* surveyed 10 district hospitals in Ghana about quality improvement activities.^61^ None of the hospitals surveyed reported an organised quality improvement programme (although this does not mean it was absent), and the only quality improvement activity related to surgery was found in one hospital and related to sterilisation of instruments.^61^ Other authors echoed these findings by reporting a lack of clinical guidelines for surgery, anaesthesia and labour management^23 31 62^ and a lack of staff trained in using them when they were available.^31^

To evaluate the quality of caesarean sections, Harfouche *et al* measured the ‘decision to incision time’ in a district hospital in Malawi and found that it reached ‘1.69 hours per caesarean section, far exceeding the recommended 30 min’.^38^ They explained their findings only by a ‘lack of operating theatre space and high patient volume’ but also by an inefficient system to transfer patients to the operating theatre.^38^

Another major challenge to quality care in district hospitals in sub-Saharan Africa is the shortage of trained surgical and anaesthesia staff. Stewart *et al*^63^ explained that shortage in staff resulted in unavailability of service during nights and weekends. Choo *et al* found that medical officers in Ghana had limited exposure to surgery during their training.^24^ Harfouche *et al* reported that while clinical officers performed most caesarean sections at district level in Malawi, they were not sufficiently trained in performing emergency hysterectomies.^38^ Positive outcomes were observed in cases where trained staff were present: Hall *et al* reported an ‘association between the presence of Obstetricians and Gynaecologists in district hospitals in Ghana and an increase in evidence-based maternal care practices as well as moderate improvement in maternal mortality and stillbirth rates’.^37^

Some authors suggested interventions to improve the quality of care. Van den Akker *et al* suggested audit as a ‘simple intervention requiring little technology’ that is able to improve quality of care.^64^ They reported a decrease in 68% of uterine ruptures in a district hospital in Malawi after implementing recommendations inferred from an audit. Recommendations included training of staff, better documentation and involvement of hospital management.^64^

Other authors insisted on the need for monitoring safety and quality of care in district hospitals as a first step toward improving quality.^40 61^

However, Smiley *et al* reported care of exceptional quality in a ‘63 bed district hospital’ named the ‘Volta River Authority Hospital’ in Ghana.^48^ In their study, they surveyed staff and patients about their perception of quality of care and conducted process mapping of surgical and obstetric care. They found that ‘over 80% of employees across a variety of perioperative roles held a positive view of teamwork and safety climate within the institution’ and ‘surgical patients who were surveyed gave similarly positive indications of overall satisfaction’.^48^ The perception of staff and patients was complemented by the observations of researchers during the process mapping. The WHO safety checklist was used in all operations witnessed and the authors reported the existence of a hospital wide morbidity and mortality conference as well as a safety training for new staff.^48^

#### Surgical outcomes

The most commonly used indicators of outcome were perioperative complications and perioperative mortality. Fifteen articles reported information about perioperative complications,^14–17 25 33 35 38 39 41 43 62 64–66^ the most reported being surgical site infection. Fifteen articles included information about perioperative mortality^10–12 15 16 25 26 30–32 35 37 49 61 62^; the most reported causes of death were sepsis, haemorrhage and anaesthesia complications.

In surgical obstetric care, the most reported complications were ruptured uterus, postpartum haemorrhage and wound dehiscence.^38 39 64^ The most reported cause of maternal mortality was ruptured uterus.^15 18 25 26 30 32 35 61^

## Discussion

Although it varies by country, our review found that district hospitals provide mainly emergency obstetric and general surgery and, in some cases, elective surgery covering a wide range of surgical specialties. The literature reports that, most often, surgical procedures are done under general or spinal anaesthesia.

Many of the procedures performed are by trained non-physician clinicians and medical officers. There is a paucity of data regarding quality of surgical care, surgical volume and perioperative morbidity and mortality.

Our study is a snapshot of two decades of published surgical activity in district hospitals. We had aimed to compare it with other similar studies, but we found that none was appropriate, either in terms of similar methodology, scope or research question. We suggest that there is a challenge and an imperative for district hospitals to collect and publish information on key indicators so that standards can be measured, and quality improvement can be encouraged. Our preference is to start with the agreed indicators recommended by the Lancet Commission on Global Surgery.^6^

Based on the findings of this study, our recommendation is for Ministries of Health in sub-Saharan Africa to consider policies and resources that prioritise quality improvement practices for district-level surgical care in the region, including standardisation and quality improvement in surgical information systems.

We have reflected on the strengths and limitations of this study. When we designed the study protocol, we followed the WHO terminology regarding ‘district hospitals’.^28^ If we had used the term ‘rural’ in our search strategy, it would have added confusion as it includes a variety of structures, many of which are health centres or dispensaries without in-patient facilities. However, we could have missed relevant literature that did not specifically use the terminology ‘district hospital’ but instead used other terms such as ‘rural hospital’. Our search strategy used the term ‘Africa south of the Sahara’ and this may have omitted studies that mentioned a country name but not the term ‘Africa or ‘south of the Sahara’. Our search included English, French and Portuguese texts but work in other languages may have been omitted. We sought to minimise subjectivity; therefore, two authors independently conducted the article selection and data extraction. Our study is prone to some publication bias as it is based only on information present in the published literature, and findings those were never published will have been omitted. It is unlikely that institutions those are not actively auditing activity would present it for publication, and even those district hospitals that do collect information of relevance to this study are unlikely to submit it for publication.^60^ Indeed, these institutions face several barriers to publish, such as the cost of submissions to journals and overwhelming clinical demands reduce the time available for academic work. Furthermore, there is usually a cost of running trials/data collection and, if the research is published, there is often a pay wall burden resulting in a limited distribution of results for the readership in LMICs. Finally, our results are limited by the heterogeneity in how articles reported indicators such as personnel training level, surgical volume (in terms of minor and major operations), complications, outcomes and perioperative mortality. Some articles used surgical indicators, but these were usually nonstandard, and the core surgical indicators (geographical access to surgical care within 2 hours, surgical providers’ density, total operative volume, in-hospital postoperative mortality and impoverishing and catastrophic cost burden) recommended by the Lancet Commission on Global Surgery^6^ were not routinely used. However, this is the first review to systematically assess the literature for the scope, volume and quality of surgery in district hospitals in sub-Saharan Africa, to our knowledge.

## Conclusion

District hospitals play a significant role in surgical care in sub-Saharan African countries. This scoping review has provided a broad but incomplete data set on the current provision of surgical care in district hospitals in sub-Saharan Africa as reported in published studies. Based on the findings of this study, we recommend to researchers to conduct more primary surgical systems research in district hospitals to report on surgical activity, using core indicators suggested by the Lancet Commission on Global Surgery that will improve comparability.

## Supplementary Material

Reviewer comments

Author's manuscript
