# Supplementary material for: Surgical care in district hospitals in sub-Saharan Africa: a scoping review
Source: BMJ Open. 2021 Mar 25;11(3):e042862. doi: 10.1136/bmjopen-2020-042862 (PMC7996654; doi:10.1136/bmjopen-2020-042862)
Supplement: Supplementary data [file bmjopen-2020-042862supp001.pdf]

Data extraction form for the scoping review

Surgical care in District Hospitals in sub Saharan Africa: a Scoping Review

* Required

1. First Author *

2. Year of publication *

3. Year of data collection (latest date to be chosen) *

4. Type of study/Research Method *

Bentounsi Z, *et al. BMJ Open* 2021; 11:e042862. doi: 10.1136/bmjopen-2020-042862

5. Country *

Check all that apply.

- [ ] Angola
- [ ] Burundi
- [ ] DRC
- [ ] Cameroon
- [ ] Central African Republic
- [ ] Chad
- [ ] Republic of the Congo
- [ ] Equatorial Guinea
- [ ] Gabon
- [ ] Kenya
- [ ] Nigeria
- [ ] Rwanda
- [ ] Sao Tome
- [ ] Tanzania
- [ ] Uganda
- [ ] Sudan
- [ ] South Sudan
- [ ] Djibouti
- [ ] Eritrea
- [ ] Ethiopia
- [ ] Somalia
- [ ] Botswana
- [ ] Comores
- [ ] Lesotho
- [ ] Madagascar
- [ ] Malawi
- [ ] Mauritius
- [ ] Mozambique
- [ ] Namibia
- [ ] Seychelles
- [ ] South Africa
- [ ] Swaziland
- [ ] ZAMBIA
- [ ] Zimbabwe
- [ ] Benin
- [ ] Mali
- [ ] Burkina Faso
- [ ] Cape Verde

- [ ] Ivory Cost
- [ ] Gambia
- [ ] Ghana
- [ ] Guinea
- [ ] Guinea-Bissau
- [ ] Liberia
- [ ] Mauritania
- [ ] Niger
- [ ] Senegal
- [ ] Sierra Leone
- [ ] Togo

Other: [ ] ______________________

6. Specialty *

Check all that apply.

- [ ] Obstetrics and Gynecology
- [ ] General Surgery
- [ ] Trauma and Orthopedics
- [ ] Head and Neck
- [ ] Urology
- [ ] Anesthesia
- [ ] Neurosurgery
- [ ] Paediatric surgery

Other: [ ] ______________________

7. Name of surgical procedures (please write all procedures mentioned) or write NA if none *

____
____
____
____
____

Bentounsi Z, *et al. BMJ Open* 2021; 11:e042862. doi: 10.1136/bmjopen-2020-042862

8. Surgical Provider *

Check all that apply.

- [ ] Specialist Surgeon (including OBGY)
- [ ] Medical Officer/GP
- [ ] Non Physician Clinician
- [ ] Nurse/Midwife
- [ ] Not specified

Other: [ ] ______________________

9. Type of Anaesthesia *

Check all that apply.

- [ ] General
- [ ] Regional Block
- [ ] Local
- [ ] Spinal
- [ ] Not specified

10. Anaesthesia Provider *

Check all that apply.

- [ ] Physician (anaesthesiologist)
- [ ] Non Physician- trained
- [ ] Non Physician- untrained
- [ ] Not specified
- [ ] Medical officer/Physician non anaesthesiologist

11. Number of operations per year/period (if available)

____

12. Catchment population (if available)

____

Bentounsi Z, *et al. BMJ Open* 2021; 11:e042862. doi: 10.1136/bmjopen-2020-042862

13. Equipment (if in a table, just write "table X" here)

14. Patients Outcomes (complications, survival...)

15. What do authors use as indicators for safety? (just list them)

16. What do authors use as indicators for surgical capacity?

17. Was the surgical care provided as a result of a temporary surgical mission or international partnership? *

Mark only one oval.

- Yes
- No
- It is unclear

Bentounsi Z, *et al. BMJ Open* 2021; 11:e042862. doi: 10.1136/bmjopen-2020-042862

18. Characteristics of the District Hospital(s) if given: number of bed, number of staff

19. Other important results

20. Recommendations made by authors

This content is neither created nor endorsed by Google.

Google Forms
