# Supplementary material for: Surgical care in district hospitals in sub-Saharan Africa: a scoping review
Source: BMJ Open. 2021 Mar 25;11(3):e042862. doi: 10.1136/bmjopen-2020-042862 (PMC7996654; doi:10.1136/bmjopen-2020-042862)
Supplement: Supplementary data [file bmjopen-2020-042862supp002.pdf]

Surgical Operations mentioned:

First Author	Country	Name of surgical procedures mentioned in text
Compaoré	Burkina Faso	caesarean section
Dossche	Burkina Faso	Total hip replacement, bipolar hemiarthroplasty
Ouedraogo C.M	Burkina Faso	Caesarean section
Ouedraogo	Burkina Faso	Caesarean section, laparotomy, cervical and perineal surgical repair
Kenfack	Cameroon	laparotomy for ectopic pregnancy
Ngaroua	Cameroon	Optical internal urethrotomy
Ramos	Ethiopia	subtotal thyroidectomy, hemi-thyroidectomy
Abdullah	Ghana	caesarean section, hernia repair, evacuation of retained products of conception from the uterus, Abdominal hysterectomy, Arthrotomy, Hand surgery, laparotomy, amputation, appendectomy, salpingectomy (e.g. ectopic pregnancy), internal fixation of fracture, other uterine procedure
Choo	Ghana	caesarean section, laparotomy, hernia repair, appendectomy, abortion
Choo	Ghana	Not specified
Damien	Ghana	caesarean sections; hernia repair and other laparotomies
Gyedu	Ghana	obstetric fistula repair, hysterectomy for uterine rupture, or intractable postpartum haemorrhage, bowel obstruction, construction of colostomy, trauma laparotomy, and skin grafting
Hall	Ghana	Caesarean section
Luo	Ghana	Caesarean section, hysterectomy
McCord C	Ghana	NA
Mehtsun	Ghana	Caesarean section, hernia, ulcer requiring debridement, cellulitis requiring debridement, abscess, trauma, burn
Smiley	Ghana	breast lumpectomy, circumcision, hernia repair, caesarean section,
Stewart	Ghana	basic general surgery, major general surgery, vascular repair, skin grafting, closed reduction, skin or skeletal traction, external fixation, internal fixation, basic neurosurgery, major neurosurgery, spine fixation
Koigi-Kamau R	Kenya	Caesarean section
Nordberg	Kenya	Caesarean section, hernia repair, exploratory laparotomy, operation on stomach, tubal ligation, prostatectomy, amputation, hydrocele, hysterectomy, myomectomy, adnexectomy, mastectomy, appendectomy, ectopic pregnancy, cleft lip/palate, club foot, thyroidectomy, open reduction of fracture, eyes/lens removal,

		orchidectomy, haemorrhoidectomy, splenectomy, vesico-vaginal fistula repair.
Sherman	Liberia	incision and drainage of abscesses, suturing, dilatation and curettage, male circumcision, burn care, wound debridement, appendectomy, caesarean section, hernia repair, laparotomy, cricothyroidotomy, chest tube insertion , foreign body removal, congenital hernia repair, contracture release, closed fracture repair, open fracture repair, management of osteomyelitis, amputation, biopsy, tubal ligation, dilation, curettage.
Fenton	Malawi	Caesarean section
Gajewski	Malawi	hysterectomy, ectopic pregnancy–salpingectomy, inguinal herniotomy, herniorrhaphy, prostatectomy, appendicectomy, repair of stab wounds, repair of gastrointestinal perforation, gastrointestinal resection with anastomosis or colostomy, other laparotomy, amputation of arm or leg, hydrocele excision and repair, cataract removal
gajewski	Malawi	hernia repair
Gajewski J	Malawi	not provided
Harfouche	Malawi	Caesarean section
Henry J	Malawi	resuscitation, cricothyroidotomy, tracheostomy, chest tube insertion, burn management, skin grafting, contracture release, splinting and casting, traction for closed fracture, open fracture, osteomyelitis management, amputation, appendectomy, hernia repair, bowel resection, laparotomy, biopsy, cholecystectomy,
lavy	Malawi	Dilatation and curettage, Caesarean section, Suture of wound, Manipulation of fracture, Hernia repair, Debridement of wound, Laparotomy, osteomyelitis surgery, Arthrotomy, Debridement of open fracture, Insertion of skeletal traction pin, Tenotomy, Skin graft, Osteotomy, Internal fixation of fracture, Club foot correction, Burr hole
Van Amelsfoort	Malawi	NA
van den Akker	Malawi	uterine rupture
Ottaway	Namibia	Caesarean section
Sani	Niger	Emergency: caesarean section, laparotomy for acute peritonitis, repair of strangulated hernia, laparotomy for intestinal obstruction, Elective: Hydrocele, Inguinal hernia, Bladder stone, Umbilical hernia, Lipoma, Ovarian Cyst, uterine fibroma, ectopic testicle, uterine prolapse
Mpirimbanyi C	Rwanda	The most common primary diagnoses were soft tissue infections followed by acute abdominal conditions, complicated hernias, urological emergencies and thoracic emergencies. Within soft tissue infections, abscesses followed by pyomyositis were the most common diagnoses. Bowel obstruction followed by volvulus were the most common type of acute abdominal conditions.

Muhirwa	Rwanda	excision of cysts, lipomas, keloids, and masses abscess, incision and drainage hernia/hydrocele repair, laparotomy, closed reduction of fracture, biopsy, amputation, Haemorrhoidectomy, mastectomy
Nkurunziza	Rwanda	Lower (uterine) segment caesarean section
Notrica	Rwanda	NA
Petroze	Rwanda	caesarean section, herniorrhaphy, amputation, closed fracture repair, chest tube insertion, laparotomy, emergency airway, wound debridement,
Petroze	Rwanda	NA
Le Brun	Rwanda, Uganda, Ethiopia, Liberia	NA
De Brouwere	Senegal	Caesarean section
Henry	SSA	Caesarean section symphysiotomy, assisted or manipulative delivery. ERPC, B-Lynch suture, repair of uterine perforation. Incision and drainage of abscess, fasciotomy, dental extraction, tympanotomy, bone drilling, arthrotomy. Debridement, haemostasis, suturing, escharotomy, skin grafting. Management of head injury, cranial burr holes, elevation of depressed skull fracture. Management of compromised airway, tracheostomy, cricothyroidotomy, removal of foreign body. Intercostal drainage, thoracostomy. Emergency laparotomy including appendectomy. Reduction of fractures and dislocations, casting and splinting, external Fixation. Amputations. Suprapubic catheterization. Hernia repair. Cataract extraction and IOL insertion. Casting and splinting, tenotomy. Cleft lip repair.
grimes	Sub Saharean Africa	Hysterectomy, Ectopic Pregnancy, Tubal Ligation, Caesarean section, Uterine evacuation, Wound-related procedures, Herniorrhaphy, Laparotomy, Appendectomy, Skin graft, Prostatectomy, Hydrocele, Strangulated hernia, Chest drain, Burr holes/craniotomy, Thoracotomy, Incision and drainage of abscess, Circumcision, Amputation
Fehr	Tanzania	Caesarean sections were the surgical interventions performed most frequently, followed by other gynaecological procedures.
Fehr	Tanzania	Caesarean Section, hysterectomy, laparotomy, hernia repair, hydrocele repair, orthopaedics
McCord	Tanzania	emergency obstetrical operations
Gajewski, Jakub	Tanzania, Malawi, ZAMBIA	Anaesthesia care
Galukande	Tanzania, Uganda, Mozambique	caesarean section, wound related procedures, hernia repair, uterine evacuation, laparotomy, open fracture, tubal ligation, hysterectomy, hydrocele, circumcision, appendectomy, biopsy of lump, salpingectomy, limb amputation, skin graft, extraction of placenta, cystostomy, osteotomy/sequestrectomy, foreign body removal, orchiectomy, haemorrhoidectomy, splenectomy, mastectomy, prostatectomy, cervical tear repair, vesico-vaginal fistula repair

dresser	Uganda	lacerations, hernias, gastrointestinal perforation, bowel obstruction, ectopic pregnancy, hemoperitoneum, fracture, peritonitis, burns, splenic lacerations, degloving injury.
Lofgren	Uganda	Caesarean delivery, Evacuation of uterus, Herniorrhaphy, Explorative laparotomy, hysterectomy, Salpingo/oophorectomy or removal of tubal/ovarian mass, appendectomy, tubal ligation, repair of cleft palate, laparotomy for ectopic pregnancy, circumcision, repair of cuts and tears, incision and drainage of abscess, reduction of fracture, excision of granuloma of the umbilical cord, excision of mass, incision of tongue tie, reduction of dislocated joint, biopsy, removal of foreign body. The most invasive orthopaedic procedure was insertion of attraction pin in the tibial tuberosity in femur fractures
Cheelo	ZAMBIA	Caesarean section and general surgical procedures
Lonnée,	Zimbabwe	NA
RUTGERS	Zimbabwe	caesarean section
