# Supplementary material for: Surgical care in district hospitals in sub-Saharan Africa: a scoping review
Source: BMJ Open. 2021 Mar 25;11(3):e042862. doi: 10.1136/bmjopen-2020-042862 (PMC7996654; doi:10.1136/bmjopen-2020-042862)
Supplement: Author's manuscript [file bmjopen-2020-042862.draft_revisions.pdf]

BMJ Open is committed to open peer review. As part of this commitment we make the peer review history of every article we publish publicly available.

When an article is published we post the peer reviewers' comments and the authors' responses online. We also post the versions of the paper that were used during peer review. These are the versions that the peer review comments apply to.

The versions of the paper that follow are the versions that were submitted during the peer review process. They are not the versions of record or the final published versions. They should not be cited or distributed as the published version of this manuscript.

BMJ Open is an open access journal and the full, final, typeset and author-corrected version of record of the manuscript is available on our site with no access controls, subscription charges or pay-per-view fees (http://bmjopen.bmj.com).

If you have any questions on BMJ Open's open peer review process please email info.bmjopen@bmj.com

BMJ Open

Surgical care in District Hospitals in sub-Saharan Africa: a Scoping Review

Journal:	*BMJ Open*
Manuscript ID	bmjopen-2020-042862
Article Type:	Original research
Date Submitted by the Author:	16-Jul-2020
Complete List of Authors:	Bentounsi, Zineb; University of Oxford,<br>Sheik-Ali, Sharaf; University of Oxford<br>Le, Grace; Nuffield Department of Orthopaedics, Rheumatology and Musculoskeletal Sciences (NDORMS),<br>Lavy, Chris; Nuffield Department of Orthopaedics, Rheumatology and Musculoskeletal Sciences (NDORMS), ,
Keywords:	SURGERY, HEALTH SERVICES ADMINISTRATION & MANAGEMENT, ANAESTHETICS

SCHOLARONE™
Manuscripts

I, the Submitting Author has the right to grant and does grant on behalf of all authors of the Work (as defined in the below author licence), an exclusive licence and/or a non-exclusive licence for contributions from authors who are: i) UK Crown employees; ii) where BMJ has agreed a CC-BY licence shall apply, and/or iii) in accordance with the terms applicable for US Federal Government officers or employees acting as part of their official duties; on a worldwide, perpetual, irrevocable, royalty-free basis to BMJ Publishing Group Ltd ("BMJ") its licensees and where the relevant Journal is co-owned by BMJ to the co-owners of the Journal, to publish the Work in this journal and any other BMJ products and to exploit all rights, as set out in our licence.

The Submitting Author accepts and understands that any supply made under these terms is made by BMJ to the Submitting Author unless you are acting as an employee on behalf of your employer or a postgraduate student of an affiliated institution which is paying any applicable article publishing charge ("APC") for Open Access articles. Where the Submitting Author wishes to make the Work available on an Open Access basis (and intends to pay the relevant APC), the terms of reuse of such Open Access shall be governed by a Creative Commons licence – details of these licences and which Creative Commons licence will apply to this Work are set out in our licence referred to above.

Other than as permitted in any relevant BMJ Author's Self Archiving Policies, I confirm this Work has not been accepted for publication elsewhere, is not being considered for publication elsewhere and does not duplicate material already published. I confirm all authors consent to publication of this Work and authorise the granting of this licence.

For peer review only

Surgical care in District Hospitals in sub-Saharan Africa: a Scoping Review

Authors:

Zineb Bentounsi, corresponding author, Botnar Research Centre, NDORMS, University of Oxford, Old Road, OX3 7LD, Oxford, UK. 07727064357, zineb.bentounsi@ndorms.ox.ac.uk
Sharaf Sheik Ali, sharaf.sheik-ali@medsci.ox.ac.uk Botnar Research Centre, NDORMS, University of Oxford, UK.
Grace Le, grace.le@ndorms.ox.ac.uk Botnar Research Centre, NDORMS, University of Oxford, UK.
Chris Lavy, chris.lavy@ndorms.ox.ac.uk Botnar Research Centre, NDORMS, University of Oxford, UK.

For peer review only

Keywords: Global Surgery, Surgery, District Hospitals, Africa

Word count: 2768

Abstract:

Objective: To summarize the body of literature existing on the provision of surgical and anaesthesia care in district hospitals in sub-Saharan Africa.

Design: Scoping Review

Setting: District hospitals in sub-Saharan Africa

Data sources: PubMed and Ovid EMBASE from January 2000 to December 2019

Study selection: Studies were included if they contained information about types of surgical procedures performed, number of operations per year, types of anaesthesia delivered, cadres of surgical/anaesthesia providers and/or patients' outcomes.

Results: The 52 articles included in analysis provided information about 16 countries. District hospitals were a group of diverse structures ranging from 21 to 371 beds. The 3 most frequently reported procedures were caesarean section, laparotomy and hernia repair but a wide range of orthopaedics, plastic surgery and neurosurgery procedures were also mentioned. The number of operations performed per year per district hospital ranged from 239 to 5233. The most mentioned anaesthesia providers were non-physician clinicians trained in anaesthesia. They deliver mainly general and spinal anaesthesia. Depending on countries, articles referred to different surgical care providers: specialist surgeons, medical officers and non-physician clinicians. 15 articles reported peri-operative complications among which surgical site infection was the most frequent. 15 articles reported perioperative deaths of which the leading causes were sepsis, haemorrhage and anaesthesia complications.

Conclusion: District hospitals play a significant role in sub-Saharan Africa, providing both emergency and elective surgery. Most procedures are done under general or spinal anaesthesia, often administered by non-physician clinicians. Depending on countries, surgical care may be provided by medical officers, specialist surgeons and/or non-physician

For peer review only - http://bmjopen.bmj.com/site/about/guidelines.xhtml

clinicians. Research on safety, quality and volume of surgical and anaesthesia care in this setting is scarce and more attention to these questions is required.

Article Summary:

Strengths and limitations:

- This is the first review to systematically assess the literature for the scope, volume and quality of surgery in district hospitals in sub-Saharan Africa.
- Article selection and data extraction were conducted independently by two authors
- Our search was conducted in English, French and Portuguese.
- This study is prone to publication bias as it is based on information present in published literature.
- There was a lot of heterogeneity in how articles reported surgical volume and quality.

Introduction:

The burden of surgical disease in Sub-Saharan Africa (SSA) is estimated at 38 disability adjusted life years (DALYs) lost per 1,000 population. District hospitals bear a large burden of this surgical care delivery(1). According to The World Health Organization (WHO), district hospitals are defined as the first level of healthcare facilities that provide in-patient surgery and anaesthesia(2). These hospitals which mainly serve rural populations, face challenges in terms of workforce, infrastructure and other essential resources. In 2010, it has been estimated that there was only 1 surgeon for 25 million people in rural SSA(3) and although training programs in the region are increasing the number of surgeons, the main burden in several countries remains on non-physician clinicians to urgently manage surgical cases(4).

District hospitals (DHs) offer a unique opportunity to improve access to surgical care in SSA as they are the first point of access to surgical care for patients and relieve referral hospitals from undue burden, allowing them to focus on complicated cases. Delivering timely surgical care in DHs has been shown to be highly cost effective(5) which should provide encouragement for governments and global health programs to invest in them. Moreover, the literature calls to consider DHs as a central component in strengthening surgical care in SSA(6)(7).

A key factor in identifying the most efficient way to address the surgical disease burden in DHs of the region is to review the scope of surgical care delivery in DHs. While the literature on district hospital surgery in SSA tends to focus on improving the current training and surgical delivery in individual hospitals, we found limited literature on scope, volume and quality of surgical care in district hospitals across the region.

In view of the number of existing studies in this field and range of methodology used, the authors undertook a scoping review of surgical care delivery in district hospitals in SSA, aiming to provide a general overview of the reported current surgical capacity and delivery, advance current knowledge and suggest targets for policy/ further development and research within the region of SSA.

For peer review only - http://bmjopen.bmj.com/site/about/guidelines.xhtml

Methods:

This scoping review follows the five steps methodology designed by Arksey and O'Malley(8) that offers a rigorous framework for reporting findings. We have also considered the recommendations of Levac, Colquhoun and O'Brien(9) that supplement this framework.

1. Identifying the research question

To guide our search of the literature, we defined the following questions:

Primary outcome:
In district hospitals in sub Saharan Africa, what types of surgical procedures are provided?

Secondary outcomes:
In district hospitals in sub-Saharan Africa:
❖ How many surgical procedures are performed per year?
❖ Are there any measures of quality, safety or outcomes of surgery?
❖ Who provides anaesthesia and/or surgery?
❖ What type of anaesthesia is delivered?

2. Identifying relevant studies

PubMed and Ovid EMBASE were searched on 18/12/19 to retrieve literature about surgery in district hospitals in sub-Saharan Africa. The search strategy combined the exploded thesaurus terms "surgery", "surgical procedures, operative" and "specialties, surgical" with the exploded thesaurus terms "hospitals, district" and "Africa south of the Sahara". References were searched in English, French and Portuguese. We decided to limit the search to references published after January 1st, 2000. Google Scholar and the Cochrane Library were also searched, but as they did not contribute any relevant new references, the results were not included for analysis.

3. Study selection

The most important inclusion criteria was the setting: a district hospital in sub-Saharan Africa. We then considered if the article provided information about either type of surgical procedures performed, number of operations per year, types of anaesthesia delivered, cadres of surgical/anaesthesia providers and/or patients outcomes. They were excluded if the setting was unclear or was not a district hospital, which was defined as the lowest level of healthcare providing in-patient surgical services, and if they were strictly about costs or burden of disease. Articles for which the full text could not be retrieved were excluded. All surgical procedures were included except dental, ophthalmic and non-surgical obstetric procedures.

For peer review only - http://bmjopen.bmj.com/site/about/guidelines.xhtml

The screening process was done independently by Z.B and G.L using the web application Rayyan(10). Conflict was resolved by dialogue between Z.B, G.L and C.L. The process of selection followed the Preferred Reporting of Items for Systematic Reviews and Meta-Analyses (PRISMA) Statement.

4. Charting the data

The data charting form was developed by Z.B after consultation with all other authors. The form was piloted and then updated iteratively during the extraction process. Data was extracted independently by Z.B and S.S.

5. Collating, summarizing and reporting the results

A descriptive numerical summary of the data was produced by S.S using Excel. Graphs were also produced in Excel. A thematic analysis was performed by Z.B in NVivo(11). Results were reported to answer the primary and secondary outcomes of the review. Implications for research and policy were discussed and reported by all authors.

Results and Discussion:

In this section, results and discussion are simultaneously presented. Characteristics of the included articles and an analysis of the district hospitals are first explored. Thereafter, we present our findings on the scope and volume of surgery, types of anaesthesia, surgical and anaesthesia providers, and finally safety and quality of care.

1. Study Selection

We identified 302 articles and after removing duplicates 267 articles remained. The titles and abstracts were screened, and 167 articles were excluded. Then, full texts of the remaining 100 articles were assessed and 48 were excluded. Therefore, 52 articles were included for analysis. A PRISMA diagram is presented in Figure 1.

Figure 1: PRISMA diagram illustrating the selection process

2. Study Characteristics

Most articles were cross-sectional surveys, some presented data from the late 1990s but were actually published in 2000 or later (see Supplementary Table 1). Most articles described the surgical care provided by staff based at the hospital, however, some reported external support from Non-Governmental Organisations (NGO) or visiting surgical teams (12–16)

This review covered 16 countries with a few articles presenting data from multiple countries(4,17,18) and others having a regional perspective(19,20). Ghana, Malawi and Rwanda were the three countries most represented in the articles selected, partly because of research projects in collaboration with researchers from High Income Countries (HIC) (Table 1)

Table 1: Distribution of articles per country

For peer review only

For peer review only - http://bmjopen.bmj.com/site/about/guidelines.xhtml

Country	Number of articles
Burkina Faso	3
Cameroon	2
Ethiopia	2
Ghana	11
Kenya	2
Liberia	2
Malawi	10
Mozambique	1
Namibia	1
Niger	1
Rwanda	7
Senegal	1
Sub Saharan Africa	2
Tanzania	5
Uganda	4
Zambia	2
Zimbabwe	2

3. Characteristics of district hospitals:

District hospitals described in this review included a variety of structures. In our review, the two smallest DH had 21 beds reported in Liberia and Ghana by Sherman et al(21) and Choo et al(22) respectively. The biggest DH had 371 beds and was reported by Fehr et al in Tanzania(23). District hospitals served rural or urban populations, with some serving both urban and rural areas. Although most were owned by the government, mission hospitals also featured in several papers. On some occasions, hospitals received support from visiting surgeons or NGOs, e.g. in Rwanda, government owned district hospitals received support from Partners in Health(14).

4. Scope of surgical operations

The majority of articles mentioned obstetrics/gynaecology and general surgery operations (Figures 2 and 3). Indeed, the three most frequently reported procedures were caesarean section, laparotomy and hernia repair. However, a wide range of other surgical procedures were mentioned across the literature, these were mainly in orthopaedics, plastic surgery or neurosurgery and may have involved visiting specialists (Table 2). District hospitals reported in the literature handled both emergency procedures such as caesarean sections, appendicectomies and treatment of burns; and elective procedures such as hernia repairs and some even more complex procedures such as direct vision internal urethrotomy(24) and hip replacement(13). Despite the high prevalence of traumatic brain injury in sub Saharan Africa(25) and the fact that cranial burr holes are included in the WHO book "Surgical Care at the District Hospital"(26), only three articles mentioned burr holes.

Figure 2: Number of articles reporting obstetrics and gynaecology operations

For peer review only - http://bmjopen.bmj.com/site/about/guidelines.xhtml

Figure 3: Number of articles reporting general surgery operations

Table 2: Number of articles reporting non obstetrics, non-general surgery operations

Surgical Procedures	Number of articles
Orthopaedics	
Amputation	10
Open Fracture repair	8
Closed Fracture repair	5
Arthrotomy	3
Osteotomy	2
Total Hip replacement	1
Bipolar Hemiarthroplasty	1
Neurosurgery	
Major neurosurgery	1
Burr holes	3
Plastics	
Skin grafting	7
Debridement	5
Burn	4
Cleft lip/palate surgery	3
Contracture release	2
Escharotomy	1

5. Surgical volume

Only 13 articles provided enough data to be able to estimate the number of operations per year per district hospital performed. We didn't always have enough information to discriminate between major and minor operations, so we considered all operations combined. Using a simple formula; the number of operations, period of years and number of district hospitals, an average of the number of operations per district hospital per reported year was calculated (Table 3). Galukande et al(4) and LeBrun et al(18) contributed several reports on different district hospitals while Van Amelsfoort et al(27) contributed two reports on the same district hospital during two different time periods.

As seen on tables 3 and 4, the surgical volume of district hospitals varied from circa 200 to circa 5000 operations per year, with the majority of hospitals performing between 1001 and 5000 operations per year. Clear limitations to the use of this data is the sample size and selection bias. Of the 13 studies, only six analysed more than five district hospitals in a given area. This data, therefore, cannot solely be used as an indicator for the productivity of district hospitals in the countries reported, rather it provides a broad reference number and supplements our general analysis of district hospitals in sub-Saharan Africa as reported in the literature.

For peer review only - http://bmjopen.bmj.com/site/about/guidelines.xhtml

Table 3: Surgical volume (all reported cases) in number of operations per year per district hospital

First author	Total number of operations	Years	Number of district hospitals	Estimated number of operations per district hospital per year *(to the nearest unit)*
Abdullah~ (28)	-	1	10	774
Damien (29)	1,391	5	1	278
Galukande (4)	980	1	1	980
Galukande (4)	2,045	1	1	2,045
Galukande (4)	601	1	1	601
Galukande (4)	256	1	1	256
Galukande (4)	1,484	1	1	1,484
Galukande (4)	248	1	1	248
Galukande (4)	239	12	1	239
Galukande (4)	1,835	1	1	1,835
Gyedu (30)	143,750	1	48	2,995
Lofgren $ (31)	-	-	2	5,018
Lavy (32)	25,053	1	21	1,193
LeBrun* (18)	-	1	1	1,296
LeBrun* (18)	-	1	1	2,052
LeBrun* (18)	-	1	1	696
LeBrun* (18)	-	1	1	538
Nordberg~ (33)	-	1	5	5,233
Notrica* (34)	-	-	-	2,052
Ottaway (35)	737	0.58	4	316
Petroze (36)	71,432	1	41	1,742
Sani (37)	544	1	3	181
Van Amelsfoort (27)	19,644	1	17	1,156
Van Amelsfoort (27)	18,524	1	17	1,090

* Reported in the article as average number per year per district hospital
~ The article reported numbers for both major and minor operations and we calculated the total number of procedures.
$ This article reported that the number of major operations was estimated over a 4 month period while the number of minor operations was estimated over a 3 month period.

Table 4: Number of publications reporting the number of operations per year per district hospital

Number of operations on average per district hospital over 1 year	Number of publications
<100	0

For peer review only - http://bmjopen.bmj.com/site/about/guidelines.xhtml

100-500	6
501-1000	5
1001-5000	11
>5000	2

6. Types of anaesthesia

Out of 52 articles, 16 contained information about the type of anaesthesia used in the district hospitals studied. General anaesthesia has been mentioned by 15 articles(9,11,13,17,21–32) and spinal anaesthesia by 10 articles(13,16,21,22,31,35,39–41,44). Local anaesthesia and regional blocks have been mentioned by 4 articles(14,16,21,31).

7. Surgical and anaesthesia providers

In different countries, different cadres of healthcare professionals are responsible for providing surgical care in district hospitals. In Ethiopia, Ramos et al reported the presence of a specialist surgeon(12). In Ghana(22,28,30,45–49), Kenya(33), Rwanda(14–16,34,36,42) and Zimbabwe(43) medical officers worked alongside specialist surgeons or obstetrician/gynaecologists to provide surgical care. In Namibia(35) and Niger(37), the presence of medical officers as sole surgical providers was reported. Non-physician clinicians provided surgical care in Burkina Faso(13,41,50), Liberia(21), Malawi(38,44,51–53), Senegal(54), Tanzania(17,55), Uganda(31,56) and Zambia(57). In our review, there was no clear majority in terms of the number of articles mentioning each cadre (Table 5).

Similarly, anaesthesia is the responsibility of different cadres depending on which country. Articles reported the presence of a physician anaesthetist in district hospitals in Burkina Faso(13) and Ghana(49). Medical officers providing anaesthesia on their own have been reported in Namibia(35). Otherwise, the most mentioned anaesthesia providers are non-physician clinicians trained in anaesthesia (Table 6). Their presence has been reported in Burkina Faso(50), Ethiopia(12), Ghana(22), Liberia(21), Malawi(44)(20), Rwanda(34)(36), Senegal(54), Tanzania(17), Uganda(31), Zambia(57) and Zimbabwe(43)(39). There are also untrained non-physician clinicians providing anaesthesia in Liberia(21), Malawi(44)(20), Rwanda(34)(36), Zimbabwe(43)(39) alongside trained personnel.

Table 5: Number of publications reporting surgical providers

Surgical Providers	Number of Publications
Specialist Surgeon/Obstetrician	5
Specialist Surgeon/Obstetrician & Medical Officers	9
Medical Officers	8
Medical Officers & Non-Physician Clinicians	4
Specialist Surgeon/Obstetrician & Non-Physician Clinicians	1
Non-Physician Clinicians	5
Specialists & Medical Officers & Non-Physician Clinicians	8

For peer review only - http://bmjopen.bmj.com/site/about/guidelines.xhtml

Table 6: Number of publications reporting anaesthesia providers

Anaesthesia Providers	Number of Publications
Physician Anaesthetists	2
Medical Officers	1
Medical Officer & Non-Physician Clinicians	1
Non-Physician Clinicians (trained)	10
Non-Physician Clinicians (trained & untrained)	6

8. Quality of care and surgical outcomes

8.1. Quality of care

Of all articles in our review, only nine articles evaluated the quality of care as part of their study. Choo et al surveyed 10 district hospitals in Ghana about quality improvement activities(58). None of the hospitals surveyed had a “comprehensive quality improvement programme” and the only quality improvement activity related to surgery was found in one hospital and related to sterilisation of instruments(58). Other authors echoed these findings by reporting a lack of clinical guidelines for surgery, anaesthesia and labour management(21,50,59) and a lack of staff trained in using them when they were available(50).

To evaluate the quality of caesarean sections, Harfouche et al measured the “decision to incision time” in a district hospital in Malawi and found that it reached “1.69 hours per caesarean section, far exceeding the recommended 30 minutes”(60). They explained their findings by a “lack of operating theatre space and high patient volume” but also by an inefficient system to transfer patients to the operating theatre(60).

Another major challenge to quality care in district hospitals in sub-Saharan Africa is the shortage in trained surgical and anaesthesia staff. Stewart et al(61) explained that shortage in staff resulted in unavailability of service during nights and weekends. Choo et al found that medical officers in Ghana had limited exposure to surgery during their training(22). Harfouche et al reported that while clinical officers performed most caesarean sections at district level in Malawi, they were not sufficiently trained in performing emergency hysterectomies(60). Positive outcomes were observed in cases where trained staff was present: Hall et al reported an “association between the presence of OBGYNs in district hospitals in Ghana and an increase in evidence-based maternal care practices, as well as moderate improvement in maternal mortality and stillbirth rates”(45).

Some authors suggested interventions to improve the quality of care. Van den Akker et al suggested audit as a “simple intervention requiring little technology” that is able to improve quality of care(62). They reported a decrease in 68% of uterine ruptures in a district hospital in Malawi after implementing recommendations inferred from an audit. Recommendations included training of staff, better documentation and involvement of hospital management(62).

For peer review only - http://bmjopen.bmj.com/site/about/guidelines.xhtml

Other authors insisted on the need for monitoring safety and quality of care in district hospitals as a first step toward improving quality(58)(31).

However, Smiley et al reported care of exceptional quality in a “63 bed district hospital” named the “Volta River Authority Hospital” in Ghana(49). In their study they surveyed staff and patients about their perception of quality of care and conducted process mapping of surgical and obstetric care. They found that “over 80% of employees across a variety of perioperative roles held a positive view of teamwork and safety climate within the institution” and “surgical patients who were surveyed gave similarly positive indications of overall satisfaction”(49). The perception of staff and patients was complemented by the observations of researchers during the process mapping. The WHO safety checklist was used in all operations witnessed and the authors reported the existence of a hospital wide morbidity and mortality conference as well as a safety training for new staff(49).

8.2. Surgical outcomes

Articles in our review used peri-operative complications and peri-operative mortality as indicators for safety. 15 articles reported information about peri-operative complications(13–16,23,35,40,44,51,52,59,60,62–64), the most reported being surgical site infection. 15 articles included information about peri-operative mortality(10-12,15,16,18,20,22,23,26,32,36,41,49,50), the most reported causes of death were sepsis, haemorrhage and anaesthesia complications.

In surgical obstetric care, the most reported complications were ruptured uterus, post-partum haemorrhage and wound dehiscence(44,60,62). The most reported cause of maternal maternity was ruptured uterus(15,18,20,23,26,30,32,49)

Strengths and Limitations:

This is the first review to systematically assess the literature for the scope, volume and quality of surgery in district hospitals in sub-Saharan Africa, to our knowledge. To minimise subjectivity, the article selection and data extraction were conducted independently by two authors. To mitigate a language bias, we did not limit our search to English, but included French and Portuguese. However, this study has some limitations. First, we could have missed literature that did not use the terminology ‘district hospital’ but instead used other terms such as ‘rural hospitals’ or ‘health centres’ to describe surgical activity in district hospitals. Second, our study is prone to some publication bias as it is based only on information present in published literature; the majority of district hospitals are not academic institutions and are thus unlikely to be represented in published literature. Finally, our results are limited by the heterogeneity in how articles reported indicators such as surgical volume and peri-operative mortality. If surgical indicators were used, these were usually non-standard, and the core surgical indicators recommended by the Lancet Commission on Global Surgery(65) were not routinely used.

Conclusion:

District hospitals play a significant role in surgical care in sub-Saharan African countries. Although it varies by country, our review found that district hospitals provide mainly

For peer review only - http://bmjopen.bmj.com/site/about/guidelines.xhtml

emergency obstetric and general surgery and, in some cases, elective surgery covering a wide range of surgical specialties. The literature reports that, most often, surgical procedures are done under general or spinal anaesthesia.

Many of the procedures performed are by trained non-physician clinicians and medical officers. There is a paucity of data regarding quality of surgical care, surgical volume and peri-operative morbidity and mortality. However, the most reported complications are infections and post-partum haemorrhage.

This scoping review has provided a broad data set on the current provision of surgical care in district hospitals in sub-Saharan Africa as reported in published studies. We recommend to researchers to conduct more primary surgical systems research in district hospitals to report on surgical activity, using core indicators suggested by the Lancet Commission on Global Surgery that will improve comparability.

Authors Contribution:

Zineb Bentounsi, MD, MSc: investigation, data curation, methodology, formal analysis, writing-original draft, writing-review and editing

Sharaf Sheik Ali, MBBS, BSc: investigation, formal analysis, writing- original draft, writing-review and editing

Grace Le, MA: project administration, investigation, writing-review and editing

Chris Lavy, MBBS, MD: conceptualization, supervision, writing-review and editing

Funding Statement:

No funding was received for this study.

Conflict of Interest:

Authors have no conflict of interest.

References:

1. Luboga S, Macfarlane SB, von Schreeb J, Kruk ME, Cherian MN, Bergström S, et al. Increasing access to surgical services in sub-saharan Africa: priorities for national and international agencies recommended by the Bellagio Essential Surgery Group. PLoS Med [Internet]. 2009/12/22. 2009 Dec;6(12):e1000200–e1000200. Available from: https://pubmed.ncbi.nlm.nih.gov/20027218
2. Mulligan J-A, Fox-Rushby JA, Adam T, Johns B, Mills A. Unit Costs of Health Care Inputs in Low and Middle Income Regions. 2003.
3. Pollock JD, Love TP, Steffes BC, Thompson DC, Mellinger J, Haisch C. Is it possible to train surgeons for rural Africa? A report of a successful international program. World J Surg. 2011 Mar 30;35(3):493–9.
4. Galukande M, von Schreeb J, Wladis A, Mbembati N, de Miranda H, Kruk ME, et al.

For peer review only - http://bmjopen.bmj.com/site/about/guidelines.xhtml

Essential surgery at the district hospital: a retrospective descriptive analysis in three African countries. PLoS Med. 2010 Mar;7(3):1–10.
5. Roberts G, Roberts C, Jamieson A, Grimes C, Conn G, Bleichrodt R. Surgery and Obstetric Care are Highly Cost-Effective Interventions in a Sub-Saharan African District Hospital: A Three-Month Single-Institution Study of Surgical Costs and Outcomes. World J Surg. 2016;40:14–20.
6. Rajbhandari R, McMahon DE, Rhatigan JJ, Farmer PE. The neglected hospital - The district hospital's central role in global health care delivery [Internet]. Vol. 382, New England Journal of Medicine. Massachussetts Medical Society; 2020 [cited 2020 Jun 3]. p. 397–400. Available from: http://www.nejm.org/doi/10.1056/NEJMp1911298
7. Grimes C, Lavy C. A plea for investment in district hospitals [Internet]. Vol. 376, The Lancet. Elsevier; 2010 [cited 2020 Jun 30]. p. 2073. Available from: http://www.thelancet.com/article/S0140673610622933/fulltext
8. Arksey H, O'Malley L. Scoping studies: towards a methodological framework. Int J Soc Res Methodol. 2005;8(1):19–32.
9. Levac D, Colquhoun H, O'Brien KK. Scoping studies: Advancing the methodology. Implement Sci. 2010;5(1):1–9.
10. Ouzzani M, Hammady H, Fedorowicz Z, Elmagarmid A. Rayyan-a web and mobile app for systematic reviews. Syst Rev [Internet]. 2016;5(1):210. Available from: http://dx.doi.org/10.1186/s13643-016-0384-4
11. QSR International. NVivo Qualitative Data Analysis Software- Version 12. 2018.
12. Ramos JM, Abate N, Reyes F, Belate W, Mohammed F, Gorgolas M. Thyroid surgery in a district hospital in Southern Ethiopia: experience from a rural center. 2013;37(7). Available from: http://www.ncbi.nlm.nih.gov/pubmed/23564217
13. Dossche L, Noyez JF, Ouedraogo W, Kalmogho E. Establishment of a hip replacement project in a district hospital in Burkina Faso: analysis of technical problems and peri-operative complications. Bone Joint J [Internet]. 2014;96(2):177–80. Available from: http://www.ncbi.nlm.nih.gov/pubmed/24493181
14. Muhirwa E, Habiyakare C, Hedt-Gauthier BL, Odhiambo J, Maine R, Gupta N, et al. Non-Obstetric Surgical Care at Three Rural District Hospitals in Rwanda: More Human Capacity and Surgical Equipment May Increase Operative Care. World J Surg. 2016;40(9):2109–16.
15. Nkurunziza T, Kateera F, Sonderman K, Gruendl M, Nihiwacu E, Ramadhan B, et al. Prevalence and predictors of surgical-site infection after caesarean section at a rural district hospital in Rwanda. Br J Surg [Internet]. 2019;106(2):e121–8. Available from: http://www.ncbi.nlm.nih.gov/pubmed/30620071
16. Mpirimbanyi C, Nyirimodoka A, Lin Y, Hedt-Gauthier BL, Odhiambo J, Nkurunziza T, et al. Emergency general surgery in Rwandan district hospitals: a cross-sectional study of spectrum, management, and patient outcomes. 2017;17(1):121.
17. Gajewski J, Pittalis C, Lavy C, Borgstein E, Bijlmakers L, Mwapasa G, et al. Anesthesia Capacity of District-Level Hospitals in Malawi, Tanzania, and Zambia: A Mixed-Methods Study. Anesth Analg [Internet]. 2019 Apr 1 [cited 2020 Apr 28];130(4):845–53. Available from: http://ovidsp.ovid.com/ovidweb.cgi?T=JS&PAGE=reference&D=ovftu&NEWS=N&AN=00000539-900000000-96017
18. LeBrun DG, Chackungal S, Chao TE, Knowlton LM, Linden AF, Notrica MR, et al. Prioritizing essential surgery and safe anesthesia for the Post-2015 Development Agenda: operative capacities of 78 district hospitals in 7 low- and middle-income countries. Surgery [Internet]. 2014;155(3):365–73. Available from: http://www.ncbi.nlm.nih.gov/pubmed/24439745

For peer review only - http://bmjopen.bmj.com/site/about/guidelines.xhtml

19. Grimes CE, Law RSL, Borgstein ES, Mkandawire NC, Lavy CBD. Systematic review of met and unmet need of surgical disease in rural sub-Saharan Africa. World J Surg [Internet]. 2012;36(1):8–23. Available from: http://www.ncbi.nlm.nih.gov/pubmed/22057752
20. Henry JA, Bem C, Grimes C, Borgstein E, Mkandawire N, Thomas WEGG, et al. Essential surgery: the way forward. World J Surg. 2015;39(4):822–32.
21. Sherman L, Clement P, Cherian M, Ndayimirije N, MD W-L, Noel L, et al. Implementing Liberia's Poverty Reduction Strategy: An Assessment of Emergency and Essential Surgical Care. Arch Surg [Internet]. 2011;146(1):35–9. Available from: http://ovidsp.ovid.com/ovidweb.cgi?T=JS&PAGE=reference&D=ovftl&NEWS=N&AN=00000853-201101000-00008
22. Choo S, Perry H, Hesse AAJ, Abantanga F, Sory E, Osen H, et al. Surgical training and experience of medical officers in Ghana's district hospitals. 2011;86(4). Available from: http://www.ncbi.nlm.nih.gov/pubmed/21346502
23. Fehr J, Hatz C, Soka I, Kibatala P, Urassa H, Smith T, et al. Risk Factors for Surgical Site Infection in a Tanzanian District Hospital: A Challenge for the Traditional National Nosocomial Infections Surveillance System Index. Infect Control Hosp Epidemiol [Internet]. 2006;27(12):1401–4. Available from: http://ovidsp.ovid.com/ovidweb.cgi?T=JS&PAGE=reference&D=ovfth&NEWS=N&AN=00004848-200612000-00019
24. Ngaroua, Eloundou NJ, Djibrilla Y, Asmaou O, Mbo AJ. Aspects épidémiologiques, cliniques et prise en charge de sténose urétrale chez l'adulte dans un Hôpital de District de Ngaoundéré, Cameroun. Pan Afr Med J. 2017;26.
25. Hyder AA, Wunderlich CA, Puvanachandra P, Gururaj G, Kobusingye OC. The impact of traumatic brain injuries: A global perspective. Vol. 22, NeuroRehabilitation. IOS Press; 2007. p. 341–53.
26. World Health Organization. Surgical Care at the District Hospital. 2003.
27. Van Amelsfoort JJC, Van Leeuwen PAM, Jiskoot P, Ratsma YEC. Surgery in Malawi – the training of clinical officers. Trop Doct. 2010;40(2):74–6.
28. Abdullah F, Choo S, Hesse AAJ, Abantanga F, Sory E, Osen H, et al. Assessment of surgical and obstetrical care at 10 district hospitals in Ghana using on-site interviews. J Surg Res [Internet]. 2011;171(2):461–6. Available from: http://www.ncbi.nlm.nih.gov/pubmed/20691981
29. Damien P, Nabare C, Baiden F, Kwara E, Apanga S, Etego-Amengo S. How are surgical theatres in rural Africa utilized? A review of five years of services at a district hospital in Ghana. Trop Doct [Internet]. 2011;41(2):91–5. Available from: http://www.ncbi.nlm.nih.gov/pubmed/21421886
30. Gyedu A, Stewart B, Gaskill C, Boakye G, Appiah-Denkyira E, Donkor P, et al. Improving Benchmarks for Global Surgery: Nationwide Enumeration of Operations Performed in Ghana. Ann Surg. 2018;268(2):282–8.
31. Löfgren J, Kadobera D, Forsberg BC, Mulowooza J, Wladis A, Nordin P. District-level surgery in Uganda: Indications, interventions and perioperative mortality. Surg (United States). 2015;158(1):7–16.
32. Lavy C, Tindall A, Steinlechner C, Mkandawire N, Chimangeni S. Surgery in Malawi - a national survey of activity in rural and urban hospitals. Ann R Coll Surg Engl [Internet]. 2007;89(7):722–4. Available from: http://ovidsp.ovid.com/ovidweb.cgi?T=JS&PAGE=reference&D=ovfti&NEWS=N&AN=00000639-200710000-00025
33. Nordberg E, Mwobobia I, Muniu E. Major and minor surgery output at district level in Kenya: review and issues in need of further research. Afr J Health Sci. 2002;9(1):17–

For peer review only - http://bmjopen.bmj.com/site/about/guidelines.xhtml

25.
34. Notrica MR, Evans FM, Knowlton LM, Kelly McQueen KA. Rwandan surgical and anesthesia infrastructure: A survey of district hospitals. World J Surg. 2011;35(8):1770–80.
35. Ottaway AJ, Kabongo L. Prospective Observational Study of Intraoperative Anesthetic Events in District Hospitals in Namibia. 2018;126(2):632–8. Available from: http://www.ncbi.nlm.nih.gov/pubmed/29261548
36. Petroze RT, Nzayisenga A, Rusanganwa V, Ntakiyiruta G, Calland JF. Comprehensive national analysis of emergency and essential surgical capacity in Rwanda. 2012;99(3). Available from: http://www.ncbi.nlm.nih.gov/pubmed/22237597
37. Sani R, Nameoua B, Yahaya A, Hassane I, Adamou R, Hsia RY, et al. The impact of launching surgery at the district level in Niger. World J Surg. 2009;33(10):2063–8.
38. Gajewski J, Bijlmakers L, Mwapasa G, Borgstein E, Pittalis C, Brugha R. "I think we are going to leave these cases". Obstacles to surgery in rural Malawi: a qualitative study of provider perspectives. Trop Med Int Heal [Internet]. 2018 Oct 1 [cited 2020 Apr 21];23(10):1141–7. Available from: http://ovidsp.ovid.com/ovidweb.cgi?T=JS&PAGE=reference&D=ovftt&NEWS=N&AN=00060771-201810000-00013
39. Lonnee H, Madzimbamuto F, Erlandsen O, Vassenden A, Chikumba E, Dimba R, et al. Anesthesia for Cesarean Delivery: A Cross-Sectional Survey of Provincial, District, and Mission Hospitals in Zimbabwe. Anesth Analg [Internet]. 2018;126(6):2056–64. Available from: http://ovidsp.ovid.com/ovidweb.cgi?T=JS&PAGE=reference&D=ovftt&NEWS=N&AN=00000539-201806000-00040
40. Ouédraogo CM, Ouédraogo A, Ouattara A, Lankoandé J. [Cesarean deliveries in a district hospital in Ouagadougou. Epidemiological, clinical, and prognostic study of 3381 cases]. Med Sante Trop [Internet]. 25(2):194–9. Available from: http://www.ncbi.nlm.nih.gov/pubmed/26081260
41. Ouédraogo CM, Ouattara A, Ouédraogo A, Bikienga M, Lankoandé J. Amélioration de l'offre des soins obstétricaux par la délégation des tâches en milieu Africain Exemple de l'hôpital du district sanitaire de Bogodogo à Ouagadougou, Burkina Faso. Med Sante Trop. 2015;25(3):280–4.
42. Petroze RT, Mehtsun W, Nzayisenga A, Ntakiyiruta G, Sawyer RG, Calland JF, et al. Ratio of cesarean sections to total procedures as a marker of district hospital trauma capacity. World J Surg. 2012;36(9):2074–9.
43. Rutgers RAK, Van Eygen L. Mortality related to caesarean section in rural Matebeleland North Province, Zimbabwe. Cent Afr J Med [Internet]. 54(5):24–7. Available from: http://www.ncbi.nlm.nih.gov/pubmed/21650076
44. Fenton PM, Whitty CJM, Reynolds F. Caesarean section in Malawi: prospective study of early maternal and perinatal mortality. BMJ. 2003;327(7415):587.
45. Hall E, Sevugu J, Danso K, Adomako J, Peltzman T, Anderson FJ. A retrospective analysis of the impact of an obstetrician on delivery and care outcomes at four district hospitals in Ghana. Int J Gynaecol Obstet [Internet]. 2015;131(1):100–1. Available from: http://www.ncbi.nlm.nih.gov/pubmed/26143230
46. Luo EM, Opare-Ado HS, Adomako J, Danso KA, Peltzman T, Anderson FWJJ. Completing the Maternal Care Team: OB/GYN Expertise at Rural District Hospitals in Ghana, a Qualitative Study. 2018;22(7):1085–91. Available from: http://www.ncbi.nlm.nih.gov/pubmed/29520728
47. McCord C. Facilities for Surgery Exist in Ghana, but Skills Are Lacking: Comment on "Assessing the Surgical and Obstetrics-Gynecology Workload of Medical Officers".

For peer review only - http://bmjopen.bmj.com/site/about/guidelines.xhtml

Arch Surg [Internet]. 2012;147(6):548–9. Available from: http://ovidsp.ovid.com/ovidweb.cgi?T=JS&PAGE=reference&D=ovftm&NEWS=N&AN=00000853-201206000-00013
48. Mehtsun WT, Weatherspoon K, McElrath L, Chima A, Torsu VEK, Obeng ENB, et al. Assessing the surgical and obstetrics-gynecology workload of medical officers: findings from 10 district hospitals in Ghana. Arch Surg [Internet]. 2012;147(6):542–8. Available from: http://www.ncbi.nlm.nih.gov/pubmed/22786541
49. Smiley K, Ofori L, Spangler C, Acquaah-Arhin R, Deh D, Enos J, et al. Safety Culture and Perioperative Quality at the Volta River Authority Hospital in Akosombo, Ghana. 2019;43(1):16–23. Available from: http://www.ncbi.nlm.nih.gov/pubmed/30109388
50. Compaoré GD, Sombié I, Ganaba R, Hounton S, Meda N, Brouwere V De, et al. Readiness of district and regional hospitals in Burkina Faso to provide caesarean section and blood transfusion services: a cross-sectional study. BMC Pregnancy Childbirth. 2014;14:158.
51. Gajewski J, Borgstein E, Bijlmakers L, Mwapasa G, Aljohani Z, Pittalis C, et al. Evaluation of a surgical training programme for clinical officers in Malawi. BJS [Internet]. 2019;106(2):e156–65. Available from: https://onlinelibrary.wiley.com/doi/abs/10.1002/bjs.11065
52. Gajewski J, Conroy R, Bijlmakers L, Mwapasa G, McCauley T, Borgstein E, et al. Quality of Surgery in Malawi: Comparison of Patient-Reported Outcomes After Hernia Surgery Between District and Central Hospitals. World J Surg. 2018;42(6):1610–6.
53. Henry J, Frenkel E, Borgstein E, Mkandawire N, Goddia C. Surgical and anaesthetic capacity of hospitals in Malawi: key insights. Heal Policy Plan [Internet]. 2015;30(8):985–94. Available from: http://ovidsp.ovid.com/ovidweb.cgi?T=JS&PAGE=reference&D=ovftq&NEWS=N&AN=00003845-201510000-00005
54. De Brouwere V, Dieng T, Diadhiou M, Witter S, Denerville E. Task shifting for emergency obstetric surgery in district hospitals in Senegal. Reprod Health Matters. 2009;17(33):32–44.
55. McCord C, Mbaruku G, Pereira C, Nzabuhakwa C, Bergstrom S. The quality of emergency obstetrical surgery by assistant medical officers in Tanzanian district hospitals. Health Aff (Millwood) [Internet]. 28(5):w876-85. Available from: http://www.ncbi.nlm.nih.gov/pubmed/19661113
56. Dresser, Caleb; Periyanayagam, Usha; Dreifuss, Brad; Wangoda, Robert; Luyimbaazi, Julius; Bisanzo MGECCIGECC. Management and Outcomes of Acute Surgical Patients at a District Hospital in Uganda with Non-physician Emergency Clinicians. World J Surg [Internet]. 2017 [cited 2020 Apr 28]; Available from: https://rayyan.qcri.org/fulltexts/320248
57. Cheelo M, Brugha R, Bijlmakers L, Kachimba J, McCauley T, Gajewski J. Surgical Capacity at District Hospitals in Zambia: From 2012 to 2016. World J Surg. 2018 Nov 1;42(11):3508–13.
58. Choo S, Papandria D, Goldstein SD, Perry H, Hesse AAJ, Abatanga F, et al. Quality improvement activities for surgical services at district hospitals in developing countries and perceived barriers to quality improvement: findings from Ghana and the scientific literature. World J Surg [Internet]. 2013;37(11):2512–9. Available from: http://www.ncbi.nlm.nih.gov/pubmed/23897444
59. Koigi-Kamau R, Kabare LW, Wanyoike-Gichuhi J. Incidence of wound infection after Caesarean delivery in a district hospital in Central Kenya. East Afr Med J. 2005;82(7):357–61.

For peer review only - http://bmjopen.bmj.com/site/about/guidelines.xhtml

60. Harfouche M, Hosseinipour M, Kaliti S, Wilkinson J. Quality indicators and outcomes of emergency caesarean deliveries at a district-level maternity hospital. Afr J Reprod Health. 2015;19(3):61–7.
61. Stewart BT, Quansah R, Gyedu A, Ankomah J, Donkor P, Mock C. Strategic Assessment of Trauma Care Capacity in Ghana. World J Surg. 2015;39(10):2428–40.
62. van den Akker T, Mwagomba B, Irlam J, van Roosmalen J. Using audits to reduce the incidence of uterine rupture in a Malawian district hospital. Int J Gynaecol Obstet [Internet]. 2009;107(3):289–94. Available from: http://www.ncbi.nlm.nih.gov/pubmed/19846089
63. Kenfack B, Noubom M, Bongoe A, Tsatedem FA, Ngono M, Tsague GN, et al. [Ectopic pregnancy in a semi-rural area in Africa: Epidemiological, clinical and therapeutic aspects about a series of 74 cases treated at the District Hospital of Sangmelima in southern Cameroon]. Pan Afr Med J. 2012;13:71.
64. Fehr J, Hatz C, Soka I, Kibatala P, Urassa H, Battegay M, et al. Antimicrobial prophylaxis to prevent surgical site infections in a rural sub-Saharan hospital. Clin Microbiol Infect [Internet]. 2006;12(12):1224–7. Available from: http://ovidsp.ovid.com/ovidweb.cgi?T=JS&PAGE=reference&D=ovfth&NEWS=N&AN=00125357-200612000-00012
65. Meara, J. G., Leather, A. J., Hagander, L., Alkire, B. C., Alonso, N., Ameh EA. Global Surgery 2030: evidence and solutions for achieving health, welfare, and economic development. Lancet. 2015;386(9993):569–624.

For peer review only

For peer review only - http://bmjopen.bmj.com/site/about/guidelines.xhtml

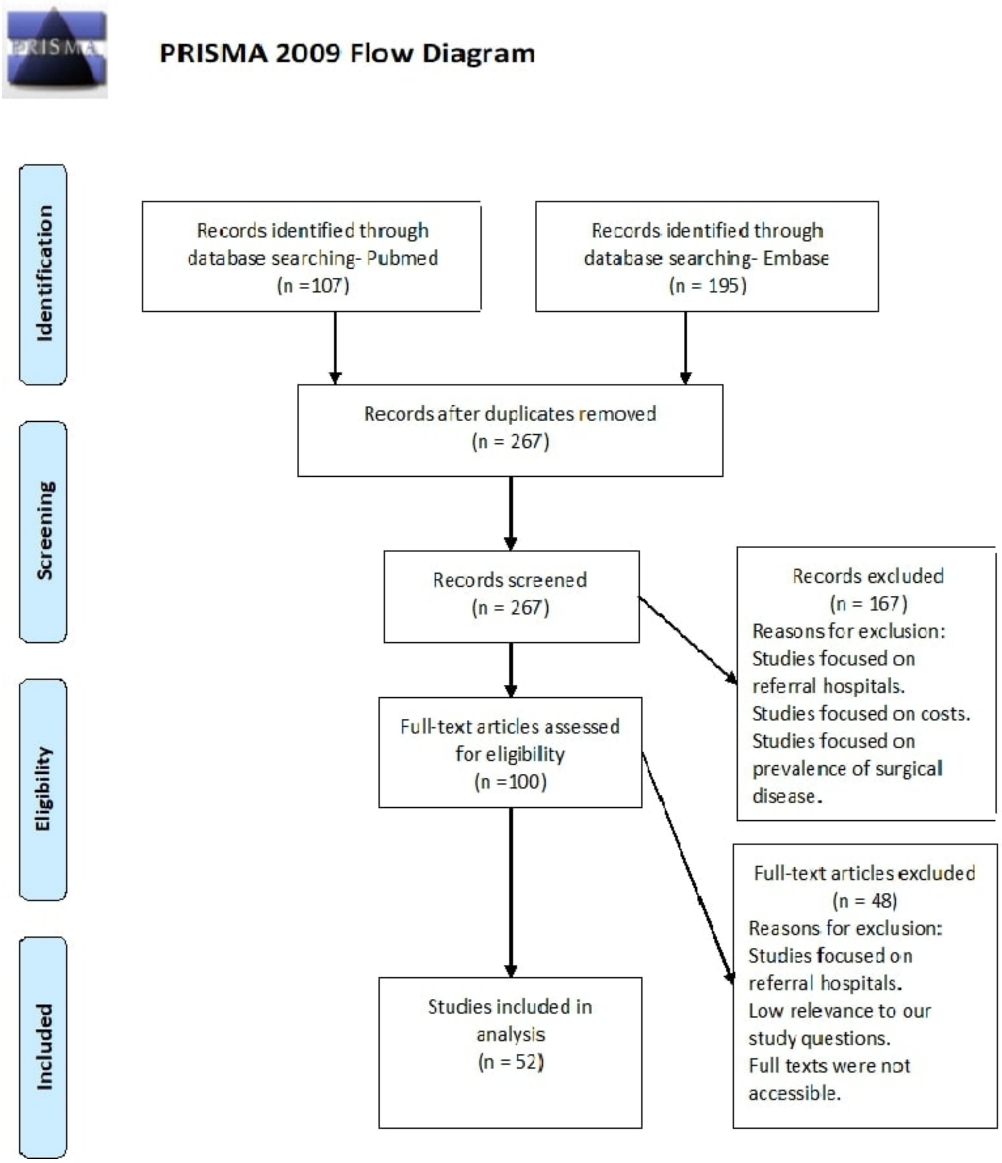

Figure 1: PRISMA diagram illustrating the selection process

269x312mm (72 x 72 DPI)

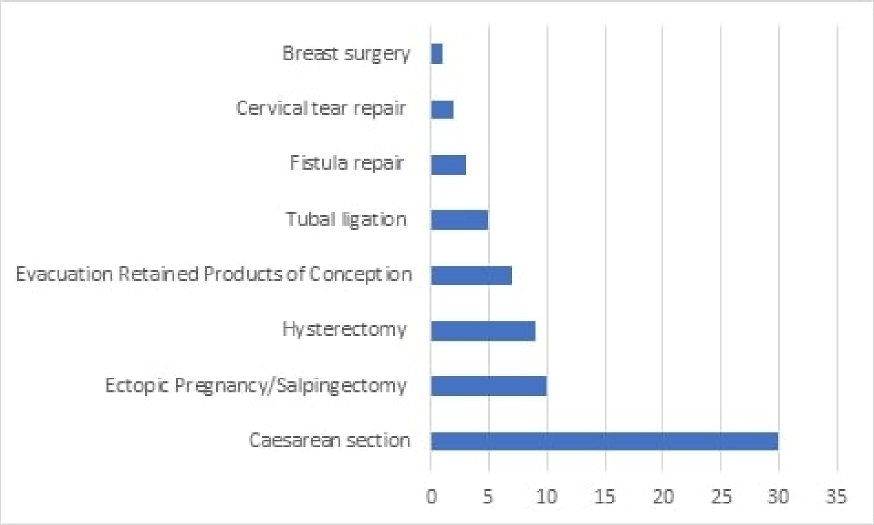

Figure 2: Number of articles reporting obstetrics and gynaecology operations

193x106mm (72 x 72 DPI)

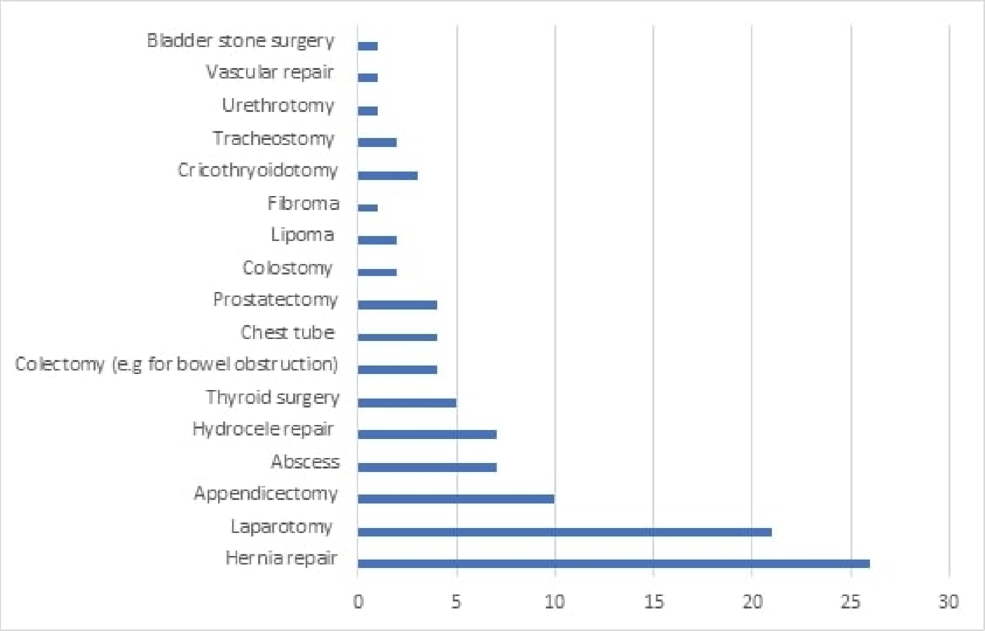

Figure 3: Number of articles reporting general surgery operations

201x133mm (72 x 72 DPI)

Systematic Search:

Dates: From November 12th to November 18th, 2019.

Databases: Pubmed and Embase

Languages: English, French, Portuguese

Time: from January 1st, 2000 onwards

Key search terms used:

"surgery"[Subheading] OR "surgical procedures, operative"[MeSH Terms] OR "Specialties, Surgical"[Mesh] AND "Hospitals, District"[Mesh] AND "Africa South of the Sahara"[Mesh] for Pubmed

“surgery" AND "sub saharan africa" AND "district hospital" for Embase.

Supplementary Table 1: Summary of studies selected

First Author	Year of Publication	Study setting	Study Design	Objective	Reference
Abdullah	2011	Ghana	Mixed methods	Assess surgical and obstetrical capacity	(28)
Cheelo	2018	Zambia	Cross-sectional survey	Assess surgical capacity	(57)
Choo	2013	Ghana	Cross-sectional survey	Appraise quality improvement activities	(58)
Choo	2011	Ghana	Qualitative methods	Explore the training of medical officers providing surgery	(22)
Compaoré	2014	Burkina Faso	Cross-sectional survey	Assess the readiness to provide caesarean sections	(50)
Damien	2011	Ghana	Review of hospital records	Evaluate surgical volume	(29)
De Brouwere	2009	Senegal	Mixed methods	Evaluate the task shifting policy	(54)
Dossche	2014	Burkina Faso	Case series	Analyse technical	(13)

For peer review only

				problems and post-operative complications	
Dresser	2017	Uganda	Review of hospital records	Evaluate the management of surgical patients by non-physician emergency clinicians	(56)
Fehr	2006	Tanzania	Prospective cohort study	Identify risk factors for surgical site infection	(23)
Fehr	2006	Tanzania	Prospective cohort study	Evaluate peri-operative antimicrobial prohylaxis	(64)
Fenton	2003	Malawi	Prospective cohort study	Identify risk factors associated with maternal death after caesarean section	(44)
Gajewski	2017	Malawi	Baseline and follow up patients' surveys	Compare surgical outcomes between central and district hospitals	(52)
Gajewski	2019	Malawi, Tanzania, Zambia	Mixed-methods	Investigate of anaesthesia capacity	(17)
Gajewski	2018	Malawi	Qualitative methods	Explore providers perspectives on obstacles to surgery	(38)
Gajewski	2019	Malawi	Randomised Control Trial	Evaluate surgical training programme for clinical officers	(51)
Galukande	2010	Uganda, Tanzania, Mozambique	Review of hospital records	Assess the scope of surgery provided	(4)

For peer review only

Grimes	2012	Sub-Saharan Africa	Systematic Review	Identify the met and unmet needs of surgical disease	(19)
Gyedu	2018	Ghana	Cross-sectional survey	Evaluate the operation rate in Ghana	(30)
Hall	2015	Ghana	Review of hospital records	Evaluate the impact of a newly placed obstetrician on health outcomes	(45)
Harfouche	2015	Malawi	Prospective cohort study	Assess the quality of emergency caesarean sections	(60)
Henry	2015	Sub-Saharan Africa	Consensus meeting+ interviews	Identify a package of essential surgical operations	(20)
Henry	2015	Malawi	Cross-sectional survey	Assess surgical capacity	(53)
Kenfack	2012	Cameroon	Case series	Describe the management of ectopic pregnancy	(63)
Koigi-Kamau	2005	Kenya	Prospective cohort study	Describe the incidence of post-caesarean wound infection	(59)
Lavy	2007	Malawi	Review of hospital records	Estimate surgical volume	(32)
LeBrun	2014	Ethiopia, Liberia, Rwanda, Uganda	Cross-sectional survey	Assess surgical capacity	(18)
Lofgren	2015	Uganda	Cross-sectional survey	Investigate indications, interventions and peri-operative mortality	(31)

For peer review only

Lonnée	2018	Zimbabwe	Cross-sectional survey	Describe anaesthesia care for caesarean sections	(39)
Luo	2018	Ghana	Qualitative	Explore the impact of a newly placed obstetrician in a district hospital	(46)
McCord	2012	Ghana	Commentary	Describe surgical care in district hospitals in Ghana	(47)
McCord	2009	Tanzania	Review of hospital records	Evaluate the quality of emergency obstetric surgery done by assistant medical officers	(55)
Mehtsun	2012	Ghana	Observational study	Assess surgical workload of medical officers	(48)
Mpirimbanyi	2017	Rwanda	Cross-sectional survey	Describe the spectrum, management and patient outcomes of emergency surgery	(16)
Muhirwa	2016	Rwanda	Cohort study	Describe the presentation, management and outcomes of surgical patients	(14)
Ngaroua	2017	Cameroon	Case series	Describe the management of urethral stenosis	(24)
Nkurunziza	2019	Rwanda	Cross-sectional survey	Estimate the prevalence of surgical site infection after	(15)

For peer review only

				caesarean section	
Nordberg	2002	Kenya	Cross-sectional survey	Estimate of the surgical volume	(33)
Notrica	2011	Rwanda	Cross-sectional survey	Assess surgical and anaesthesia infrastructure	(34)
Ottaway	2018	Namibia	Prospective Observational Study	Determine the prevalence of anaesthetic adverse events	(35)
Ouédraogo	2015	Burkina Faso	Prospective Observational Study	Describe task shifting in obstetric care	(41)
Ouédraogo	2015	Burkina Faso	Case series	Describe prognosis of patients undergoing caesarean sections	(40)
Petroze	2012	Rwanda	Cross-sectional survey	Evaluate the Ratio of caesarean sections to total procedures as a marker of trauma capacity	(42)
Petroze	2012	Rwanda	Mixed methods	Assess emergency and essential surgical capacity	(36)
Ramos	2013	Ethiopia	Case series	Describe thyroid surgery cases	(12)
Rutgers	2008	Zimbabwe	Cross-sectional survey	Describe the mortality associated with caesarean sections	(43)
Sani	2009	Niger	Cross-sectional survey	Assess surgical care provided by	(37)

For peer review only

				general practitioners	
Sherman	2011	Liberia	Cross-sectional survey	Assess emergency and essential surgical care	(21)
Smiley	2019	Ghana	Surveys + process mapping	Evaluate patients and staff perception of quality of care + Analyse peri-operative process	(49)
Stewart	2015	Ghana	Cross-sectional survey	Assess trauma capacity	(61)
Van Amelsfoort	2010	Malawi	Mixed methods	Describe the training and experiences of clinical officers	(27)
Van den Akker	2009	Malawi	Mixed methods	Evaluate the impact of audits in reducing the incidence of uterine ruptures	(62)

For peer review only

Preferred Reporting Items for Systematic reviews and Meta-Analyses extension for Scoping Reviews (PRISMA-ScR) Checklist

SECTION	ITEM	PRISMA-ScR CHECKLIST ITEM	REPORTED ON PAGE #
**TITLE**			
Title	1	Identify the report as a scoping review.	Title
**ABSTRACT**			
Structured summary	2	Provide a structured summary that includes (as applicable): background, objectives, eligibility criteria, sources of evidence, charting methods, results, and conclusions that relate to the review questions and objectives.	Page 1
**INTRODUCTION**			
Rationale	3	Describe the rationale for the review in the context of what is already known. Explain why the review questions/objectives lend themselves to a scoping review approach.	Page 2
Objectives	4	Provide an explicit statement of the questions and objectives being addressed with reference to their key elements (e.g., population or participants, concepts, and context) or other relevant key elements used to conceptualize the review questions and/or objectives.	Page 2
**METHODS**			
Protocol and registration	5	Indicate whether a review protocol exists; state if and where it can be accessed (e.g., a Web address); and if available, provide registration information, including the registration number.	Prospero didn't accept to register a scoping review protocol
Eligibility criteria	6	Specify characteristics of the sources of evidence used as eligibility criteria (e.g., years considered, language, and publication status), and provide a rationale.	Page 3
Information sources*	7	Describe all information sources in the search (e.g., databases with dates of coverage and contact with authors to identify additional sources), as well as the date the most recent search was executed.	Page 3
Search	8	Present the full electronic search strategy for at least 1 database, including any limits used, such that it could be repeated.	Page 3 +supplementary material
Selection of sources of evidence†	9	State the process for selecting sources of evidence (i.e., screening and eligibility) included in the scoping review.	Page 3
Data charting process‡	10	Describe the methods of charting data from the included sources of evidence (e.g., calibrated forms or forms that have been tested by the team before their use, and whether data charting was done independently or in duplicate) and any processes for obtaining and confirming data from investigators.	Page 4
Data items	11	List and define all variables for which data were sought and any assumptions and simplifications made.	NA
Critical appraisal	12	If done, provide a rationale for conducting a critical	NA

St. Michael's
Inspired Care.
Inspiring Science.

For peer review only - http://bmjopen.bmj.com/site/about/guidelines.xhtml

SECTION	ITEM	PRISMA-ScR CHECKLIST ITEM	REPORTED ON PAGE #
of individual sources of evidence§		appraisal of included sources of evidence; describe the methods used and how this information was used in any data synthesis (if appropriate).	
Synthesis of results	13	Describe the methods of handling and summarizing the data that were charted.	Page 4
**RESULTS**			
Selection of sources of evidence	14	Give numbers of sources of evidence screened, assessed for eligibility, and included in the review, with reasons for exclusions at each stage, ideally using a flow diagram.	Page 5
Characteristics of sources of evidence	15	For each source of evidence, present characteristics for which data were charted and provide the citations.	Pages 5-12
Critical appraisal within sources of evidence	16	If done, present data on critical appraisal of included sources of evidence (see item 12).	NA
Results of individual sources of evidence	17	For each included source of evidence, present the relevant data that were charted that relate to the review questions and objectives.	Pages 5-12
Synthesis of results	18	Summarize and/or present the charting results as they relate to the review questions and objectives.	Pages 5-12
**DISCUSSION**			
Summary of evidence	19	Summarize the main results (including an overview of concepts, themes, and types of evidence available), link to the review questions and objectives, and consider the relevance to key groups.	Results and Discussion were presented together
Limitations	20	Discuss the limitations of the scoping review process.	Page 12
Conclusions	21	Provide a general interpretation of the results with respect to the review questions and objectives, as well as potential implications and/or next steps.	Page 12
**FUNDING**			
Funding	22	Describe sources of funding for the included sources of evidence, as well as sources of funding for the scoping review. Describe the role of the funders of the scoping review.	Page 13

JBI = Joanna Briggs Institute; PRISMA-ScR = Preferred Reporting Items for Systematic reviews and Meta-Analyses extension for Scoping Reviews.

* Where *sources of evidence* (see second footnote) are compiled from, such as bibliographic databases, social media platforms, and Web sites.

† A more inclusive/heterogeneous term used to account for the different types of evidence or data sources (e.g., quantitative and/or qualitative research, expert opinion, and policy documents) that may be eligible in a scoping review as opposed to only studies. This is not to be confused with *information sources* (see first footnote).

‡ The frameworks by Arksey and O'Malley (6) and Levac and colleagues (7) and the JBI guidance (4, 5) refer to the process of data extraction in a scoping review as data charting.

§ The process of systematically examining research evidence to assess its validity, results, and relevance before using it to inform a decision. This term is used for items 12 and 19 instead of "risk of bias" (which is more applicable to systematic reviews of interventions) to include and acknowledge the various sources of evidence that may be used in a scoping review (e.g., quantitative and/or qualitative research, expert opinion, and policy document).

For peer review only - http://bmjopen.bmj.com/site/about/guidelines.xhtml

From: Tricco AC, Lillie E, Zarin W, O'Brien KK, Colquhoun H, Levac D, et al. PRISMA Extension for Scoping Reviews (PRISMAScR): Checklist and Explanation. Ann Intern Med. 2018;169:467–473. doi: 10.7326/M18-0850.

For peer review only

BMJ Open

Surgical care in District Hospitals in sub-Saharan Africa: a Scoping Review

Journal:	*BMJ Open*
Manuscript ID	bmjopen-2020-042862.R1
Article Type:	Original research
Date Submitted by the Author:	19-Oct-2020
Complete List of Authors:	Bentounsi, Zineb; University of Oxford,<br>Sheik-Ali, Sharaf; University of Oxford<br>Le, Grace; Nuffield Department of Orthopaedics, Rheumatology and Musculoskeletal Sciences (NDORMS),<br>Lavy, Chris; Nuffield Department of Orthopaedics, Rheumatology and Musculoskeletal Sciences (NDORMS), ,
**Primary Subject Heading**:	Surgery
Secondary Subject Heading:	Anaesthesia
Keywords:	SURGERY, HEALTH SERVICES ADMINISTRATION & MANAGEMENT, ANAESTHETICS

SCHOLARONE™
Manuscripts

BMJ

I, the Submitting Author has the right to grant and does grant on behalf of all authors of the Work (as defined in the below author licence), an exclusive licence and/or a non-exclusive licence for contributions from authors who are: i) UK Crown employees; ii) where BMJ has agreed a CC-BY licence shall apply, and/or iii) in accordance with the terms applicable for US Federal Government officers or employees acting as part of their official duties; on a worldwide, perpetual, irrevocable, royalty-free basis to BMJ Publishing Group Ltd ("BMJ") its licensees and where the relevant Journal is co-owned by BMJ to the co-owners of the Journal, to publish the Work in this journal and any other BMJ products and to exploit all rights, as set out in our licence.

The Submitting Author accepts and understands that any supply made under these terms is made by BMJ to the Submitting Author unless you are acting as an employee on behalf of your employer or a postgraduate student of an affiliated institution which is paying any applicable article publishing charge ("APC") for Open Access articles. Where the Submitting Author wishes to make the Work available on an Open Access basis (and intends to pay the relevant APC), the terms of reuse of such Open Access shall be governed by a Creative Commons licence – details of these licences and which Creative Commons licence will apply to this Work are set out in our licence referred to above.

Other than as permitted in any relevant BMJ Author's Self Archiving Policies, I confirm this Work has not been accepted for publication elsewhere, is not being considered for publication elsewhere and does not duplicate material already published. I confirm all authors consent to publication of this Work and authorise the granting of this licence.

For peer review only

Surgical care in District Hospitals in sub-Saharan Africa: a Scoping Review

Authors:

Zineb Bentounsi, corresponding author, Botnar Research Centre, NDORMS, University of Oxford, Old Road, OX3 7LD, Oxford, UK. 07727064357, zineb.bentounsi@ndorms.ox.ac.uk

Sharaf Sheik Ali, sharaf.sheik-ali@medsci.ox.ac.uk Botnar Research Centre, NDORMS, University of Oxford, UK.

Grace Le, grace.le@ndorms.ox.ac.uk Botnar Research Centre, NDORMS, University of Oxford, UK.

Chris Lavy, chris.lavy@ndorms.ox.ac.uk Botnar Research Centre, NDORMS, University of Oxford, UK.

Keywords:

Global Surgery, Surgery, District Hospitals, Africa

Word count: 2877

For peer review only

Abstract:

Objective: To summarize the body of literature existing on the provision of surgical and anaesthesia care in district hospitals in sub-Saharan Africa.

Design: Scoping Review

Setting: District hospitals in sub-Saharan Africa

Data sources: PubMed and Ovid EMBASE from January 2000 to December 2019

Study selection: Studies were included if they contained information about types of surgical procedures performed, number of operations per year, types of anaesthesia delivered, cadres of surgical/anaesthesia providers and/or patients' outcomes.

Results: The 52 articles included in analysis provided information about 16 countries. District hospitals were a group of diverse institutions ranging from 21 to 371 beds. The 3 most frequently reported procedures were caesarean section, laparotomy and hernia repair but a wide range of orthopaedics, plastic surgery and neurosurgery procedures were also mentioned. The number of operations performed per year per district hospital ranged from 239 to 5233. The most mentioned anaesthesia providers were non-physician clinicians trained in anaesthesia. They deliver mainly general and spinal anaesthesia. Depending on countries, articles referred to different surgical care providers: specialist surgeons, medical officers and non-physician clinicians. 15 articles reported peri-operative complications among which surgical site infection was the most frequent. 15 articles reported perioperative deaths of which the leading causes were sepsis, haemorrhage and anaesthesia complications.

Conclusion: District hospitals play a significant role in sub-Saharan Africa, providing both emergency and elective surgery. Most procedures are done under general or spinal anaesthesia, often administered by non-physician clinicians. Depending on countries, surgical care may be provided by medical officers, specialist surgeons and/or non-physician clinicians. Research on safety, quality and volume of surgical and anaesthesia care in this setting is scarce and more attention to these questions is required.

For peer review only

Article Summary:

Strengths and limitations:

- This is the first review to systematically assess the literature for the scope, volume and quality of surgery in district hospitals in sub-Saharan Africa.
- Article selection and data extraction were conducted independently by two authors
- Our search was conducted in English, French and Portuguese.
- This study is prone to publication bias as it is based on information present in published literature.
- There was a lot of heterogeneity in how articles reported surgical volume and quality.

For peer review only - http://bmjopen.bmj.com/site/about/guidelines.xhtml

Introduction:

The burden of surgical disease in Sub-Saharan Africa (SSA) is estimated at 38 disability adjusted life years (DALYs) per 1,000 population. District hospitals bear a large burden of this surgical care delivery(1). According to The World Health Organization (WHO), district hospitals are defined as the first level of healthcare facilities that provide in-patient surgery and anaesthesia(2). These hospitals which mainly serve rural populations, face challenges in terms of workforce, infrastructure and other essential resources. In 2010, it has been estimated that there was only 1 surgeon for 25 million people in rural SSA(3) and although training programs in the region are increasing the number of surgeons, the main burden in several countries remains on non-physician clinicians to manage surgical cases(4).

District hospitals (DHs) offer a unique opportunity to improve access to surgical care in SSA as they are the first point of access to surgical care for patients and relieve referral hospitals from undue burden, allowing them to focus on complicated cases. Delivering timely surgical care in DHs has been shown to be highly cost effective(5) which should provide encouragement for governments and global health programs to invest in them. The Lancet Commission on Global Surgery (65) reinforces this emphasis on district hospitals and echoes other authors' calls to consider DHs as a central component in strengthening surgical care in SSA(6)(7).

The authors developed an interest in District hospitals in sub-Saharan Africa through research and training partnerships with colleagues in the region. We considered a systematic review of district hospital surgery, however in view of the limited number of existing studies in this field and the wide range of methodology used, we chose to change the review methodology and undertake a scoping review of surgical care delivery in district hospitals in SSA, aiming to provide a general overview of the reported current surgical capacity and delivery, advance current knowledge and suggest targets for further development and research within the region of SSA.

Methods:

This scoping review follows the five step methodology designed by Arksey and O'Malley(8) that offers a rigorous framework for reporting findings. We have also considered the recommendations of Levac, Colquhoun and O'Brien(9) that supplement this framework.

1. Identifying the research question

To guide our search of the literature, we defined the following questions:

Primary outcome:
In district hospitals in sub Saharan Africa, what types of surgical procedures are provided?

Secondary outcomes:
In district hospitals in sub-Saharan Africa:

- How many surgical procedures are performed per year?
- Are there any measures of quality, safety or outcomes of surgery?
- Who provides anaesthesia and/or surgery?
- What type of anaesthesia is delivered?

For peer review only - http://bmjopen.bmj.com/site/about/guidelines.xhtml

2. Identifying relevant studies

PubMed and Ovid EMBASE were searched on 18/12/19 to retrieve literature about surgery in district hospitals in sub-Saharan Africa. The search strategy combined the exploded thesaurus terms “surgery”, “surgical procedures, operative” and “specialties, surgical” with the exploded thesaurus terms “hospitals, district” and “Africa south of the Sahara”. References were searched in English, French and Portuguese. We limited the search to references published after January 1st, 2000 this giving us a 20 year study period. Google Scholar and the Cochrane Library were also searched with the same search terms, but they did not contribute any relevant new references.

3. Study selection

The most important inclusion criteria was the setting: a district hospital in sub-Saharan Africa. Articles were included when they provided information about either type of surgical procedures performed, number of operations per year, types of anaesthesia delivered, cadres of surgical/anaesthesia providers and/or patients outcomes. They were excluded if the setting was unclear or was not a district hospital, and if they were strictly about costs or burden of disease. Articles for which the full text could not be retrieved were excluded. All surgical procedures were included except dental and ophthalmic procedures, as these are often done in outpatient departments

The screening process was done independently by Z.B and G.L using the web application Rayyan(10). Conflict was resolved by dialogue between Z.B, G.L and C.L. The process of selection followed the Preferred Reporting of Items for Systematic Reviews and Meta-Analyses (PRISMA) Statement.

4. Charting the data

The data charting form was developed by Z.B after consultation with all other authors. The form was piloted and then updated iteratively during the extraction process (see supplementary file). The form contained 20 fields of data entry covering information about articles (e.g. name of first author, year of publication, research method) and information about district hospitals, surgical and anaesthesia providers, surgical procedures as well as other relevant information. Data was extracted independently by Z.B and S.S.

5. Collating, summarizing and reporting the results

A descriptive numerical summary of surgical volume, types of surgical procedures and types of anaesthesia was produced by S.S using Excel. Graphs were also produced in Excel. A thematic analysis was performed by Z.B in NVivo(11) with surgical providers, quality of care and surgical outcomes as themes. Results were reported to answer the primary and secondary outcomes of the review. Implications for research and policy were discussed and reported by all authors.

For peer review only - http://bmjopen.bmj.com/site/about/guidelines.xhtml

Results:

In this section, results and discussion are simultaneously presented. Characteristics of the included articles and an analysis of the district hospitals are first explored. Thereafter, we present our findings on the scope and volume of surgery, types of anaesthesia, surgical and anaesthesia providers, and finally safety and quality of care.

1. Study Selection

We identified 302 articles and after removing duplicates 267 articles remained. The titles and abstracts were screened, and, using our criteria noted above, 167 articles were excluded. Then, full texts of the remaining 100 articles were assessed and 48 were excluded. Therefore, 52 articles were included for analysis. A PRISMA diagram is presented in Figure 1.

Figure 1: PRISMA diagram illustrating the selection process

2. Study Characteristics

Thirty three per cent of articles were cross-sectional surveys, 17% were prospective studies and 11% were based on retrospective review of hospital records (see Table 1). Most articles (93%) described the surgical care provided by staff based at the hospital, however, four articles reported external support from Non-Governmental Organisations (NGO) or visiting surgical teams (12–16)

Table 1: Summary of studies selected

First Author	Year of Publication	Study setting	Study Design	Objective	Reference
Abdullah	2011	Ghana	Mixed methods	Assess surgical and obstetrical capacity	(28)
Cheelo	2018	Zambia	Cross-sectional survey	Assess surgical capacity	(57)
Choo	2013	Ghana	Cross-sectional survey	Appraise quality improvement activities	(58)
Choo	2011	Ghana	Qualitative methods	Explore the training of medical officers providing surgery	(22)
Compaoré	2014	Burkina Faso	Cross-sectional survey	Assess the readiness to provide caesarean sections	(50)

For peer review only - http://bmjopen.bmj.com/site/about/guidelines.xhtml

Damien	2011	Ghana	Review of hospital records	Evaluate surgical volume	(29)
De Brouwere	2009	Senegal	Mixed methods	Evaluate the task shifting policy	(54)
Dossche	2014	Burkina Faso	Case series	Analyse technical problems and post-operative complications	(13)
Dresser	2017	Uganda	Review of hospital records	Evaluate the management of surgical patients by non-physician emergency clinicians	(56)
Fehr	2006	Tanzania	Prospective cohort study	Identify risk factors for surgical site infection	(23)
Fehr	2006	Tanzania	Prospective cohort study	Evaluate peri-operative antimicrobial prohylaxis	(64)
Fenton	2003	Malawi	Prospective cohort study	Identify risk factors associated with maternal death after caesarean section	(44)
Gajewski	2017	Malawi	Baseline and follow up patients' surveys	Compare surgical outcomes between central and district hospitals	(52)
Gajewski	2019	Malawi, Tanzania, Zambia	Mixed-methods	Investigate of anaesthesia capacity	(17)
Gajewski	2018	Malawi	Qualitative methods	Explore providers perspectives on obstacles to surgery	(38)

For peer review only
For peer review only - http://bmjopen.bmj.com/site/about/guidelines.xhtml

Gajewski	2019	Malawi	Randomised Control Trial	Evaluate surgical training programme for clinical officers	(51)
Galukande	2010	Uganda, Tanzania, Mozambique	Review of hospital records	Assess the scope of surgery provided	(4)
Grimes	2012	Sub-Saharan Africa	Systematic Review	Identify the met and unmet needs of surgical disease	(19)
Gyedu	2018	Ghana	Cross-sectional survey	Evaluate the operation rate in Ghana	(30)
Hall	2015	Ghana	Review of hospital records	Evaluate the impact of a newly placed obstetrician on health outcomes	(45)
Harfouche	2015	Malawi	Prospective cohort study	Assess the quality of emergency caesarean sections	(60)
Henry	2015	Sub-Saharan Africa	Consensus meeting+ interviews	Identify a package of essential surgical operations	(20)
Henry	2015	Malawi	Cross-sectional survey	Assess surgical capacity	(53)
Kenfack	2012	Cameroon	Case series	Describe the management of ectopic pregnancy	(63)
Koigi-Kamau	2005	Kenya	Prospective cohort study	Describe the incidence of post-caesarean wound infection	(59)
Lavy	2007	Malawi	Review of hospital records	Estimate surgical volume	(32)

For peer review only - http://bmjopen.bmj.com/site/about/guidelines.xhtml

LeBrun	2014	Ethiopia, Liberia, Rwanda, Uganda	Cross-sectional survey	Assess surgical capacity	(18)
Lofgren	2015	Uganda	Cross-sectional survey	Investigate indications, interventions and peri-operative mortality	(31)
Lonnée	2018	Zimbabwe	Cross-sectional survey	Describe anaesthesia care for caesarean sections	(39)
Luo	2018	Ghana	Qualitative methods	Explore the impact of a newly placed obstetrician in a district hospital	(46)
McCord	2012	Ghana	Commentary	Describe surgical care in district hospitals in Ghana	(47)
McCord	2009	Tanzania	Review of hospital records	Evaluate the quality of emergency obstetric surgery done by assistant medical officers	(55)
Mehtsun	2012	Ghana	Observational study	Assess surgical workload of medical officers	(48)
Mpirimbanyi	2017	Rwanda	Cross-sectional survey	Describe the spectrum, management and patient outcomes of emergency surgery	(16)
Muhirwa	2016	Rwanda	Cohort study	Describe the presentation, management and outcomes	(14)

For peer review only - http://bmjopen.bmj.com/site/about/guidelines.xhtml

				of surgical patients	
Ngaroua	2017	Cameroon	Case series	Describe the management of urethral stenosis	(24)
Nkurunziza	2019	Rwanda	Cross-sectional survey	Estimate the prevalence of surgical site infection after caesarean section	(15)
Nordberg	2002	Kenya	Cross-sectional survey	Estimate of the surgical volume	(33)
Notrica	2011	Rwanda	Cross-sectional survey	Assess surgical and anaesthesia infrastructure	(34)
Ottaway	2018	Namibia	Prospective Observational Study	Determine the prevalence of anaesthetic adverse events	(35)
Ouédraogo	2015	Burkina Faso	Prospective Observational Study	Describe task shifting in obstetric care	(41)
Ouédraogo	2015	Burkina Faso	Case series	Describe prognosis of patients undergoing caesarean sections	(40)
Petroze	2012	Rwanda	Cross-sectional survey	Evaluate the Ratio of caesarean sections to total procedures as a marker of trauma capacity	(42)
Petroze	2012	Rwanda	Mixed methods	Assess emergency and essential surgical capacity	(36)

For peer review only - http://bmjopen.bmj.com/site/about/guidelines.xhtml

Ramos	2013	Ethiopia	Case series	Describe thyroid surgery cases	(12)
Rutgers	2008	Zimbabwe	Cross-sectional survey	Describe the mortality associated with caesarean sections	(43)
Sani	2009	Niger	Cross-sectional survey	Assess surgical care provided by general practitioners	(37)
Sherman	2011	Liberia	Cross-sectional survey	Assess emergency and essential surgical care	(21)
Smiley	2019	Ghana	Surveys + process mapping	Evaluate patients and staff perception of quality of care + Analyse peri-operative process	(49)
Stewart	2015	Ghana	Cross-sectional survey	Assess trauma capacity	(61)
Van Amelsfoort	2010	Malawi	Mixed methods	Describe the training and experiences of clinical officers	(27)
Van den Akker	2009	Malawi	Mixed methods	Evaluate the impact of audits in reducing the incidence of uterine ruptures	(62)

This review covered 16 countries with a few articles presenting data from multiple countries(4,17,18) and others giving a regional perspective(19,20). Ghana, Malawi and Rwanda were the three countries most represented in the articles selected, partly because of research projects in collaboration with researchers from High Income Countries (HIC) (Table 2)

For peer review only - http://bmjopen.bmj.com/site/about/guidelines.xhtml

Table 2: Distribution of articles per country

Country	Number of articles
Burkina Faso	3
Cameroon	2
Ethiopia	2
Ghana	11
Kenya	2
Liberia	2
Malawi	10
Mozambique	1
Namibia	1
Niger	1
Rwanda	7
Senegal	1
Sub Saharan Africa (regional perspective)	2
Tanzania	5
Uganda	4
Zambia	2
Zimbabwe	2

3. Characteristics of district hospitals:

District hospitals described in this review included a variety of institutions (see Table 3). In our review, the two smallest DH had 21 beds reported in Liberia and Ghana by Sherman et al(21) and Choo et al(22) respectively. The biggest DH had 371 beds and was reported by Fehr et al in Tanzania(23). District hospitals served rural or urban populations, with some serving both groups. Although most were owned by the government, mission linked district hospitals also featured in several papers. On some occasions, hospitals received support from visiting surgeons or NGOs, e.g. in Rwanda, government owned district hospitals received support from Partners in Health(14).

Table 3: Distribution of articles per size of district hospitals

Size of district hospitals (in number of beds)	Number of articles
<20	0
20-50	2
51-100	8
101-300	9
>300	2
Not specified	33

4. Scope of surgical operations

The majority of articles mentioned obstetrics/gynaecology and general surgery operations (Table 4). Indeed, the three most frequently reported procedures were caesarean section, laparotomy and hernia repair. However, a wide range of other surgical procedures were mentioned across the literature. These were mainly in orthopaedics, plastic surgery or neurosurgery and may have involved visiting specialists (Table 4). District hospitals reported in the literature handled both emergency procedures such as caesarean sections,

For peer review only - http://bmjopen.bmj.com/site/about/guidelines.xhtml

appendicectomies and treatment of burns; and elective procedures such as hernia repairs and some even more complex procedures such as direct vision internal urethrotomy(24) and hip replacement(13). Despite the high prevalence of traumatic brain injury in sub Saharan Africa(25) and the fact that cranial burr holes are included in the WHO book "Surgical Care at the District Hospital"(26), only three articles mentioned burr holes.

Table 4: Distribution of articles per surgical operations

Surgical Procedures	Number of articles
General Surgery	
Hernia Repair	26
Laparotomy	21
Appendicectomy	10
Abscess	7
Hydrocele repair	7
Thyroid surgery	5
Colectomy	4
Chest tube insertion	4
Prostatectomy	4
Cricothyroidotomy	3
Colostomy	2
Lipoma	2
Tracheostomy	2
Fibroma	1
Urethrotomy	1
Vascular repair	1
Bladder stone surgery	1
Obstetrics and Gynaecology	
Caesarean section	30
Salpingectomy (ectopic pregnancy)	10
Hysterectomy	9
Evacuation of Retained Products of Conception	7
Tubal Ligation	5
Fistula Repair	3
Cervical tear repair	2
Breast surgery	1
Orthopaedics	
Amputation	10
Open Fracture repair	8
Closed Fracture repair	5
Arthrotomy	3

For peer review only - http://bmjopen.bmj.com/site/about/guidelines.xhtml

For peer review only

Osteotomy	2
Total Hip replacement	1
Bipolar Hemiarthroplasty	1
Neurosurgery	
Major neurosurgery	1
Burr holes	3
Plastics	
Skin grafting	7
Debridement	5
Burn	4
Cleft lip/palate surgery	3
Contracture release	2
Escharotomy	1

5. Surgical volume

Only 13 articles provided enough data to be able to estimate the number of operations per year per district hospital performed. In some cases, there was not enough information to discriminate between major and minor operations, therefore the choice was made to present all operations combined. Using a simple formula; the number of operations, period of years and number of district hospitals, an average of the number of operations per district hospital per year was calculated (Table 5). Galukande et al(4) and LeBrun et al(18) contributed several reports on different district hospitals while Van Amelsfoort et al(27) contributed two reports on the same district hospital during two different time periods.

As seen in tables 5 and 6, the surgical volume of district hospitals varied from circa 200 to circa 5000 operations per year, with the majority of hospitals performing between 1001 and 5000 operations per year. Clear limitations to the use of this data is the sample size and selection bias. Of the 13 studies, only six analysed more than five district hospitals in a given area. This data, therefore, cannot solely be used as a general indicator for the productivity of district hospitals in the countries represented.

Table 5: Surgical volume (all reported cases) in number of operations per year per district hospital

First author	Study setting	Total number of operations	Years	Number of district hospitals	Estimated number of operations per district hospital per year *(to the nearest unit)*
Abdullah~ (28)	Ghana	-	1	10	774
Damien (29)	Ghana	1,391	5	1	278
Galukande (4)	Tanzania	980	1	1	980

For peer review only - http://bmjopen.bmj.com/site/about/guidelines.xhtml

Galukande (4)	Tanzania	2,045	1	1	2,045
Galukande (4)	Mozambique	601	1	1	601
Galukande (4)	Mozambique	256	1	1	256
Galukande (4)	Uganda	1,484	1	1	1,484
Galukande (4)	Uganda	248	1	1	248
Galukande (4)	Uganda	239	12	1	239
Galukande (4)	Uganda	1,835	1	1	1,835
Gyedu (30)	Ghana	143,750	1	48	2,995
Lofgren $ (31)	Uganda	-	-	2	5,018
Lavy (32)	Malawi	25,053	1	21	1,193
LeBrun* (18)	Uganda	-	1	1	1,296
LeBrun* (18)	Rwanda	-	1	1	2,052
LeBrun* (18)	Liberia	-	1	1	696
LeBrun* (18)	Ethiopia	-	1	1	538
Nordberg~ (33)	Kenya	-	1	5	5,233
Notrica* (34)	Rwanda	-	-	-	2,052
Ottaway (35)	Namibia	737	0.58	4	316
Petroze (36)	Rwanda	71,432	1	41	1,742
Sani (37)	Niger	544	1	3	181
Van Amelsfoort (27)	Malawi	19,644	1	17	1,156
Van Amelsfoort (27)	Malawi	18,524	1	17	1,090

* Reported in the article as average number per year per district hospital
~ The article reported numbers for both major and minor operations and we calculated the total number of procedures.
$ This article reported that the number of major operations was estimated over a 4 month period while the number of minor operations was estimated over a 3 month period.

Table 6: Number of publications reporting the number of operations per year per district hospital

Number of operations on average per district hospital over 1 year	Number of publications
<100	0
100-500	6
501-1000	5
1001-5000	11
>5000	2

6. Types of anaesthesia

For peer review only - http://bmjopen.bmj.com/site/about/guidelines.xhtml

Out of 52 articles, 16 contained information about the type of anaesthesia used in the district hospitals studied. General anaesthesia was mentioned by 15 articles(9,11,13,17,21–32) and spinal anaesthesia by 10 articles(13,16,21,22,31,35,39–41,44). Local anaesthesia and regional blocks were mentioned by 4 articles(14,16,21,31).

7. Surgical and anaesthesia providers

In different countries, different cadres of healthcare professionals are responsible for providing surgical care in district hospitals. In Ethiopia, Ramos et al reported the presence of a specialist surgeon(12). In Ghana(22,28,30,45–49), Kenya(33), Rwanda(14–16,34,36,42) and Zimbabwe(43) medical officers worked alongside specialist surgeons or obstetrician/gynaecologists to provide surgical care. In Namibia(35) and Niger(37), the presence of medical officers as sole surgical providers was reported. Non-physician clinicians provided surgical care in Burkina Faso(13,41,50), Liberia(21), Malawi(38,44,51–53), Senegal(54), Tanzania(17,55), Uganda(31,56) and Zambia(57). In our review, there was no clear majority in terms of the number of articles mentioning each cadre (Table 5).

Similarly, anaesthesia was performed by different cadres in different countries. Articles reported the presence of a physician anaesthetist in district hospitals in Burkina Faso(13) and Ghana(49). Medical officers providing anaesthesia on their own were reported in Namibia(35). Otherwise, the most mentioned anaesthesia providers are non-physician clinicians trained in anaesthesia (Table 6). Their presence has been reported in Burkina Faso(50), Ethiopia(12), Ghana(22), Liberia(21), Malawi(44)(20), Rwanda(34)(36), Senegal(54), Tanzania(17), Uganda(31), Zambia(57) and Zimbabwe(43)(39). There are also untrained non-physician clinicians providing anaesthesia in Liberia(21), Malawi(44)(20), Rwanda(34)(36), Zimbabwe(43)(39) alongside trained personnel.

Table 7: Number of publications reporting surgical providers

Surgical Providers	Number of Publications
Specialist Surgeon/Obstetrician	5
Specialist Surgeon/Obstetrician & Medical Officers	9
Medical Officers	8
Medical Officers & Non-Physician Clinicians	4
Specialist Surgeon/Obstetrician & Non-Physician Clinicians	1
Non-Physician Clinicians	5
Specialists & Medical Officers & Non-Physician Clinicians	8

Table 8: Number of publications reporting anaesthesia providers

Anaesthesia Providers	Number of Publications
Physician Anaesthetists	2
Medical Officers	1
Medical Officer & Non-Physician Clinicians	1

For peer review only - http://bmjopen.bmj.com/site/about/guidelines.xhtml

Non-Physician Clinicians (trained)	10
Non-Physician Clinicians (trained & untrained)	6

8. Quality of care and surgical outcomes

8.1. Quality of care

Of all articles in our review, only nine articles evaluated the quality of care as part of their study. Choo et al surveyed 10 district hospitals in Ghana about quality improvement activities(58). None of the hospitals surveyed reported an organised quality improvement programme, although this does not mean it was absent, and the only quality improvement activity related to surgery was found in one hospital and related to sterilisation of instruments(58). Other authors echoed these findings by reporting a lack of clinical guidelines for surgery, anaesthesia and labour management(21,50,59) and a lack of staff trained in using them when they were available(50).

To evaluate the quality of caesarean sections, Harfouche et al measured the "decision to incision time" in a district hospital in Malawi and found that it reached "1.69 hours per caesarean section, far exceeding the recommended 30 minutes"(60). They explained their findings by a "lack of operating theatre space and high patient volume" but also by an inefficient system to transfer patients to the operating theatre(60).

Another major challenge to quality care in district hospitals in sub-Saharan Africa is the shortage of trained surgical and anaesthesia staff. Stewart et al(61) explained that shortage in staff resulted in unavailability of service during nights and weekends. Choo et al found that medical officers in Ghana had limited exposure to surgery during their training(22). Harfouche et al reported that while clinical officers performed most caesarean sections at district level in Malawi, they were not sufficiently trained in performing emergency hysterectomies(60). Positive outcomes were observed in cases where trained staff were present: Hall et al reported an "association between the presence of OBGYNs in district hospitals in Ghana and an increase in evidence-based maternal care practices, as well as moderate improvement in maternal mortality and stillbirth rates"(45).

Some authors suggested interventions to improve the quality of care. Van den Akker et al suggested audit as a "simple intervention requiring little technology" that is able to improve quality of care(62). They reported a decrease in 68% of uterine ruptures in a district hospital in Malawi after implementing recommendations inferred from an audit. Recommendations included training of staff, better documentation and involvement of hospital management(62). Other authors insisted on the need for monitoring safety and quality of care in district hospitals as a first step toward improving quality(58)(31).

However, Smiley et al reported care of exceptional quality in a "63 bed district hospital" named the "Volta River Authority Hospital" in Ghana(49). In their study they surveyed staff and patients about their perception of quality of care and conducted process mapping of surgical and obstetric care. They found that "over 80% of employees across a variety of perioperative roles held a positive view of teamwork and safety climate within the institution" and "surgical patients who were surveyed gave similarly positive indications of overall satisfaction"(49). The perception of staff and patients was complemented by the

For peer review only - http://bmjopen.bmj.com/site/about/guidelines.xhtml

observations of researchers during the process mapping. The WHO safety checklist was used in all operations witnessed and the authors reported the existence of a hospital wide morbidity and mortality conference as well as a safety training for new staff(49).

8.2. Surgical outcomes

The most commonly used indicators of outcome and quality were peri-operative complications and peri-operative mortality. 15 articles reported information about peri-operative complications(13–16,23,35,40,44,51,52,59,60,62–64), the most reported being surgical site infection. 15 articles included information about peri-operative mortality(10-12,15,16,18,20,22,23,26,32,36,41,49,50), the most reported causes of death were sepsis, haemorrhage and anaesthesia complications.

In surgical obstetric care, the most reported complications were ruptured uterus, post-partum haemorrhage and wound dehiscence(44,60,62). The most reported cause of maternal maternity was ruptured uterus(15,18,20,23,26,30,32,49)

Discussion:

Summary of findings:

Although it varies by country, our review found that district hospitals provide mainly emergency obstetric and general surgery and, in some cases, elective surgery covering a wide range of surgical specialties. The literature reports that, most often, surgical procedures are done under general or spinal anaesthesia.

Many of the procedures performed are by trained non-physician clinicians and medical officers. There is a paucity of data regarding quality of surgical care, surgical volume and peri-operative morbidity and mortality. However, the most reported complications are infections and post-partum haemorrhage.

Strengths and Limitations:

This is the first review to systematically assess the literature for the scope, volume and quality of surgery in district hospitals in sub-Saharan Africa, to our knowledge. To minimise subjectivity, the article selection and data extraction were conducted independently by two authors. To mitigate a language bias, we did not limit our search to English, but included French and Portuguese. However, this study has some limitations.

First, we could have missed relevant literature that did not specifically use the terminology ‘district hospital’ but instead used other terms such as ‘rural hospital’.

Second, our study is prone to some publication bias as it is based only on information present in published literature. It is unlikely that institutions that are not actively auditing activity would present it for publication. And even those district hospitals that do collect information of relevance to this study are unlikely to submit it for publication, unless they have a member of staff with an academic interest, and thus are unlikely to be represented in published literature.

For peer review only - http://bmjopen.bmj.com/site/about/guidelines.xhtml

For peer review only

Finally, our results are limited by the heterogeneity in how articles reported indicators such as personnel training level, surgical volume in terms of minor and major operations, complications, outcomes, and peri-operative mortality. If surgical indicators were used, these were usually non-standard, and the core surgical indicators recommended by the Lancet Commission on Global Surgery(65) were not routinely used.

Impact for future research

Our study is a snapshot of two decades of published surgical activity in DHs. We had aimed to compare it with other similar studies, but we found none that was appropriate. We feel that there is a challenge and an imperative for DHs to collect information on key indicators so that standards can be measured, and quality improvement encouraged. Our preference is to start with the indicators recommended by the Lancet Commission on Global Surgery. (65)

Impact for policy

It is not the role of this paper to determine or influence surgical policy for Ministries of Health in sub-Saharan Africa, however its findings indicate areas of need in both recording of surgical activity, and quality improvement practice for the region.

Conclusion:

District hospitals play a significant role in surgical care in sub-Saharan African countries.

This scoping review has provided a broad but incomplete data set on the current provision of surgical care in district hospitals in sub-Saharan Africa as reported in published studies. We recommend to researchers to conduct more primary surgical systems research in district hospitals to report on surgical activity, using core indicators suggested by the Lancet Commission on Global Surgery that will improve comparability.

Authors Contribution:

Zineb Bentounsi, MD, MSc: investigation, data curation, methodology, formal analysis, writing-original draft, writing-review and editing

Sharaf Sheik Ali, MBBS, BSc: investigation, formal analysis, writing- original draft, writing-review and editing

Grace Le, MA: project administration, investigation, writing-review and editing

Chris Lavy, MBBS, MD: conceptualization, supervision, writing-review and editing

Data Sharing Statement: Data available upon request

Patient and public involvement: No patient involved

For peer review only - http://bmjopen.bmj.com/site/about/guidelines.xhtml

Conflict of Interest: Authors have no conflict of interest.

Funding Statement: This work was supported by SURG Africa through the European Union Horizon 2020 programme Grant Agreement no. 733391.

References:

1. Luboga S, Macfarlane SB, von Schreeb J, Kruk ME, Cherian MN, Bergström S, et al. Increasing access to surgical services in sub-saharan Africa: priorities for national and international agencies recommended by the Bellagio Essential Surgery Group. PLoS Med [Internet]. 2009/12/22. 2009 Dec;6(12):e1000200–e1000200. Available from: https://pubmed.ncbi.nlm.nih.gov/20027218
2. Mulligan J-A, Fox-Rushby JA, Adam T, Johns B, Mills A. Unit Costs of Health Care Inputs in Low and Middle Income Regions. 2003.
3. Pollock JD, Love TP, Steffes BC, Thompson DC, Mellinger J, Haisch C. Is it possible to train surgeons for rural Africa? A report of a successful international program. World J Surg. 2011 Mar 30;35(3):493–9.
4. Galukande M, von Schreeb J, Wladis A, Mbembati N, de Miranda H, Kruk ME, et al. Essential surgery at the district hospital: a retrospective descriptive analysis in three African countries. PLoS Med. 2010 Mar;7(3):1–10.
5. Roberts G, Roberts C, Jamieson A, Grimes C, Conn G, Bleichrodt R. Surgery and Obstetric Care are Highly Cost-Effective Interventions in a Sub-Saharan African District Hospital: A Three-Month Single-Institution Study of Surgical Costs and Outcomes. World J Surg. 2016;40:14–20.
6. Rajbhandari R, McMahon DE, Rhatigan JJ, Farmer PE. The neglected hospital - The district hospital's central role in global health care delivery [Internet]. Vol. 382, New England Journal of Medicine. Massachussetts Medical Society; 2020 [cited 2020 Jun 3]. p. 397–400. Available from: http://www.nejm.org/doi/10.1056/NEJMp1911298
7. Grimes C, Lavy C. A plea for investment in district hospitals [Internet]. Vol. 376, The Lancet. Elsevier; 2010 [cited 2020 Jun 30]. p. 2073. Available from: http://www.thelancet.com/article/S0140673610622933/fulltext
8. Arksey H, O'Malley L. Scoping studies: towards a methodological framework. Int J Soc Res Methodol. 2005;8(1):19–32.
9. Levac D, Colquhoun H, O'Brien KK. Scoping studies: Advancing the methodology. Implement Sci. 2010;5(1):1–9.
10. Ouzzani M, Hammady H, Fedorowicz Z, Elmagarmid A. Rayyan-a web and mobile app for systematic reviews. Syst Rev [Internet]. 2016;5(1):210. Available from: http://dx.doi.org/10.1186/s13643-016-0384-4
11. QSR International. NVivo Qualitative Data Analysis Software- Version 12. 2018.
12. Ramos JM, Abate N, Reyes F, Belate W, Mohammed F, Gorgolas M. Thyroid surgery in a district hospital in Southern Ethiopia: experience from a rural center. 2013;37(7). Available from: http://www.ncbi.nlm.nih.gov/pubmed/23564217
13. Dossche L, Noyez JF, Ouedraogo W, Kalmogho E. Establishment of a hip replacement project in a district hospital in Burkina Faso: analysis of technical problems and peri-operative complications. Bone Joint J [Internet]. 2014;96(2):177–80. Available from: http://www.ncbi.nlm.nih.gov/pubmed/24493181
14. Muhirwa E, Habiyakare C, Hedt-Gauthier BL, Odhiambo J, Maine R, Gupta N, et al. Non-Obstetric Surgical Care at Three Rural District Hospitals in Rwanda: More Human Capacity and Surgical Equipment May Increase Operative Care. World J Surg.

For peer review only - http://bmjopen.bmj.com/site/about/guidelines.xhtml

2016;40(9):2109–16.
15. Nkurunziza T, Kateera F, Sonderman K, Gruendl M, Nihiwacu E, Ramadhan B, et al. Prevalence and predictors of surgical-site infection after caesarean section at a rural district hospital in Rwanda. Br J Surg [Internet]. 2019;106(2):e121–8. Available from: http://www.ncbi.nlm.nih.gov/pubmed/30620071
16. Mpirimbanyi C, Nyirimodoka A, Lin Y, Hedt-Gauthier BL, Odhiambo J, Nkurunziza T, et al. Emergency general surgery in Rwandan district hospitals: a cross-sectional study of spectrum, management, and patient outcomes. 2017;17(1):121.
17. Gajewski J, Pittalis C, Lavy C, Borgstein E, Bijlmakers L, Mwapasa G, et al. Anesthesia Capacity of District-Level Hospitals in Malawi, Tanzania, and Zambia: A Mixed-Methods Study. Anesth Analg [Internet]. 2019 Apr 1 [cited 2020 Apr 28];130(4):845–53. Available from: http://ovidsp.ovid.com/ovidweb.cgi?T=JS&PAGE=reference&D=ovftu&NEWS=N&AN=00000539-900000000-96017
18. LeBrun DG, Chackungal S, Chao TE, Knowlton LM, Linden AF, Notrica MR, et al. Prioritizing essential surgery and safe anesthesia for the Post-2015 Development Agenda: operative capacities of 78 district hospitals in 7 low- and middle-income countries. Surgery [Internet]. 2014;155(3):365–73. Available from: http://www.ncbi.nlm.nih.gov/pubmed/24439745
19. Grimes CE, Law RSL, Borgstein ES, Mkandawire NC, Lavy CBD. Systematic review of met and unmet need of surgical disease in rural sub-Saharan Africa. World J Surg [Internet]. 2012;36(1):8–23. Available from: http://www.ncbi.nlm.nih.gov/pubmed/22057752
20. Henry JA, Bem C, Grimes C, Borgstein E, Mkandawire N, Thomas WEGG, et al. Essential surgery: the way forward. World J Surg. 2015;39(4):822–32.
21. Sherman L, Clement P, Cherian M, Ndayimirije N, MD W-L, Noel L, et al. Implementing Liberia's Poverty Reduction Strategy: An Assessment of Emergency and Essential Surgical Care. Arch Surg [Internet]. 2011;146(1):35–9. Available from: http://ovidsp.ovid.com/ovidweb.cgi?T=JS&PAGE=reference&D=ovftl&NEWS=N&AN=00000853-201101000-00008
22. Choo S, Perry H, Hesse AAJ, Abantanga F, Sory E, Osen H, et al. Surgical training and experience of medical officers in Ghana's district hospitals. 2011;86(4). Available from: http://www.ncbi.nlm.nih.gov/pubmed/21346502
23. Fehr J, Hatz C, Soka I, Kibatala P, Urassa H, Smith T, et al. Risk Factors for Surgical Site Infection in a Tanzanian District Hospital: A Challenge for the Traditional National Nosocomial Infections Surveillance System Index. Infect Control Hosp Epidemiol [Internet]. 2006;27(12):1401–4. Available from: http://ovidsp.ovid.com/ovidweb.cgi?T=JS&PAGE=reference&D=ovfth&NEWS=N&AN=00004848-200612000-00019
24. Ngaroua, Eloundou NJ, Djibrilla Y, Asmaou O, Mbo AJ. Aspects épidémiologiques, cliniques et prise en charge de sténose urétrale chez l'adulte dans un Hôpital de District de Ngaoundéré, Cameroun. Pan Afr Med J. 2017;26.
25. Hyder AA, Wunderlich CA, Puvanachandra P, Gururaj G, Kobusingye OC. The impact of traumatic brain injuries: A global perspective. Vol. 22, NeuroRehabilitation. IOS Press; 2007. p. 341–53.
26. World Health Organization. Surgical Care at the District Hospital. 2003.
27. Van Amelsfoort JJC, Van Leeuwen PAM, Jiskoot P, Ratsma YEC. Surgery in Malawi – the training of clinical officers. Trop Doct. 2010;40(2):74–6.
28. Abdullah F, Choo S, Hesse AAJ, Abantanga F, Sory E, Osen H, et al. Assessment of surgical and obstetrical care at 10 district hospitals in Ghana using on-site interviews. J

For peer review only - http://bmjopen.bmj.com/site/about/guidelines.xhtml

Surg Res [Internet]. 2011;171(2):461–6. Available from: http://www.ncbi.nlm.nih.gov/pubmed/20691981

29. Damien P, Nabare C, Baiden F, Kwara E, Apanga S, Etego-Amengo S. How are surgical theatres in rural Africa utilized? A review of five years of services at a district hospital in Ghana. Trop Doct [Internet]. 2011;41(2):91–5. Available from: http://www.ncbi.nlm.nih.gov/pubmed/21421886
30. Gyedu A, Stewart B, Gaskill C, Boakye G, Appiah-Denkyira E, Donkor P, et al. Improving Benchmarks for Global Surgery: Nationwide Enumeration of Operations Performed in Ghana. Ann Surg. 2018;268(2):282–8.
31. Löfgren J, Kadobera D, Forsberg BC, Mulowooza J, Wladis A, Nordin P. District-level surgery in Uganda: Indications, interventions and perioperative mortality. Surg (United States). 2015;158(1):7–16.
32. Lavy C, Tindall A, Steinlechner C, Mkandawire N, Chimangeni S. Surgery in Malawi - a national survey of activity in rural and urban hospitals. Ann R Coll Surg Engl [Internet]. 2007;89(7):722–4. Available from: http://ovidsp.ovid.com/ovidweb.cgi?T=JS&PAGE=reference&D=ovfti&NEWS=N&AN=00000639-200710000-00025
33. Nordberg E, Mwobobia I, Muniu E. Major and minor surgery output at district level in Kenya: review and issues in need of further research. Afr J Health Sci. 2002;9(1):17–25.
34. Notrica MR, Evans FM, Knowlton LM, Kelly McQueen KA. Rwandan surgical and anesthesia infrastructure: A survey of district hospitals. World J Surg. 2011;35(8):1770–80.
35. Ottaway AJ, Kabongo L. Prospective Observational Study of Intraoperative Anesthetic Events in District Hospitals in Namibia. 2018;126(2):632–8. Available from: http://www.ncbi.nlm.nih.gov/pubmed/29261548
36. Petroze RT, Nzayisenga A, Rusanganwa V, Ntakiyiruta G, Calland JF. Comprehensive national analysis of emergency and essential surgical capacity in Rwanda. 2012;99(3). Available from: http://www.ncbi.nlm.nih.gov/pubmed/22237597
37. Sani R, Nameoua B, Yahaya A, Hassane I, Adamou R, Hsia RY, et al. The impact of launching surgery at the district level in Niger. World J Surg. 2009;33(10):2063–8.
38. Gajewski J, Bijlmakers L, Mwapasa G, Borgstein E, Pittalis C, Brugha R. "I think we are going to leave these cases". Obstacles to surgery in rural Malawi: a qualitative study of provider perspectives. Trop Med Int Heal [Internet]. 2018 Oct 1 [cited 2020 Apr 21];23(10):1141–7. Available from: http://ovidsp.ovid.com/ovidweb.cgi?T=JS&PAGE=reference&D=ovftt&NEWS=N&AN=00060771-201810000-00013
39. Lonnee H, Madzimbamuto F, Erlandsen O, Vassenden A, Chikumba E, Dimba R, et al. Anesthesia for Cesarean Delivery: A Cross-Sectional Survey of Provincial, District, and Mission Hospitals in Zimbabwe. Anesth Analg [Internet]. 2018;126(6):2056–64. Available from: http://ovidsp.ovid.com/ovidweb.cgi?T=JS&PAGE=reference&D=ovftt&NEWS=N&AN=00000539-201806000-00040
40. Ouédraogo CM, Ouédraogo A, Ouattara A, Lankoandé J. [Cesarean deliveries in a district hospital in Ouagadougou. Epidemiological, clinical, and prognostic study of 3381 cases]. Med Sante Trop [Internet]. 25(2):194–9. Available from: http://www.ncbi.nlm.nih.gov/pubmed/26081260
41. Ouédraogo CM, Ouattara A, Ouédraogo A, Bikienga M, Lankoandé J. Amélioration de l'offre des soins obstétricaux par la délégation des tâches en milieu Africain Exemple de l'hôpital du district sanitaire de Bogodogo à Ouagadougou, Burkina Faso. Med

For peer review only - http://bmjopen.bmj.com/site/about/guidelines.xhtml

Sante Trop. 2015;25(3):280–4.
42. Petroze RT, Mehtsun W, Nzayisenga A, Ntakiyiruta G, Sawyer RG, Calland JF, et al. Ratio of cesarean sections to total procedures as a marker of district hospital trauma capacity. World J Surg. 2012;36(9):2074–9.
43. Rutgers RAK, Van Eygen L. Mortality related to caesarean section in rural Matebeleland North Province, Zimbabwe. Cent Afr J Med [Internet]. 54(5):24–7. Available from: http://www.ncbi.nlm.nih.gov/pubmed/21650076
44. Fenton PM, Whitty CJM, Reynolds F. Caesarean section in Malawi: prospective study of early maternal and perinatal mortality. BMJ. 2003;327(7415):587.
45. Hall E, Sevugu J, Danso K, Adomako J, Peltzman T, Anderson FJ. A retrospective analysis of the impact of an obstetrician on delivery and care outcomes at four district hospitals in Ghana. Int J Gynaecol Obstet [Internet]. 2015;131(1):100–1. Available from: http://www.ncbi.nlm.nih.gov/pubmed/26143230
46. Luo EM, Opare-Ado HS, Adomako J, Danso KA, Peltzman T, Anderson FWJJ. Completing the Maternal Care Team: OB/GYN Expertise at Rural District Hospitals in Ghana, a Qualitative Study. 2018;22(7):1085–91. Available from: http://www.ncbi.nlm.nih.gov/pubmed/29520728
47. McCord C. Facilities for Surgery Exist in Ghana, but Skills Are Lacking: Comment on "Assessing the Surgical and Obstetrics-Gynecology Workload of Medical Officers". Arch Surg [Internet]. 2012;147(6):548–9. Available from: http://ovidsp.ovid.com/ovidweb.cgi?T=JS&PAGE=reference&D=ovftm&NEWS=N&AN=00000853-201206000-00013
48. Mehtsun WT, Weatherspoon K, McElrath L, Chima A, Torsu VEK, Obeng ENB, et al. Assessing the surgical and obstetrics-gynecology workload of medical officers: findings from 10 district hospitals in Ghana. Arch Surg [Internet]. 2012;147(6):542–8. Available from: http://www.ncbi.nlm.nih.gov/pubmed/22786541
49. Smiley K, Ofori L, Spangler C, Acquaah-Arhin R, Deh D, Enos J, et al. Safety Culture and Perioperative Quality at the Volta River Authority Hospital in Akosombo, Ghana. 2019;43(1):16–23. Available from: http://www.ncbi.nlm.nih.gov/pubmed/30109388
50. Compaoré GD, Sombié I, Ganaba R, Hounton S, Meda N, Brouwere V De, et al. Readiness of district and regional hospitals in Burkina Faso to provide caesarean section and blood transfusion services: a cross-sectional study. BMC Pregnancy Childbirth. 2014;14:158.
51. Gajewski J, Borgstein E, Bijlmakers L, Mwapasa G, Aljohani Z, Pittalis C, et al. Evaluation of a surgical training programme for clinical officers in Malawi. BJS [Internet]. 2019;106(2):e156–65. Available from: https://onlinelibrary.wiley.com/doi/abs/10.1002/bjs.11065
52. Gajewski J, Conroy R, Bijlmakers L, Mwapasa G, McCauley T, Borgstein E, et al. Quality of Surgery in Malawi: Comparison of Patient-Reported Outcomes After Hernia Surgery Between District and Central Hospitals. World J Surg. 2018;42(6):1610–6.
53. Henry J, Frenkel E, Borgstein E, Mkandawire N, Goddia C. Surgical and anaesthetic capacity of hospitals in Malawi: key insights. Heal Policy Plan [Internet]. 2015;30(8):985–94. Available from: http://ovidsp.ovid.com/ovidweb.cgi?T=JS&PAGE=reference&D=ovftq&NEWS=N&AN=00003845-201510000-00005
54. De Brouwere V, Dieng T, Diadhiou M, Witter S, Denerville E. Task shifting for emergency obstetric surgery in district hospitals in Senegal. Reprod Health Matters. 2009;17(33):32–44.
55. McCord C, Mbaruku G, Pereira C, Nzabuhakwa C, Bergstrom S. The quality of

For peer review only

emergency obstetrical surgery by assistant medical officers in Tanzanian district hospitals. Health Aff (Millwood) [Internet]. 28(5):w876-85. Available from: http://www.ncbi.nlm.nih.gov/pubmed/19661113

56. Dresser, Caleb; Periyanayagam, Usha; Dreifuss, Brad; Wangoda, Robert; Luyimbaazi, Julius; Bisanzo MGECCIGECC. Management and Outcomes of Acute Surgical Patients at a District Hospital in Uganda with Non-physician Emergency Clinicians. World J Surg [Internet]. 2017 [cited 2020 Apr 28]; Available from: https://rayyan.qcri.org/fulltexts/320248

57. Cheelo M, Brugha R, Bijlmakers L, Kachimba J, McCauley T, Gajewski J. Surgical Capacity at District Hospitals in Zambia: From 2012 to 2016. World J Surg. 2018 Nov 1;42(11):3508–13.

58. Choo S, Papandria D, Goldstein SD, Perry H, Hesse AAJ, Abatanga F, et al. Quality improvement activities for surgical services at district hospitals in developing countries and perceived barriers to quality improvement: findings from Ghana and the scientific literature. World J Surg [Internet]. 2013;37(11):2512–9. Available from: http://www.ncbi.nlm.nih.gov/pubmed/23897444

59. Koigi-Kamau R, Kabare LW, Wanyoike-Gichuhi J. Incidence of wound infection after Caesarean delivery in a district hospital in Central Kenya. East Afr Med J. 2005;82(7):357–61.

60. Harfouche M, Hosseinipour M, Kaliti S, Wilkinson J. Quality indicators and outcomes of emergency caesarean deliveries at a district-level maternity hospital. Afr J Reprod Health. 2015;19(3):61–7.

61. Stewart BT, Quansah R, Gyedu A, Ankomah J, Donkor P, Mock C. Strategic Assessment of Trauma Care Capacity in Ghana. World J Surg. 2015;39(10):2428–40.

62. van den Akker T, Mwagomba B, Irlam J, van Roosmalen J. Using audits to reduce the incidence of uterine rupture in a Malawian district hospital. Int J Gynaecol Obstet [Internet]. 2009;107(3):289–94. Available from: http://www.ncbi.nlm.nih.gov/pubmed/19846089

63. Kenfack B, Noubom M, Bongoe A, Tsatedem FA, Ngono M, Tsague GN, et al. [Ectopic pregnancy in a semi-rural area in Africa: Epidemiological, clinical and therapeutic aspects about a series of 74 cases treated at the District Hospital of Sangmelima in southern Cameroon]. Pan Afr Med J. 2012;13:71.

64. Fehr J, Hatz C, Soka I, Kibatala P, Urassa H, Battegay M, et al. Antimicrobial prophylaxis to prevent surgical site infections in a rural sub-Saharan hospital. Clin Microbiol Infect [Internet]. 2006;12(12):1224–7. Available from: http://ovidsp.ovid.com/ovidweb.cgi?T=JS&PAGE=reference&D=ovfth&NEWS=N&AN=00125357-200612000-00012

65. Meara, J. G., Leather, A. J., Hagander, L., Alkire, B. C., Alonso, N., Ameh EA. Global Surgery 2030: evidence and solutions for achieving health, welfare, and economic development. Lancet. 2015;386(9993):569–624.

For peer review only

PRISMA 2009 Flow Diagram

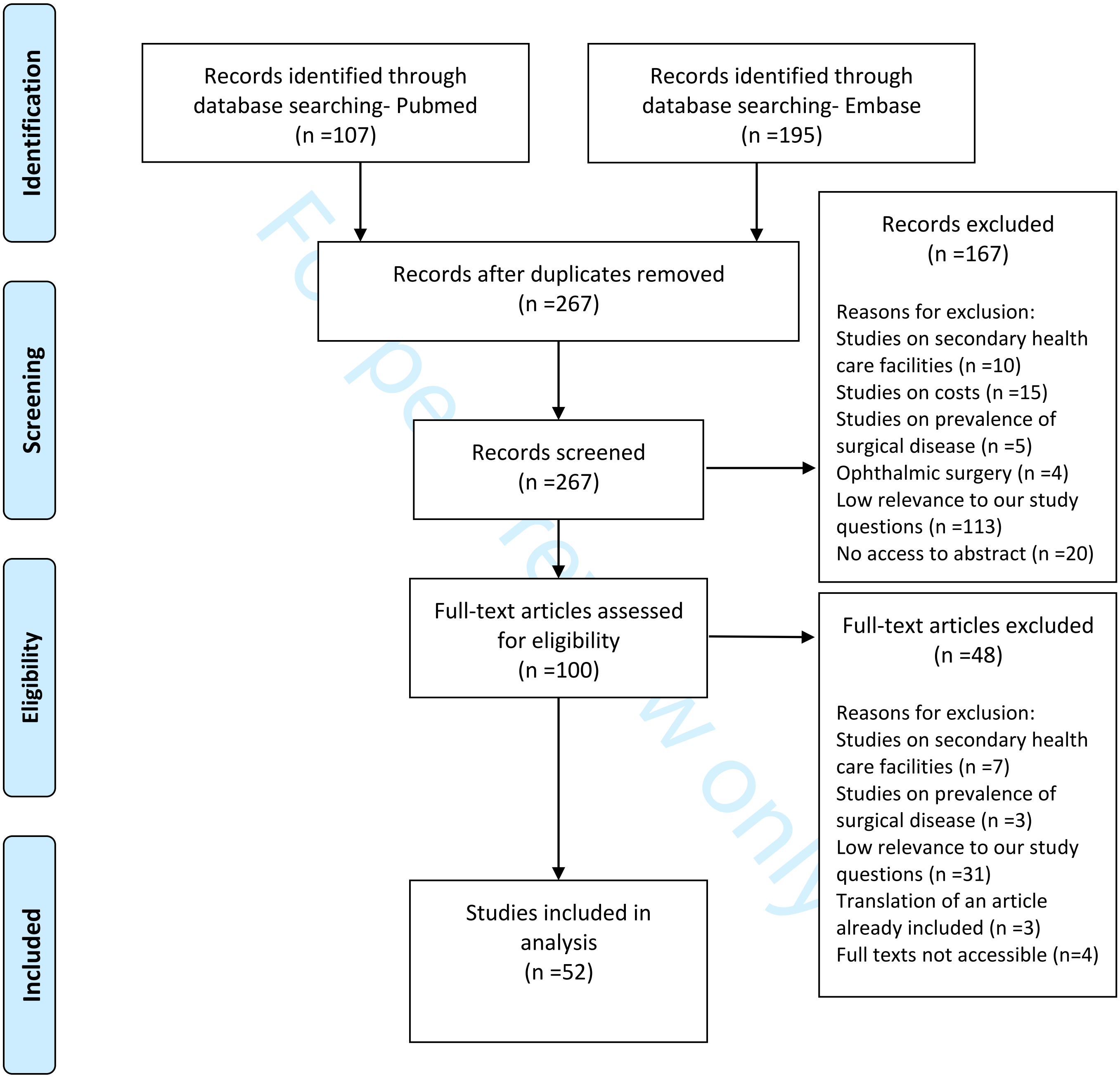

From: Moher D, Liberati A, Tetzlaff J, Altman DG, The PRISMA Group (2009). *P*referred *R*eporting *I*tems for *S*ystematic Reviews and *M*eta-*A*nalyses: The PRISMA Statement. PLoS Med 6(7): e1000097. doi:10.1371/journal.pmed1000097

For more information, visit www.prisma-statement.org.

For peer review only - http://bmjopen.bmj.com/site/about/guidelines.xhtml

Data extraction form for the scoping review

Surgical care in District Hospitals in sub Saharan Africa: a Scoping Review

* Required

1. First Author *

2. Year of publication *

3. Year of data collection (latest date to be chosen) *

4. Type of study/Research Method *

For peer review only - http://bmjopen.bmj.com/site/about/guidelines.xhtml

5. Country *

Check all that apply.

- [ ] Angola
- [ ] Burundi
- [ ] DRC
- [ ] Cameroon
- [ ] Central African Republic
- [ ] Chad
- [ ] Republic of the Congo
- [ ] Equatorial Guinea
- [ ] Gabon
- [ ] Kenya
- [ ] Nigeria
- [ ] Rwanda
- [ ] Sao Tome
- [ ] Tanzania
- [ ] Uganda
- [ ] Sudan
- [ ] South Sudan
- [ ] Djibouti
- [ ] Eritrea
- [ ] Ethiopia
- [ ] Somalia
- [ ] Botswana
- [ ] Comores
- [ ] Lesotho
- [ ] Madagascar
- [ ] Malawi
- [ ] Mauritius
- [ ] Mozambique
- [ ] Namibia
- [ ] Seychelles
- [ ] South Africa
- [ ] Swaziland
- [ ] ZAMBIA
- [ ] Zimbabwe
- [ ] Benin
- [ ] Mali
- [ ] Burkina Faso
- [ ] Cape Verde

For peer review only - http://bmjopen.bmj.com/site/about/guidelines.xhtml

- [ ] Ivory Cost
- [ ] Gambia
- [ ] Ghana
- [ ] Guinea
- [ ] Guinea-Bissau
- [ ] Liberia
- [ ] Mauritania
- [ ] Niger
- [ ] Senegal
- [ ] Sierra Leone
- [ ] Togo

Other: [ ] ____________________

6. Specialty *

Check all that apply.

- [ ] Obstetrics and Gynecology
- [ ] General Surgery
- [ ] Trauma and Orthopedics
- [ ] Head and Neck
- [ ] Urology
- [ ] Anesthesia
- [ ] Neurosurgery
- [ ] Paediatric surgery

Other: [ ] ____________________

7. Name of surgical procedures (please write all procedures mentioned) or write NA if none *

8. Surgical Provider *

Check all that apply.

- [ ] Specialist Surgeon (including OBGY)
- [ ] Medical Officer/GP
- [ ] Non Physician Clinician
- [ ] Nurse/Midwife
- [ ] Not specified

Other: [ ] ______________________

9. Type of Anaesthesia *

Check all that apply.

- [ ] General
- [ ] Regional Block
- [ ] Local
- [ ] Spinal
- [ ] Not specified

10. Anaesthesia Provider *

Check all that apply.

- [ ] Physician (anaesthesiologist)
- [ ] Non Physician- trained
- [ ] Non Physician- untrained
- [ ] Not specified
- [ ] Medical officer/Physician non anaesthesiologist

11. Number of operations per year/period (if available)

____

12. Catchment population (if available)

____

13. Equipment (if in a table, just write "table X" here)

14. Patients Outcomes (complications, survival...)

15. What do authors use as indicators for safety? (just list them)

16. What do authors use as indicators for surgical capacity?

17. Was the surgical care provided as a result of a temporary surgical mission or international partnership? *

Mark only one oval.

- Yes
- No
- It is unclear

18. Characteristics of the District Hospital(s) if given: number of bed, number of staff

19. Other important results

20. Recommendations made by authors

This content is neither created nor endorsed by Google.

Google Forms

Preferred Reporting Items for Systematic reviews and Meta-Analyses extension for Scoping Reviews (PRISMA-ScR) Checklist

SECTION	ITEM	PRISMA-ScR CHECKLIST ITEM	REPORTED ON PAGE #
**TITLE**			
Title	1	Identify the report as a scoping review.	Title
**ABSTRACT**			
Structured summary	2	Provide a structured summary that includes (as applicable): background, objectives, eligibility criteria, sources of evidence, charting methods, results, and conclusions that relate to the review questions and objectives.	Page 2
**INTRODUCTION**			
Rationale	3	Describe the rationale for the review in the context of what is already known. Explain why the review questions/objectives lend themselves to a scoping review approach.	Page 3
Objectives	4	Provide an explicit statement of the questions and objectives being addressed with reference to their key elements (e.g., population or participants, concepts, and context) or other relevant key elements used to conceptualize the review questions and/or objectives.	Page 3
**METHODS**			
Protocol and registration	5	Indicate whether a review protocol exists; state if and where it can be accessed (e.g., a Web address); and if available, provide registration information, including the registration number.	Prospero didn't accept to register a scoping review protocol
Eligibility criteria	6	Specify characteristics of the sources of evidence used as eligibility criteria (e.g., years considered, language, and publication status), and provide a rationale.	Page 4
Information sources*	7	Describe all information sources in the search (e.g., databases with dates of coverage and contact with authors to identify additional sources), as well as the date the most recent search was executed.	Page 4
Search	8	Present the full electronic search strategy for at least 1 database, including any limits used, such that it could be repeated.	Page 4 +supplementary material
Selection of sources of evidence†	9	State the process for selecting sources of evidence (i.e., screening and eligibility) included in the scoping review.	Page 4
Data charting process‡	10	Describe the methods of charting data from the included sources of evidence (e.g., calibrated forms or forms that have been tested by the team before their use, and whether data charting was done independently or in duplicate) and any processes for obtaining and confirming data from investigators.	Page 4
Data items	11	List and define all variables for which data were sought and any assumptions and simplifications made.	NA
Critical appraisal	12	If done, provide a rationale for conducting a critical	NA

St. Michael's
Inspired Care. Inspiring Science.

For peer review only - http://bmjopen.bmj.com/site/about/guidelines.xhtml

SECTION	ITEM	PRISMA-ScR CHECKLIST ITEM	REPORTED ON PAGE #
of individual sources of evidence§		appraisal of included sources of evidence; describe the methods used and how this information was used in any data synthesis (if appropriate).	
Synthesis of results	13	Describe the methods of handling and summarizing the data that were charted.	Page 4
**RESULTS**			
Selection of sources of evidence	14	Give numbers of sources of evidence screened, assessed for eligibility, and included in the review, with reasons for exclusions at each stage, ideally using a flow diagram.	Page 5
Characteristics of sources of evidence	15	For each source of evidence, present characteristics for which data were charted and provide the citations.	Pages 5-17
Critical appraisal within sources of evidence	16	If done, present data on critical appraisal of included sources of evidence (see item 12).	NA
Results of individual sources of evidence	17	For each included source of evidence, present the relevant data that were charted that relate to the review questions and objectives.	Pages 5-17
Synthesis of results	18	Summarize and/or present the charting results as they relate to the review questions and objectives.	Page 5-17
**DISCUSSION**			
Summary of evidence	19	Summarize the main results (including an overview of concepts, themes, and types of evidence available), link to the review questions and objectives, and consider the relevance to key groups.	Page 17
Limitations	20	Discuss the limitations of the scoping review process.	Page 17
Conclusions	21	Provide a general interpretation of the results with respect to the review questions and objectives, as well as potential implications and/or next steps.	Page 18
**FUNDING**			
Funding	22	Describe sources of funding for the included sources of evidence, as well as sources of funding for the scoping review. Describe the role of the funders of the scoping review.	Page 19

JBI = Joanna Briggs Institute; PRISMA-ScR = Preferred Reporting Items for Systematic reviews and Meta-Analyses extension for Scoping Reviews.
* Where *sources of evidence* (see second footnote) are compiled from, such as bibliographic databases, social media platforms, and Web sites.
† A more inclusive/heterogeneous term used to account for the different types of evidence or data sources (e.g., quantitative and/or qualitative research, expert opinion, and policy documents) that may be eligible in a scoping review as opposed to only studies. This is not to be confused with *information sources* (see first footnote).
‡ The frameworks by Arksey and O'Malley (6) and Levac and colleagues (7) and the JBI guidance (4, 5) refer to the process of data extraction in a scoping review as data charting.
§ The process of systematically examining research evidence to assess its validity, results, and relevance before using it to inform a decision. This term is used for items 12 and 19 instead of "risk of bias" (which is more applicable to systematic reviews of interventions) to include and acknowledge the various sources of evidence that may be used in a scoping review (e.g., quantitative and/or qualitative research, expert opinion, and policy document).

For peer review only - http://bmjopen.bmj.com/site/about/guidelines.xhtml

From: Tricco AC, Lillie E, Zarin W, O'Brien KK, Colquhoun H, Levac D, et al. PRISMA Extension for Scoping Reviews (PRISMAScR): Checklist and Explanation. Ann Intern Med. 2018;169:467–473. doi: 10.7326/M18-0850.

For peer review only

BMJ Open

Surgical care in District Hospitals in sub-Saharan Africa: a Scoping Review

Journal:	*BMJ Open*
Manuscript ID	bmjopen-2020-042862.R2
Article Type:	Original research
Date Submitted by the Author:	17-Dec-2020
Complete List of Authors:	Bentounsi, Zineb; University of Oxford, Nuffield Department of Orthopaedics, Rheumatology and Musculoskeletal Sciences<br>Sheik-Ali, Sharaf; Oxford University Hospitals NHS Foundation Trust,<br>Drury, Grace; University of Oxford, Nuffield Department of Orthopaedics, Rheumatology and Musculoskeletal Sciences<br>Lavy, Chris; University of Oxford, Nuffiled Department of Orthopaedics, Rheumatology and Musculoskeletal Sciences
**Primary Subject Heading**:	Surgery
Secondary Subject Heading:	Anaesthesia
Keywords:	SURGERY, HEALTH SERVICES ADMINISTRATION & MANAGEMENT, ANAESTHETICS

SCHOLARONE™
Manuscripts

I, the Submitting Author has the right to grant and does grant on behalf of all authors of the Work (as defined in the below author licence), an exclusive licence and/or a non-exclusive licence for contributions from authors who are: i) UK Crown employees; ii) where BMJ has agreed a CC-BY licence shall apply, and/or iii) in accordance with the terms applicable for US Federal Government officers or employees acting as part of their official duties; on a worldwide, perpetual, irrevocable, royalty-free basis to BMJ Publishing Group Ltd ("BMJ") its licensees and where the relevant Journal is co-owned by BMJ to the co-owners of the Journal, to publish the Work in this journal and any other BMJ products and to exploit all rights, as set out in our licence.

The Submitting Author accepts and understands that any supply made under these terms is made by BMJ to the Submitting Author unless you are acting as an employee on behalf of your employer or a postgraduate student of an affiliated institution which is paying any applicable article publishing charge ("APC") for Open Access articles. Where the Submitting Author wishes to make the Work available on an Open Access basis (and intends to pay the relevant APC), the terms of reuse of such Open Access shall be governed by a Creative Commons licence – details of these licences and which Creative Commons licence will apply to this Work are set out in our licence referred to above.

Other than as permitted in any relevant BMJ Author's Self Archiving Policies, I confirm this Work has not been accepted for publication elsewhere, is not being considered for publication elsewhere and does not duplicate material already published. I confirm all authors consent to publication of this Work and authorise the granting of this licence.

For peer review only

Surgical care in District Hospitals in sub-Saharan Africa: a Scoping Review

Authors:

Zineb Bentounsi, corresponding author, Botnar Research Centre, NDORMS, University of Oxford, Old Road, OX3 7LD, Oxford, UK. 07727064357, zineb.bentounsi@ndorms.ox.ac.uk

Sharaf Sheik Ali, sharaf554@gmail.com, Botnar Research Centre, NDORMS, University of Oxford, UK.

Grace Drury, grace.drury@ndorms.ox.ac.uk Botnar Research Centre, NDORMS, University of Oxford, UK.

Chris Lavy, chris.lavy@ndorms.ox.ac.uk Botnar Research Centre, NDORMS, University of Oxford, UK.

Keywords:

Global Surgery, Surgery, District Hospitals, Africa

Word count: 2961

For peer review only

Abstract:

Objective. To provide a general overview of the reported current surgical capacity and delivery in order to advance current knowledge and suggest targets for further development and research within the region of SSA

Design: Scoping Review

Setting: District hospitals in sub-Saharan Africa

Data sources: PubMed and Ovid EMBASE from January 2000 to December 2019

Study selection: Studies were included if they contained information about types of surgical procedures performed, number of operations per year, types of anaesthesia delivered, cadres of surgical/anaesthesia providers and/or patients' outcomes.

Results: The 52 articles included in analysis provided information about 16 countries. District hospitals were a group of diverse institutions ranging from 21 to 371 beds. The 3 most frequently reported procedures were caesarean section, laparotomy and hernia repair but a wide range of orthopaedics, plastic surgery and neurosurgery procedures were also mentioned. The number of operations performed per year per district hospital ranged from 239 to 5233. The most mentioned anaesthesia providers were non-physician clinicians trained in anaesthesia. They deliver mainly general and spinal anaesthesia. Depending on countries, articles referred to different surgical care providers: specialist surgeons, medical officers and non-physician clinicians. 15 articles reported peri-operative complications among which surgical site infection was the most frequent. 15 articles reported perioperative deaths of which the leading causes were sepsis, haemorrhage and anaesthesia complications.

Conclusion: District hospitals play a significant role in sub-Saharan Africa, providing both emergency and elective surgery. Most procedures are done under general or spinal anaesthesia, often administered by non-physician clinicians. Depending on countries, surgical care may be provided by medical officers, specialist surgeons and/or non-physician clinicians. Research on safety, quality and volume of surgical and anaesthesia care in this setting is scarce and more attention to these questions is required.

Article Summary:

Strengths and limitations:

- This is the first review to systematically assess the literature for the scope, volume and quality of surgery in district hospitals in sub-Saharan Africa.
- Article selection and data extraction were conducted independently by two authors
- Our search was conducted in English, French and Portuguese.
- This study is prone to publication bias as it is based on information present in published literature.
- There was a lot of heterogeneity in how articles reported surgical volume and quality.

For peer review only - http://bmjopen.bmj.com/site/about/guidelines.xhtml

Introduction:

The burden of surgical disease in Sub-Saharan Africa (SSA) is estimated at 38 disability adjusted life years (DALYs) per 1,000 population. District hospitals bear a large burden of this surgical care delivery.(1) According to The World Health Organization (WHO), district hospitals are defined as the first level of healthcare facilities that provide in-patient surgery and anaesthesia.(2) These hospitals which mainly serve rural populations, face challenges in terms of workforce, infrastructure and other essential resources. In 2010, it was estimated that there was only 1 surgeon for 25 million people in rural SSA(3) and although training programs in the region are increasing the number of surgeons, the main burden in several countries remains on non-physician clinicians to manage surgical cases.(4)

District hospitals offer a unique opportunity to improve access to surgical care in SSA as they are the first point of access to surgical care for patients and relieve referral hospitals from undue burden, allowing them to focus on complicated cases. Delivering timely surgical care in district hospitals has been shown to be highly cost effective(5) which should provide encouragement for governments and global health programs to invest in them. The Lancet Commission on Global Surgery,(6) which defines scalable solutions for the provision of quality surgical and anaesthesia care for all, reinforces this emphasis on district hospitals and echoes other authors' calls to consider district hospitals as a central component in strengthening surgical care in SSA.(7)(8)

We considered a systematic review of district hospital surgery, however in view of the limited number of existing studies in this field and the wide range of methodology used, we chose to change the review methodology and undertake a scoping review of surgical care delivery in district hospitals in SSA, aiming to provide a general overview of the reported current surgical capacity and delivery in order to advance current knowledge and suggest targets for further development and research within the region of SSA.

Methods:

This scoping review follows the five step methodology designed by Arksey and O'Malley(9) that offers a rigorous framework for reporting findings. We have also considered the recommendations of Levac, Colquhoun and O'Brien(10) that supplement this framework.

1. Identifying the research question

To guide our search of the literature, we defined the following questions:

Primary outcome:
In district hospitals in sub Saharan Africa, what types of surgical procedures are provided?

Secondary outcomes:
In district hospitals in sub-Saharan Africa:
- How many surgical procedures are performed per year?
- What type of surgical procedures are performed?
- Are there any measures of quality, safety or outcomes of surgery?
- Who provides anaesthesia and/or surgery?

For peer review only - http://bmjopen.bmj.com/site/about/guidelines.xhtml

❖ What type of anaesthesia is delivered?

2. Identifying relevant studies

We searched PubMed and Ovid EMBASE on 18/12/19 to retrieve literature about surgery in district hospitals in sub-Saharan Africa. The search strategy combined the exploded thesaurus terms “surgery”, “surgical procedures, operative” and “specialties, surgical” with the exploded thesaurus terms “hospitals, district” and “Africa south of the Sahara”. We searched references in English, French and Portuguese. We limited the search to references published after January 1st, 2000 in order to review more recent studies and gain a clearer understanding of more recent developments in SSA. We also searched Google Scholar and the Cochrane Library with the same search terms, but they did not contribute any relevant new references.

3. Study selection

The most important inclusion criteria was the setting: a district-level hospital in sub-Saharan Africa. We included articles that provided information about either type of surgical procedures performed, number of operations per year, types of anaesthesia delivered, cadres of surgical/anaesthesia providers and/or patient outcomes. We excluded articles if the setting was unclear or was not a district hospital, and if they were strictly about costs or burden of disease. We excluded articles if the full text could not be retrieved. We included all surgical procedures except those typically done in outpatient departments, such as dental and ophthalmic procedures.

Z.B. and G.L. independently screened the abstracts using the web application Rayyan,(11) resolving any conflicts by dialogue between Z.B, G.L and C.L. The process of selection followed the Preferred Reporting of Items for Systematic Reviews and Meta-Analyses (PRISMA) Statement.

4. Charting the data

Z.B. developed the data charting form after consultation with all other authors. The form was piloted and then updated iteratively during the extraction process (see supplementary file). The form contained 20 fields of data entry covering information about articles (e.g. name of first author, year of publication, research method) and information about district hospitals, surgical and anaesthesia providers, surgical procedures as well as other relevant information. Z.B. and S.S. extracted the data independently.

5. Collating, summarizing and reporting the results

S.S. produced descriptive numerical summaries of surgical volume, types of surgical procedures and types of anaesthesia using Excel. Z.B. performed a thematic analysis in NVivo(12) with surgical providers, quality of care and surgical outcomes as themes. We reported results to answer the primary and secondary outcomes of the review. All authors discussed and reported implications for research and policy.

For peer review only - http://bmjopen.bmj.com/site/about/guidelines.xhtml

Results:

In this section, we first present characteristics of the included articles and an analysis of the district hospitals. Thereafter, we present our findings on the scope and volume of surgery, types of anaesthesia, surgical and anaesthesia providers, and finally safety and quality of care.

1. Study Selection

We identified 302 articles and after removing duplicates, 267 articles remained. We screened the titles and abstracts, and, using our criteria noted above, excluded 167 articles. Then, we assessed full texts of the remaining 100 articles and excluded 48. Therefore, we included 52 articles for analysis. A PRISMA diagram is presented in Figure 1.

Figure 1: PRISMA diagram illustrating the selection process

2. Study Characteristics

Thirty three per cent of articles were cross-sectional surveys, 17% were prospective studies and 11% were based on retrospective review of hospital records (see Table 1). Most articles (93%) described the surgical care provided by staff based at the hospital, however, four articles reported external support from Non-Governmental Organisations (NGO) or visiting surgical teams (13–17)

Table 1: Summary of studies selected

First Author	Year of Publication	Study setting	Study Design	Objective
Abdullah(18)	2011	Ghana	Mixed methods	Assess surgical and obstetrical capacity
Cheelo(19)	2018	Zambia	Cross-sectional survey	Assess surgical capacity
Choo(20)	2013	Ghana	Cross-sectional survey	Appraise quality improvement activities
Choo(21)	2011	Ghana	Qualitative methods	Explore the training of medical officers providing surgery
Compaoré(22)	2014	Burkina Faso	Cross-sectional survey	Assess the readiness to provide caesarean sections

For peer review only - http://bmjopen.bmj.com/site/about/guidelines.xhtml

Damien(23)	2011	Ghana	Review of hospital records	Evaluate surgical volume
De Brouwere(24)	2009	Senegal	Mixed methods	Evaluate the task shifting policy
Dossche(14)	2014	Burkina Faso	Case series	Analyse technical problems and post-operative complications
Dresser(25)	2017	Uganda	Review of hospital records	Evaluate the management of surgical patients by non-physician emergency clinicians
Fehr(26)	2006	Tanzania	Prospective cohort study	Identify risk factors for surgical site infection
Fehr(27)	2006	Tanzania	Prospective cohort study	Evaluate peri-operative antimicrobial prohylaxis
Fenton(28)	2003	Malawi	Prospective cohort study	Identify risk factors associated with maternal death after caesarean section
Gajewski(29)	2017	Malawi	Baseline and follow up patients' surveys	Compare surgical outcomes between central and district hospitals
Gajewski(30)	2019	Malawi, Tanzania, Zambia	Mixed-methods	Investigate of anaesthesia capacity
Gajewski(31)	2018	Malawi	Qualitative methods	Explore providers perspectives on obstacles to surgery

For peer review only - http://bmjopen.bmj.com/site/about/guidelines.xhtml

Gajewski(32)	2019	Malawi	Randomised Control Trial	Evaluate surgical training programme for clinical officers
Galukande(4)	2010	Uganda, Tanzania, Mozambique	Review of hospital records	Assess the scope of surgery provided
Grimes(33)	2012	Sub-Saharan Africa	Systematic Review	Identify the met and unmet needs of surgical disease
Gyedu(34)	2018	Ghana	Cross-sectional survey	Evaluate the operation rate in Ghana
Hall(35)	2015	Ghana	Review of hospital records	Evaluate the impact of a newly placed obstetrician on health outcomes
Harfouche(36)	2015	Malawi	Prospective cohort study	Assess the quality of emergency caesarean sections
Henry(37)	2015	Sub-Saharan Africa	Consensus meeting+ interviews	Identify a package of essential surgical operations
Henry(38)	2015	Malawi	Cross-sectional survey	Assess surgical capacity
Kenfack(39)	2012	Cameroon	Case series	Describe the management of ectopic pregnancy
Koigi-Kamau(40)	2005	Kenya	Prospective cohort study	Describe the incidence of post-caesarean wound infection
Lavy(41)	2007	Malawi	Review of hospital records	Estimate surgical volume

For peer review only - http://bmjopen.bmj.com/site/about/guidelines.xhtml

LeBrun(42)	2014	Ethiopia, Liberia, Rwanda, Uganda	Cross-sectional survey	Assess surgical capacity
Lofgren(43)	2015	Uganda	Cross-sectional survey	Investigate indications, interventions and peri-operative mortality
Lonnée (40)	2018	Zimbabwe	Cross-sectional survey	Describe anaesthesia care for caesarean sections
Luo(44)	2018	Ghana	Qualitative methods	Explore the impact of a newly placed obstetrician in a district hospital
McCord(45)	2012	Ghana	Commentary	Describe surgical care in district hospitals in Ghana
McCord(46)	2009	Tanzania	Review of hospital records	Evaluate the quality of emergency obstetric surgery done by assistant medical officers
Mehtsun(47)	2012	Ghana	Observational study	Assess surgical workload of medical officers
Mpirimbanyi(17)	2017	Rwanda	Cross-sectional survey	Describe the spectrum, management and patient outcomes of emergency surgery
Muhirwa(15)	2016	Rwanda	Cohort study	Describe the presentation, management and outcomes

For peer review only - http://bmjopen.bmj.com/site/about/guidelines.xhtml

				of surgical patients
Ngaroua(48)	2017	Cameroon	Case series	Describe the management of urethral stenosis
Nkurunziza(16)	2019	Rwanda	Cross-sectional survey	Estimate the prevalence of surgical site infection after caesarean section
Nordberg(49)	2002	Kenya	Cross-sectional survey	Estimate of the surgical volume
Notrica(50)	2011	Rwanda	Cross-sectional survey	Assess surgical and anaesthesia infrastructure
Ottaway(51)	2018	Namibia	Prospective Observational Study	Determine the prevalence of anaesthetic adverse events
Ouédraogo(52)	2015	Burkina Faso	Prospective Observational Study	Describe task shifting in obstetric care
Ouédraogo(53)	2015	Burkina Faso	Case series	Describe prognosis of patients undergoing caesarean sections
Petroze(54)	2012	Rwanda	Cross-sectional survey	Evaluate the Ratio of caesarean sections to total procedures as a marker of trauma capacity
Petroze(55)	2012	Rwanda	Mixed methods	Assess emergency and essential surgical capacity

For peer review only - http://bmjopen.bmj.com/site/about/guidelines.xhtml

Ramos(13)	2013	Ethiopia	Case series	Describe thyroid surgery cases
Rutgers(56)	2008	Zimbabwe	Cross-sectional survey	Describe the mortality associated with caesarean sections
Sani(57)	2009	Niger	Cross-sectional survey	Assess surgical care provided by general practitioners
Sherman(58)	2011	Liberia	Cross-sectional survey	Assess emergency and essential surgical care
Smiley(59)	2019	Ghana	Surveys + process mapping	Evaluate patients and staff perception of quality of care + Analyse peri-operative process
Stewart(60)	2015	Ghana	Cross-sectional survey	Assess trauma capacity
Van Amelsfoort(61)	2010	Malawi	Mixed methods	Describe the training and experiences of clinical officers
Van den Akker(62)	2009	Malawi	Mixed methods	Evaluate the impact of audits in reducing the incidence of uterine ruptures

This review covered 16 countries with a few articles presenting data from multiple countries(4,30,42) and others giving a regional perspective.(33,37) Ghana, Malawi and Rwanda were the three countries most represented in the articles selected, partly because of research projects in collaboration with researchers from High Income Countries (HIC) (Table 2)

For peer review only - http://bmjopen.bmj.com/site/about/guidelines.xhtml

Table 2: Distribution of articles per country

Country	Number of articles
Burkina Faso	3
Cameroon	2
Ethiopia	2
Ghana	11
Kenya	2
Liberia	2
Malawi	10
Mozambique	1
Namibia	1
Niger	1
Rwanda	7
Senegal	1
Sub Saharan Africa (regional perspective)	2
Tanzania	5
Uganda	4
Zambia	2
Zimbabwe	2

3. Characteristics of district hospitals:

District hospitals described in this review included a variety of institutions (see Table 3). In our review, the two smallest DH had 21 beds, reported in Liberia and Ghana by Sherman et al(58) and Choo et al(21) respectively. The biggest DH had 371 beds, reported by Fehr et al in Tanzania.(26) District hospitals served rural or urban populations, with some serving both groups. Although most were owned by the government, mission linked district hospitals also featured in several papers. On some occasions, hospitals received support from visiting surgeons or NGOs, e.g. in Rwanda, government owned district hospitals received support from Partners in Health.(15)

Table 3: Distribution of articles per size of district hospitals

Size of district hospitals (in number of beds)	Number of articles
<20	0
20-50	2
51-100	8
101-300	9
>300	2
Not specified	33

4. Scope of surgical operations

The majority of articles mentioned obstetrics/gynaecology and general surgery operations (Table 4). Indeed, the three most frequently reported procedures were caesarean section, laparotomy and hernia repair. However, a wide range of other surgical procedures were mentioned across the literature. These were mainly in orthopaedics, plastic surgery or neurosurgery and may have involved visiting specialists (Table 4). District hospitals reported in the literature handled both emergency procedures (such as caesarean sections,

For peer review only - http://bmjopen.bmj.com/site/about/guidelines.xhtml

appendicectomies and treatment of burns); and elective procedures (such as hernia repairs) and some even more complex procedures such as direct vision internal urethrotomy(48) and hip replacement.(14) Despite the high prevalence of traumatic brain injury in sub Saharan Africa(63) and the fact that cranial burr holes are included in the WHO book "Surgical Care at the District Hospital",(64) only three articles mentioned burr holes.

Table 4: Distribution of articles per surgical operations

Surgical Procedures	Number of articles
General Surgery	
Hernia Repair	26
Laparotomy	21
Appendicectomy	10
Abscess	7
Hydrocele repair	7
Thyroid surgery	5
Colectomy	4
Chest tube insertion	4
Prostatectomy	4
Cricothyroidotomy	3
Colostomy	2
Lipoma	2
Tracheostomy	2
Fibroma	1
Urethrotomy	1
Vascular repair	1
Bladder stone surgery	1
Obstetrics and Gynaecology	
Caesarean section	30
Salpingectomy (ectopic pregnancy)	10
Hysterectomy	9
Evacuation of Retained Products of Conception	7
Tubal Ligation	5
Fistula Repair	3
Cervical tear repair	2
Breast surgery	1
Orthopaedics	
Amputation	10
Open Fracture repair	8
Closed Fracture repair	5
Arthrotomy	3

For peer review only - http://bmjopen.bmj.com/site/about/guidelines.xhtml

Osteotomy	2
Total Hip replacement	1
Bipolar Hemiarthroplasty	1
Neurosurgery	
Major neurosurgery	1
Burr holes	3
Plastics	
Skin grafting	7
Debridement	5
Burn	4
Cleft lip/palate surgery	3
Contracture release	2
Escharotomy	1

5. Surgical volume

Only 13 articles provided enough data to be able to estimate the number of operations per year per district hospital performed. In some cases, there was not enough information to discriminate between major and minor operations, therefore the choice was made to present all operations combined. Using a simple formula; the number of operations, period of years and number of district hospitals, an average of the number of operations per district hospital per year was calculated (Table 5). Galukande et al(4) and LeBrun et al(42) contributed several reports on different district hospitals while Van Amelsfoort et al(61) contributed two reports on the same district hospital during two different time periods.

As seen in tables 5 and 6, the surgical volume of district hospitals varied from circa 200 to circa 5000 operations per year, with the majority of hospitals performing between 1001 and 5000 operations per year. Clear limitations to the use of these data is the sample size and selection bias. Of the 13 studies, only six analysed more than five district hospitals in a given area. These data, therefore, cannot solely be used as a general indicator for the productivity of district hospitals in the countries represented.

Table 5: Surgical volume (all reported cases) in number of operations per year per district hospital

First author	Study setting	Total number of operations	Years	Number of district hospitals	Estimated number of operations per district hospital per year *(to the nearest unit)*
Abdullah~ (18)	Ghana	-	1	10	774
Damien (23)	Ghana	1,391	5	1	278
Galukande	Tanzania	980	1	1	980

For peer review only - http://bmjopen.bmj.com/site/about/guidelines.xhtml

For peer review only

Galukande	Tanzania	2,045	1	1	2,045
Galukande	Mozambique	601	1	1	601
Galukande	Mozambique	256	1	1	256
Galukande	Uganda	1,484	1	1	1,484
Galukande	Uganda	248	1	1	248
Galukande	Uganda	239	12	1	239
Galukande	Uganda	1,835	1	1	1,835
Gyedu	Ghana	143,750	1	48	2,995
Lofgren $	Uganda	-	-	2	5,018
Lavy (41)	Malawi	25,053	1	21	1,193
LeBrun*	Uganda	-	1	1	1,296
LeBrun*	Rwanda	-	1	1	2,052
LeBrun*	Liberia	-	1	1	696
LeBrun*	Ethiopia	-	1	1	538
Nordberg~	Kenya	-	1	5	5,233
Notrica*	Rwanda	-	-	-	2,052
Ottaway	Namibia	737	0.58	4	316
Petroze	Rwanda	71,432	1	41	1,742
Sani	Niger	544	1	3	181
Van Amelsfoort	Malawi	19,644	1	17	1,156
Van Amelsfoort	Malawi	18,524	1	17	1,090

* Reported in the article as average number per year per district hospital
~ The article reported numbers for both major and minor operations and we calculated the total number of procedures.
$ This article reported that the number of major operations was estimated over a 4 month period while the number of minor operations was estimated over a 3 month period.

Table 6: Number of publications reporting the number of operations per year per district hospital

Number of operations on average per district hospital over 1 year	Number of publications
<100	0
100-500	6
501-1000	5
1001-5000	11
>5000	2

6. Types of anaesthesia

Out of 52 articles, 16 contained information about the type of anaesthesia used in the district hospitals studied. General anaesthesia was mentioned by 15 articles(13,15,17,18,22,23,29–

For peer review only - http://bmjopen.bmj.com/site/about/guidelines.xhtml

37) and spinal anaesthesia by 10 articles(14,17,21,28,43,51–53,58,65). Local anaesthesia and regional blocks were mentioned by 4 articles(15,17,43,58).

7. Surgical and anaesthesia providers

In each country, different cadres of healthcare professionals are responsible for providing surgical care in district hospitals (Table 7). In Ethiopia, Ramos et al reported the presence of a specialist surgeon.(13) In Ghana(18,21,34,35,44,45,47,59), Kenya(49), Rwanda(15–17,50,54,55) and Zimbabwe(56) medical officers worked alongside specialist surgeons or obstetrician/gynaecologists to provide surgical care. In Namibia(51) and Niger(57), the presence of medical officers as sole surgical providers was reported. Non-physician clinicians provided surgical care in Burkina Faso,(14,22,52) Liberia,(58) Malawi,(28,29,31,32,38) Senegal,(24) Tanzania,(30,46) Uganda(25,43) and Zambia.(19) In our review, there was no clear majority in terms of the number of articles mentioning each cadre.

Similarly, different cadres performed anaesthesia in different countries.
Articles reported the presence of a physician anaesthetist in district hospitals in Burkina Faso(14) and Ghana.(59) Medical officers providing anaesthesia on their own were reported in Namibia.(51) Otherwise, the most mentioned anaesthesia providers are non-physician clinicians trained in anaesthesia (Table 8). Their presence is reported in Burkina Faso,(22) Ethiopia,(13) Ghana,(21) Liberia,(58) Malawi,(28)(20) Rwanda,(50)(55) Senegal,(24) Tanzania,(30) Uganda,(43) Zambia(19) and Zimbabwe.(56)(65) There are also untrained non-physician clinicians providing anaesthesia in Liberia,(58) Malawi,(28)(20) Rwanda,(50)(55) Zimbabwe(56)(65) alongside trained personnel.

Table 7: Number of publications reporting surgical providers

Surgical Providers	Number of Publications
Specialist Surgeon/Obstetrician	5
Specialist Surgeon/Obstetrician & Medical Officers	9
Medical Officers	8
Medical Officers & Non-Physician Clinicians	4
Specialist Surgeon/Obstetrician & Non-Physician Clinicians	1
Non-Physician Clinicians	5
Specialists & Medical Officers & Non-Physician Clinicians	8

Table 8: Number of publications reporting anaesthesia providers

Anaesthesia Providers	Number of Publications
Physician Anaesthetists	2
Medical Officers	1
Medical Officer & Non-Physician Clinicians	1
Non-Physician Clinicians (trained)	10

For peer review only - http://bmjopen.bmj.com/site/about/guidelines.xhtml

Non-Physician Clinicians (trained & untrained)	6

8. Quality of care and surgical outcomes

8.1. Quality of care

Of all articles in our review, only nine articles evaluated the quality of care as part of their study. Choo et al surveyed 10 district hospitals in Ghana about quality improvement activities.(20) None of the hospitals surveyed reported an organised quality improvement programme (although this does not mean it was absent), and the only quality improvement activity related to surgery was found in one hospital and related to sterilisation of instruments.(20) Other authors echoed these findings by reporting a lack of clinical guidelines for surgery, anaesthesia and labour management(22,40,58) and a lack of staff trained in using them when they were available.(22)

To evaluate the quality of caesarean sections, Harfouche et al measured the "decision to incision time" in a district hospital in Malawi and found that it reached "1.69 hours per caesarean section, far exceeding the recommended 30 minutes".(36) They explained their findings by a "lack of operating theatre space and high patient volume" but also by an inefficient system to transfer patients to the operating theatre.(36)

Another major challenge to quality care in district hospitals in sub-Saharan Africa is the shortage of trained surgical and anaesthesia staff. Stewart et al(60) explained that shortage in staff resulted in unavailability of service during nights and weekends. Choo et al found that medical officers in Ghana had limited exposure to surgery during their training.(21) Harfouche et al reported that while clinical officers performed most caesarean sections at district level in Malawi, they were not sufficiently trained in performing emergency hysterectomies.(36) Positive outcomes were observed in cases where trained staff were present: Hall et al reported an "association between the presence of OBGYNs in district hospitals in Ghana and an increase in evidence-based maternal care practices, as well as moderate improvement in maternal mortality and stillbirth rates".(35)

Some authors suggested interventions to improve the quality of care. Van den Akker et al suggested audit as a "simple intervention requiring little technology" that is able to improve quality of care.(62) They reported a decrease in 68% of uterine ruptures in a district hospital in Malawi after implementing recommendations inferred from an audit. Recommendations included training of staff, better documentation and involvement of hospital management.(62) Other authors insisted on the need for monitoring safety and quality of care in district hospitals as a first step toward improving quality.(20)(43)

However, Smiley et al reported care of exceptional quality in a "63 bed district hospital" named the "Volta River Authority Hospital" in Ghana(59). In their study they surveyed staff and patients about their perception of quality of care and conducted process mapping of surgical and obstetric care. They found that "over 80% of employees across a variety of perioperative roles held a positive view of teamwork and safety climate within the institution" and "surgical patients who were surveyed gave similarly positive indications of overall satisfaction".(59) The perception of staff and patients was complemented by the observations of researchers during the process mapping. The WHO safety checklist was used

For peer review only - http://bmjopen.bmj.com/site/about/guidelines.xhtml

in all operations witnessed and the authors reported the existence of a hospital wide morbidity and mortality conference as well as a safety training for new staff.(59)

8.2. Surgical outcomes

The most commonly used indicators of outcome and quality were peri-operative complications and peri-operative mortality. 15 articles reported information about peri-operative complications,(14–17,26–29,32,36,39,40,51,53,62) the most reported being surgical site infection. 15 articles included information about peri-operative mortality;(10-12,15,16,18,20,22,23,26,32,36,41,49,50) the most reported causes of death were sepsis, haemorrhage and anaesthesia complications.

In surgical obstetric care, the most reported complications were ruptured uterus, post-partum haemorrhage and wound dehiscence.(28,36,62) The most reported cause of maternal maternity was ruptured uterus.(15,18,20,23,26,30,32,49)

Discussion:

Although it varies by country, our review found that district hospitals provide mainly emergency obstetric and general surgery and, in some cases, elective surgery covering a wide range of surgical specialties. The literature reports that, most often, surgical procedures are done under general or spinal anaesthesia.

Many of the procedures performed are by trained non-physician clinicians and medical officers. There is a paucity of data regarding quality of surgical care, surgical volume and peri-operative morbidity and mortality. However, the most reported complications are infections and post-partum haemorrhage.

This is the first review to systematically assess the literature for the scope, volume and quality of surgery in district hospitals in sub-Saharan Africa, to our knowledge. To minimise subjectivity, two authors conducted the article selection and data extraction. To mitigate a language bias, we did not limit our search to English, but included French and Portuguese.

However, this study has some limitations. First, we could have missed relevant literature that did not specifically use the terminology ‘district hospital’ but instead used other terms such as ‘rural hospital’. Second, our study is prone to some publication bias as it is based only on information present in published literature. It is unlikely that institutions that are not actively auditing activity would present it for publication, and even those district hospitals that do collect information of relevance to this study are unlikely to submit it for publication. Indeed, these institutions face several barriers to publish, such as the cost of submissions to journals or overwhelming clinical demands reduce the time available for academic work. Furthermore, there is usually a cost of running trials/data collection and, if the research is published, there is often a pay wall burden resulting in a limited distribution of results for the readership in LMICs. Finally, our results are limited by the heterogeneity in how articles reported indicators such as personnel training level, surgical volume in terms of minor and major operations, complications, outcomes, and peri-operative mortality. Some articles used surgical indicators but these were usually non-standard, and the core surgical indicators (geographical access to surgical care within 2 hours, surgical providers density, total

For peer review only - http://bmjopen.bmj.com/site/about/guidelines.xhtml

operative volume, in-hospital postoperative mortality, and impoverishing and catastrophic cost burden) recommended by the Lancet Commission on Global Surgery(6) were not routinely used.

Our study is a snapshot of two decades of published surgical activity in district hospitals. We had aimed to compare it with other similar studies, but we found none that were appropriate, either in terms of similar methodology, scope or research question. We suggest that there is a challenge and an imperative for district hospitals to collect information on key indicators so that standards can be measured, and quality improvement encouraged. Our preference is to start with the agreed indicators recommended by the Lancet Commission on Global Surgery.(6)

Based on the findings of this study we suggest that the key policy recommendation for Ministries of Health in sub-Saharan Africa is to support district hospitals, through policy changes and resources, in standardizing and improving quality of recording of surgical activity, and quality improvement practice for district-level surgical care the region.

Conclusion:

District hospitals play a significant role in surgical care in sub-Saharan African countries.

This scoping review has provided a broad but incomplete data set on the current provision of surgical care in district hospitals in sub-Saharan Africa as reported in published studies. Based on the findings of this study, we recommend to researchers to conduct more primary surgical systems research in district hospitals to report on surgical activity, using core indicators suggested by the Lancet Commission on Global Surgery that will improve comparability.

Authors Contribution:

Zineb Bentounsi, MD, MSc: investigation, data curation, methodology, formal analysis, writing-original draft, writing-review and editing

Sharaf Sheik Ali, MRCS (ENT), MBBS, BSc: investigation, formal analysis, writing-original draft, writing- review and editing

Grace Drury, MA: project administration, investigation, writing-review and editing

Chris Lavy, MBBS, MD: conceptualization, supervision, writing-review and editing

Data Sharing Statement: Data available upon request

Patient and public involvement: Patients and the public were not involved in the design or conduct of the study.

For peer review only - http://bmjopen.bmj.com/site/about/guidelines.xhtml

For peer review only

Conflict of Interest: Authors have no conflict of interest.

Funding Statement: This work was supported by SURG Africa through the European Union Horizon 2020 programme Grant Agreement no. 733391.

References:

1. Luboga S, Macfarlane SB, von Schreeb J, Kruk ME, Cherian MN, Bergström S, et al. Increasing access to surgical services in sub-saharan Africa: priorities for national and international agencies recommended by the Bellagio Essential Surgery Group. PLoS Med [Internet]. 2009/12/22. 2009 Dec;6(12):e1000200–e1000200. Available from: https://pubmed.ncbi.nlm.nih.gov/20027218
2. Mulligan J-A, Fox-Rushby JA, Adam T, Johns B, Mills A. Unit Costs of Health Care Inputs in Low and Middle Income Regions. 2003.
3. Pollock JD, Love TP, Steffes BC, Thompson DC, Mellinger J, Haisch C. Is it possible to train surgeons for rural Africa? A report of a successful international program. World J Surg. 2011 Mar 30;35(3):493–9.
4. Galukande M, von Schreeb J, Wladis A, Mbembati N, de Miranda H, Kruk ME, et al. Essential surgery at the district hospital: a retrospective descriptive analysis in three African countries. PLoS Med. 2010 Mar;7(3):1–10.
5. Roberts G, Roberts C, Jamieson A, Grimes C, Conn G, Bleichrodt R. Surgery and Obstetric Care are Highly Cost-Effective Interventions in a Sub-Saharan African District Hospital: A Three-Month Single-Institution Study of Surgical Costs and Outcomes. World J Surg. 2016;40:14–20.
6. Meara, J. G., Leather, A. J., Hagander, L., Alkire, B. C., Alonso, N., Ameh EA. Global Surgery 2030: evidence and solutions for achieving health, welfare, and economic development. Lancet. 2015;386(9993):569–624.
7. Rajbhandari R, McMahon DE, Rhatigan JJ, Farmer PE. The neglected hospital - The district hospital's central role in global health care delivery [Internet]. Vol. 382, New England Journal of Medicine. Massachussetts Medical Society; 2020 [cited 2020 Jun 3]. p. 397–400. Available from: http://www.nejm.org/doi/10.1056/NEJMp1911298
8. Grimes C, Lavy C. A plea for investment in district hospitals [Internet]. Vol. 376, The Lancet. Elsevier; 2010 [cited 2020 Jun 30]. p. 2073. Available from: http://www.thelancet.com/article/S0140673610622933/fulltext
9. Arksey H, O'Malley L. Scoping studies: towards a methodological framework. Int J Soc Res Methodol. 2005;8(1):19–32.
10. Levac D, Colquhoun H, O'Brien KK. Scoping studies: Advancing the methodology. Implement Sci. 2010;5(1):1–9.
11. Ouzzani M, Hammady H, Fedorowicz Z, Elmagarmid A. Rayyan-a web and mobile app for systematic reviews. Syst Rev [Internet]. 2016;5(1):210. Available from: http://dx.doi.org/10.1186/s13643-016-0384-4
12. QSR International. NVivo Qualitative Data Analysis Software- Version 12. 2018.
13. Ramos JM, Abate N, Reyes F, Belate W, Mohammed F, Gorgolas M. Thyroid surgery in a district hospital in Southern Ethiopia: experience from a rural center. 2013;37(7). Available from: http://www.ncbi.nlm.nih.gov/pubmed/23564217
14. Dossche L, Noyez JF, Ouedraogo W, Kalmogho E. Establishment of a hip replacement project in a district hospital in Burkina Faso: analysis of technical problems and peri-operative complications. Bone Joint J [Internet]. 2014;96(2):177–80. Available from: http://www.ncbi.nlm.nih.gov/pubmed/24493181

For peer review only - http://bmjopen.bmj.com/site/about/guidelines.xhtml

15. Muhirwa E, Habiyakare C, Hedt-Gauthier BL, Odhiambo J, Maine R, Gupta N, et al. Non-Obstetric Surgical Care at Three Rural District Hospitals in Rwanda: More Human Capacity and Surgical Equipment May Increase Operative Care. World J Surg. 2016;40(9):2109–16.
16. Nkurunziza T, Kateera F, Sonderman K, Gruendl M, Nihiwacu E, Ramadhan B, et al. Prevalence and predictors of surgical-site infection after caesarean section at a rural district hospital in Rwanda. Br J Surg [Internet]. 2019;106(2):e121–8. Available from: http://www.ncbi.nlm.nih.gov/pubmed/30620071
17. Mpirimbanyi C, Nyirimodoka A, Lin Y, Hedt-Gauthier BL, Odhiambo J, Nkurunziza T, et al. Emergency general surgery in Rwandan district hospitals: a cross-sectional study of spectrum, management, and patient outcomes. 2017;17(1):121.
18. Abdullah F, Choo S, Hesse AAJ, Abantanga F, Sory E, Osen H, et al. Assessment of surgical and obstetrical care at 10 district hospitals in Ghana using on-site interviews. J Surg Res [Internet]. 2011;171(2):461–6. Available from: http://www.ncbi.nlm.nih.gov/pubmed/20691981
19. Cheelo M, Brugha R, Bijlmakers L, Kachimba J, McCauley T, Gajewski J. Surgical Capacity at District Hospitals in Zambia: From 2012 to 2016. World J Surg. 2018 Nov 1;42(11):3508–13.
20. Choo S, Papandria D, Goldstein SD, Perry H, Hesse AAJ, Abatanga F, et al. Quality improvement activities for surgical services at district hospitals in developing countries and perceived barriers to quality improvement: findings from Ghana and the scientific literature. World J Surg [Internet]. 2013;37(11):2512–9. Available from: http://www.ncbi.nlm.nih.gov/pubmed/23897444
21. Choo S, Perry H, Hesse AAJ, Abantanga F, Sory E, Osen H, et al. Surgical training and experience of medical officers in Ghana's district hospitals. 2011;86(4). Available from: http://www.ncbi.nlm.nih.gov/pubmed/21346502
22. Compaoré GD, Sombié I, Ganaba R, Hounton S, Meda N, Brouwere V De, et al. Readiness of district and regional hospitals in Burkina Faso to provide caesarean section and blood transfusion services: a cross-sectional study. BMC Pregnancy Childbirth. 2014;14:158.
23. Damien P, Nabare C, Baiden F, Kwara E, Apanga S, Etego-Amengo S. How are surgical theatres in rural Africa utilized? A review of five years of services at a district hospital in Ghana. Trop Doct [Internet]. 2011;41(2):91–5. Available from: http://www.ncbi.nlm.nih.gov/pubmed/21421886
24. De Brouwere V, Dieng T, Diadhiou M, Witter S, Denerville E. Task shifting for emergency obstetric surgery in district hospitals in Senegal. Reprod Health Matters. 2009;17(33):32–44.
25. Dresser, Caleb; Periyanayagam, Usha; Dreifuss, Brad; Wangoda, Robert; Luyimbaazi, Julius; Bisanzo MGECCIGECC. Management and Outcomes of Acute Surgical Patients at a District Hospital in Uganda with Non-physician Emergency Clinicians. World J Surg [Internet]. 2017 [cited 2020 Apr 28]; Available from: https://rayyan.qcri.org/fulltexts/320248
26. Fehr J, Hatz C, Soka I, Kibatala P, Urassa H, Smith T, et al. Risk Factors for Surgical Site Infection in a Tanzanian District Hospital: A Challenge for the Traditional National Nosocomial Infections Surveillance System Index. Infect Control Hosp Epidemiol [Internet]. 2006;27(12):1401–4. Available from: http://ovidsp.ovid.com/ovidweb.cgi?T=JS&PAGE=reference&D=ovfth&NEWS=N&AN=00004848-200612000-00019
27. Fehr J, Hatz C, Soka I, Kibatala P, Urassa H, Battegay M, et al. Antimicrobial prophylaxis to prevent surgical site infections in a rural sub-Saharan hospital. Clin

For peer review only - http://bmjopen.bmj.com/site/about/guidelines.xhtml

Microbiol Infect [Internet]. 2006;12(12):1224–7. Available from: http://ovidsp.ovid.com/ovidweb.cgi?T=JS&PAGE=reference&D=ovfth&NEWS=N&AN=00125357-200612000-00012

28. Fenton PM, Whitty CJM, Reynolds F. Caesarean section in Malawi: prospective study of early maternal and perinatal mortality. BMJ. 2003;327(7415):587.

29. Gajewski J, Conroy R, Bijlmakers L, Mwapasa G, McCauley T, Borgstein E, et al. Quality of Surgery in Malawi: Comparison of Patient-Reported Outcomes After Hernia Surgery Between District and Central Hospitals. World J Surg. 2018;42(6):1610–6.

30. Gajewski J, Pittalis C, Lavy C, Borgstein E, Bijlmakers L, Mwapasa G, et al. Anesthesia Capacity of District-Level Hospitals in Malawi, Tanzania, and Zambia: A Mixed-Methods Study. Anesth Analg [Internet]. 2019 Apr 1 [cited 2020 Apr 28];130(4):845–53. Available from: http://ovidsp.ovid.com/ovidweb.cgi?T=JS&PAGE=reference&D=ovftu&NEWS=N&AN=00000539-900000000-96017

31. Gajewski J, Bijlmakers L, Mwapasa G, Borgstein E, Pittalis C, Brugha R. "I think we are going to leave these cases". Obstacles to surgery in rural Malawi: a qualitative study of provider perspectives. Trop Med Int Heal [Internet]. 2018 Oct 1 [cited 2020 Apr 21];23(10):1141–7. Available from: http://ovidsp.ovid.com/ovidweb.cgi?T=JS&PAGE=reference&D=ovftt&NEWS=N&AN=00060771-201810000-00013

32. Gajewski J, Borgstein E, Bijlmakers L, Mwapasa G, Aljohani Z, Pittalis C, et al. Evaluation of a surgical training programme for clinical officers in Malawi. BJS [Internet]. 2019 Jan 1 [cited 2020 Nov 18];106(2):e156–65. Available from: https://pubmed.ncbi.nlm.nih.gov/30620067/

33. Grimes CE, Law RSL, Borgstein ES, Mkandawire NC, Lavy CBD. Systematic review of met and unmet need of surgical disease in rural sub-Saharan Africa. World J Surg [Internet]. 2012;36(1):8–23. Available from: http://www.ncbi.nlm.nih.gov/pubmed/22057752

34. Gyedu A, Stewart B, Gaskill C, Boakye G, Appiah-Denkyira E, Donkor P, et al. Improving Benchmarks for Global Surgery: Nationwide Enumeration of Operations Performed in Ghana. Ann Surg. 2018;268(2):282–8.

35. Hall E, Sevugu J, Danso K, Adomako J, Peltzman T, Anderson FJ. A retrospective analysis of the impact of an obstetrician on delivery and care outcomes at four district hospitals in Ghana. Int J Gynaecol Obstet [Internet]. 2015;131(1):100–1. Available from: http://www.ncbi.nlm.nih.gov/pubmed/26143230

36. Harfouche M, Hosseinipour M, Kaliti S, Wilkinson J. Quality indicators and outcomes of emergency caesarean deliveries at a district-level maternity hospital. Afr J Reprod Health. 2015;19(3):61–7.

37. Henry JA, Bem C, Grimes C, Borgstein E, Mkandawire N, Thomas WEGG, et al. Essential surgery: the way forward. World J Surg. 2015;39(4):822–32.

38. Henry J, Frenkel E, Borgstein E, Mkandawire N, Goddia C. Surgical and anaesthetic capacity of hospitals in Malawi: key insights. Heal Policy Plan [Internet]. 2015;30(8):985–94. Available from: http://ovidsp.ovid.com/ovidweb.cgi?T=JS&PAGE=reference&D=ovftq&NEWS=N&AN=00003845-201510000-00005

39. Kenfack B, Noubom M, Bongoe A, Tsatedem FA, Ngono M, Tsague GN, et al. [Ectopic pregnancy in a semi-rural area in Africa: Epidemiological, clinical and therapeutic aspects about a series of 74 cases treated at the District Hospital of Sangmelima in southern Cameroon]. Pan Afr Med J. 2012;13:71.

For peer review only - http://bmjopen.bmj.com/site/about/guidelines.xhtml

40. Koigi-Kamau R, Kabare LW, Wanyoike-Gichuhi J. Incidence of wound infection after Caesarean delivery in a district hospital in Central Kenya. East Afr Med J. 2005;82(7):357–61.
41. Lavy C, Tindall A, Steinlechner C, Mkandawire N, Chimangeni S. Surgery in Malawi - a national survey of activity in rural and urban hospitals. Ann R Coll Surg Engl [Internet]. 2007;89(7):722–4. Available from: http://ovidsp.ovid.com/ovidweb.cgi?T=JS&PAGE=reference&D=ovfti&NEWS=N&AN=00000639-200710000-00025
42. LeBrun DG, Chackungal S, Chao TE, Knowlton LM, Linden AF, Notrica MR, et al. Prioritizing essential surgery and safe anesthesia for the Post-2015 Development Agenda: operative capacities of 78 district hospitals in 7 low- and middle-income countries. Surgery [Internet]. 2014;155(3):365–73. Available from: http://www.ncbi.nlm.nih.gov/pubmed/24439745
43. Löfgren J, Kadobera D, Forsberg BC, Mulowooza J, Wladis A, Nordin P. District-level surgery in Uganda: Indications, interventions and perioperative mortality. Surg (United States). 2015;158(1):7–16.
44. Luo EM, Opare-Ado HS, Adomako J, Danso KA, Peltzman T, Anderson FWJJ. Completing the Maternal Care Team: OB/GYN Expertise at Rural District Hospitals in Ghana, a Qualitative Study. 2018;22(7):1085–91. Available from: http://www.ncbi.nlm.nih.gov/pubmed/29520728
45. McCord C. Facilities for Surgery Exist in Ghana, but Skills Are Lacking: Comment on "Assessing the Surgical and Obstetrics-Gynecology Workload of Medical Officers". Arch Surg [Internet]. 2012;147(6):548–9. Available from: http://ovidsp.ovid.com/ovidweb.cgi?T=JS&PAGE=reference&D=ovftm&NEWS=N&AN=00000853-201206000-00013
46. McCord C, Mbaruku G, Pereira C, Nzabuhakwa C, Bergstrom S. The quality of emergency obstetrical surgery by assistant medical officers in Tanzanian district hospitals. Health Aff (Millwood) [Internet]. 28(5):w876-85. Available from: http://www.ncbi.nlm.nih.gov/pubmed/19661113
47. Mehtsun WT, Weatherspoon K, McElrath L, Chima A, Torsu VEK, Obeng ENB, et al. Assessing the surgical and obstetrics-gynecology workload of medical officers: findings from 10 district hospitals in Ghana. Arch Surg [Internet]. 2012;147(6):542–8. Available from: http://www.ncbi.nlm.nih.gov/pubmed/22786541
48. Ngaroua, Eloundou NJ, Djibrilla Y, Asmaou O, Mbo AJ. Aspects épidémiologiques, cliniques et prise en charge de sténose urétrale chez l'adulte dans un Hôpital de District de Ngaoundéré, Cameroun. Pan Afr Med J. 2017;26.
49. Nordberg E, Mwobobia I, Muniu E. Major and minor surgery output at district level in Kenya: review and issues in need of further research. Afr J Health Sci. 2002;9(1):17–25.
50. Notrica MR, Evans FM, Knowlton LM, Kelly McQueen KA. Rwandan surgical and anesthesia infrastructure: A survey of district hospitals. World J Surg. 2011;35(8):1770–80.
51. Ottaway AJ, Kabongo L. Prospective Observational Study of Intraoperative Anesthetic Events in District Hospitals in Namibia. 2018;126(2):632–8. Available from: http://www.ncbi.nlm.nih.gov/pubmed/29261548
52. Ouédraogo CM, Ouattara A, Ouédraogo A, Bikienga M, Lankoandé J. Amélioration de l'offre des soins obstétricaux par la délégation des tâches en milieu Africain Exemple de l'hôpital du district sanitaire de Bogodogo à Ouagadougou, Burkina Faso. Med Sante Trop. 2015;25(3):280–4.
53. Ouédraogo CM, Ouédraogo A, Ouattara A, Lankoandé J. [Cesarean deliveries in a

For peer review only - http://bmjopen.bmj.com/site/about/guidelines.xhtml

district hospital in Ouagadougou. Epidemiological, clinical, and prognostic study of 3381 cases]. Med Sante Trop [Internet]. 25(2):194–9. Available from: http://www.ncbi.nlm.nih.gov/pubmed/26081260

54. Petroze RT, Mehtsun W, Nzayisenga A, Ntakiyiruta G, Sawyer RG, Calland JF, et al. Ratio of cesarean sections to total procedures as a marker of district hospital trauma capacity. World J Surg. 2012;36(9):2074–9.
55. Petroze RT, Nzayisenga A, Rusanganwa V, Ntakiyiruta G, Calland JF. Comprehensive national analysis of emergency and essential surgical capacity in Rwanda. 2012;99(3). Available from: http://www.ncbi.nlm.nih.gov/pubmed/22237597
56. Rutgers RAK, Van Eygen L. Mortality related to caesarean section in rural Matebeleland North Province, Zimbabwe. Cent Afr J Med [Internet]. 54(5):24–7. Available from: http://www.ncbi.nlm.nih.gov/pubmed/21650076
57. Sani R, Nameoua B, Yahaya A, Hassane I, Adamou R, Hsia RY, et al. The impact of launching surgery at the district level in Niger. World J Surg. 2009;33(10):2063–8.
58. Sherman L, Clement P, Cherian M, Ndayimirije N, MD W-L, Noel L, et al. Implementing Liberia's Poverty Reduction Strategy: An Assessment of Emergency and Essential Surgical Care. Arch Surg [Internet]. 2011;146(1):35–9. Available from: http://ovidsp.ovid.com/ovidweb.cgi?T=JS&PAGE=reference&D=ovftl&NEWS=N&AN=00000853-201101000-00008
59. Smiley K, Ofori L, Spangler C, Acquaah-Arhin R, Deh D, Enos J, et al. Safety Culture and Perioperative Quality at the Volta River Authority Hospital in Akosombo, Ghana. 2019;43(1):16–23. Available from: http://www.ncbi.nlm.nih.gov/pubmed/30109388
60. Stewart BT, Quansah R, Gyedu A, Ankomah J, Donkor P, Mock C. Strategic Assessment of Trauma Care Capacity in Ghana. World J Surg. 2015;39(10):2428–40.
61. Van Amelsfoort JJC, Van Leeuwen PAM, Jiskoot P, Ratsma YEC. Surgery in Malawi – the training of clinical officers. Trop Doct. 2010;40(2):74–6.
62. van den Akker T, Mwagomba B, Irlam J, van Roosmalen J. Using audits to reduce the incidence of uterine rupture in a Malawian district hospital. Int J Gynaecol Obstet [Internet]. 2009;107(3):289–94. Available from: http://www.ncbi.nlm.nih.gov/pubmed/19846089
63. Hyder AA, Wunderlich CA, Puvanachandra P, Gururaj G, Kobusingye OC. The impact of traumatic brain injuries: A global perspective. Vol. 22, NeuroRehabilitation. IOS Press; 2007. p. 341–53.
64. World Health Organization. Surgical Care at the District Hospital. 2003.
65. Lonnee H, Madzimbamuto F, Erlandsen O, Vassenden A, Chikumba E, Dimba R, et al. Anesthesia for Cesarean Delivery: A Cross-Sectional Survey of Provincial, District, and Mission Hospitals in Zimbabwe. Anesth Analg [Internet]. 2018;126(6):2056–64. Available from: http://ovidsp.ovid.com/ovidweb.cgi?T=JS&PAGE=reference&D=ovftt&NEWS=N&AN=00000539-201806000-00040

For peer review only - http://bmjopen.bmj.com/site/about/guidelines.xhtml

PRISMA 2009 Flow Diagram

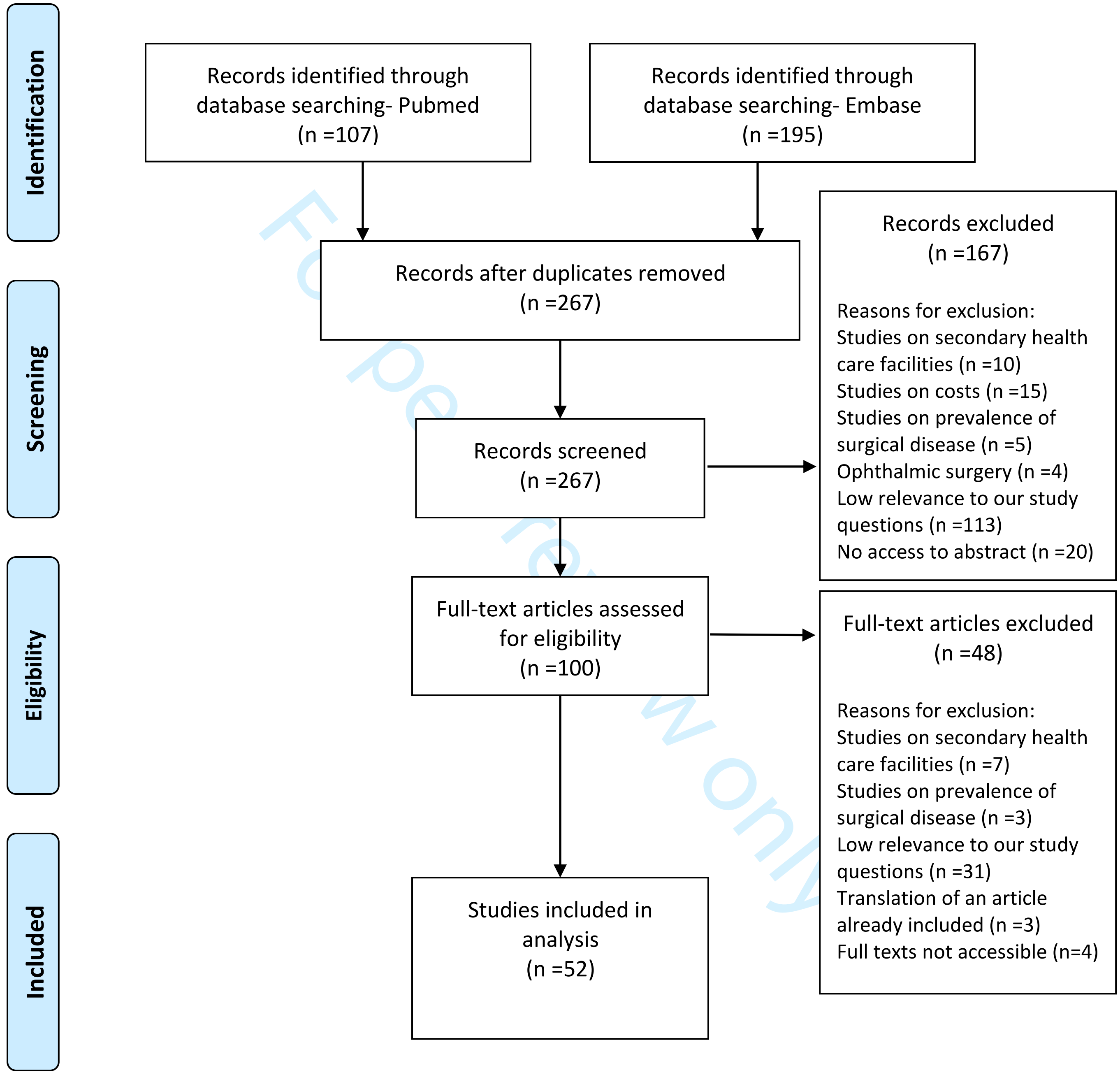

From: Moher D, Liberati A, Tetzlaff J, Altman DG, The PRISMA Group (2009). *P*referred *R*eporting *I*tems for *S*ystematic Reviews and *M*eta-*A*nalyses: The PRISMA Statement. PLoS Med 6(7): e1000097. doi:10.1371/journal.pmed1000097

For more information, visit www.prisma-statement.org.

For peer review only - http://bmjopen.bmj.com/site/about/guidelines.xhtml

Data extraction form for the scoping review

Surgical care in District Hospitals in sub Saharan Africa: a Scoping Review

* Required

1. First Author *

2. Year of publication *

3. Year of data collection (latest date to be chosen) *

4. Type of study/Research Method *

5. Country *

Check all that apply.

- [ ] Angola
- [ ] Burundi
- [ ] DRC
- [ ] Cameroon
- [ ] Central African Republic
- [ ] Chad
- [ ] Republic of the Congo
- [ ] Equatorial Guinea
- [ ] Gabon
- [ ] Kenya
- [ ] Nigeria
- [ ] Rwanda
- [ ] Sao Tome
- [ ] Tanzania
- [ ] Uganda
- [ ] Sudan
- [ ] South Sudan
- [ ] Djibouti
- [ ] Eritrea
- [ ] Ethiopia
- [ ] Somalia
- [ ] Botswana
- [ ] Comores
- [ ] Lesotho
- [ ] Madagascar
- [ ] Malawi
- [ ] Mauritius
- [ ] Mozambique
- [ ] Namibia
- [ ] Seychelles
- [ ] South Africa
- [ ] Swaziland
- [ ] ZAMBIA
- [ ] Zimbabwe
- [ ] Benin
- [ ] Mali
- [ ] Burkina Faso
- [ ] Cape Verde

For peer review only - http://bmjopen.bmj.com/site/about/guidelines.xhtml

- [ ] Ivory Cost
- [ ] Gambia
- [ ] Ghana
- [ ] Guinea
- [ ] Guinea-Bissau
- [ ] Liberia
- [ ] Mauritania
- [ ] Niger
- [ ] Senegal
- [ ] Sierra Leone
- [ ] Togo

Other: [ ] ____________________

6. Specialty *

Check all that apply.

- [ ] Obstetrics and Gynecology
- [ ] General Surgery
- [ ] Trauma and Orthopedics
- [ ] Head and Neck
- [ ] Urology
- [ ] Anesthesia
- [ ] Neurosurgery
- [ ] Paediatric surgery

Other: [ ] ____________________

7. Name of surgical procedures (please write all procedures mentioned) or write NA if none *

8. Surgical Provider *

Check all that apply.

- [ ] Specialist Surgeon (including OBGY)
- [ ] Medical Officer/GP
- [ ] Non Physician Clinician
- [ ] Nurse/Midwife
- [ ] Not specified

Other: [ ] ______________________

9. Type of Anaesthesia *

Check all that apply.

- [ ] General
- [ ] Regional Block
- [ ] Local
- [ ] Spinal
- [ ] Not specified

10. Anaesthesia Provider *

Check all that apply.

- [ ] Physician (anaesthesiologist)
- [ ] Non Physician- trained
- [ ] Non Physician- untrained
- [ ] Not specified
- [ ] Medical officer/Physician non anaesthesiologist

11. Number of operations per year/period (if available)

____

12. Catchment population (if available)

____

13. Equipment (if in a table, just write "table X" here)

14. Patients Outcomes (complications, survival...)

15. What do authors use as indicators for safety? (just list them)

16. What do authors use as indicators for surgical capacity?

17. Was the surgical care provided as a result of a temporary surgical mission or international partnership? *

Mark only one oval.

- Yes
- No
- It is unclear

18. Characteristics of the District Hospital(s) if given: number of bed, number of staff

19. Other important results

20. Recommendations made by authors

This content is neither created nor endorsed by Google.

Google Forms

Preferred Reporting Items for Systematic reviews and Meta-Analyses extension for Scoping Reviews (PRISMA-ScR) Checklist

SECTION	ITEM	PRISMA-ScR CHECKLIST ITEM	REPORTED ON PAGE #
**TITLE**			
Title	1	Identify the report as a scoping review.	Title
**ABSTRACT**			
Structured summary	2	Provide a structured summary that includes (as applicable): background, objectives, eligibility criteria, sources of evidence, charting methods, results, and conclusions that relate to the review questions and objectives.	Page 2
**INTRODUCTION**			
Rationale	3	Describe the rationale for the review in the context of what is already known. Explain why the review questions/objectives lend themselves to a scoping review approach.	Page 3
Objectives	4	Provide an explicit statement of the questions and objectives being addressed with reference to their key elements (e.g., population or participants, concepts, and context) or other relevant key elements used to conceptualize the review questions and/or objectives.	Page 3
**METHODS**			
Protocol and registration	5	Indicate whether a review protocol exists; state if and where it can be accessed (e.g., a Web address); and if available, provide registration information, including the registration number.	Prospero didn't accept to register a scoping review protocol
Eligibility criteria	6	Specify characteristics of the sources of evidence used as eligibility criteria (e.g., years considered, language, and publication status), and provide a rationale.	Page 4
Information sources*	7	Describe all information sources in the search (e.g., databases with dates of coverage and contact with authors to identify additional sources), as well as the date the most recent search was executed.	Page 4
Search	8	Present the full electronic search strategy for at least 1 database, including any limits used, such that it could be repeated.	Page 4 +supplementary material
Selection of sources of evidence†	9	State the process for selecting sources of evidence (i.e., screening and eligibility) included in the scoping review.	Page 4
Data charting process‡	10	Describe the methods of charting data from the included sources of evidence (e.g., calibrated forms or forms that have been tested by the team before their use, and whether data charting was done independently or in duplicate) and any processes for obtaining and confirming data from investigators.	Page 4
Data items	11	List and define all variables for which data were sought and any assumptions and simplifications made.	NA
Critical appraisal	12	If done, provide a rationale for conducting a critical	NA

For peer review only

For peer review only - http://bmjopen.bmj.com/site/about/guidelines.xhtml

SECTION	ITEM	PRISMA-ScR CHECKLIST ITEM	REPORTED ON PAGE #
of individual sources of evidence§		appraisal of included sources of evidence; describe the methods used and how this information was used in any data synthesis (if appropriate).	
Synthesis of results	13	Describe the methods of handling and summarizing the data that were charted.	Page 4
**RESULTS**			
Selection of sources of evidence	14	Give numbers of sources of evidence screened, assessed for eligibility, and included in the review, with reasons for exclusions at each stage, ideally using a flow diagram.	Page 5
Characteristics of sources of evidence	15	For each source of evidence, present characteristics for which data were charted and provide the citations.	Pages 5-17
Critical appraisal within sources of evidence	16	If done, present data on critical appraisal of included sources of evidence (see item 12).	NA
Results of individual sources of evidence	17	For each included source of evidence, present the relevant data that were charted that relate to the review questions and objectives.	Pages 5-17
Synthesis of results	18	Summarize and/or present the charting results as they relate to the review questions and objectives.	Page 5-17
**DISCUSSION**			
Summary of evidence	19	Summarize the main results (including an overview of concepts, themes, and types of evidence available), link to the review questions and objectives, and consider the relevance to key groups.	Page 17
Limitations	20	Discuss the limitations of the scoping review process.	Page 17
Conclusions	21	Provide a general interpretation of the results with respect to the review questions and objectives, as well as potential implications and/or next steps.	Page 18
**FUNDING**			
Funding	22	Describe sources of funding for the included sources of evidence, as well as sources of funding for the scoping review. Describe the role of the funders of the scoping review.	Page 19

JBI = Joanna Briggs Institute; PRISMA-ScR = Preferred Reporting Items for Systematic reviews and Meta-Analyses extension for Scoping Reviews.

* Where *sources of evidence* (see second footnote) are compiled from, such as bibliographic databases, social media platforms, and Web sites.

† A more inclusive/heterogeneous term used to account for the different types of evidence or data sources (e.g., quantitative and/or qualitative research, expert opinion, and policy documents) that may be eligible in a scoping review as opposed to only studies. This is not to be confused with *information sources* (see first footnote).

‡ The frameworks by Arksey and O'Malley (6) and Levac and colleagues (7) and the JBI guidance (4, 5) refer to the process of data extraction in a scoping review as data charting.

§ The process of systematically examining research evidence to assess its validity, results, and relevance before using it to inform a decision. This term is used for items 12 and 19 instead of "risk of bias" (which is more applicable to systematic reviews of interventions) to include and acknowledge the various sources of evidence that may be used in a scoping review (e.g., quantitative and/or qualitative research, expert opinion, and policy document).

For peer review only - http://bmjopen.bmj.com/site/about/guidelines.xhtml

From: Tricco AC, Lillie E, Zarin W, O'Brien KK, Colquhoun H, Levac D, et al. PRISMA Extension for Scoping Reviews (PRISMAScR): Checklist and Explanation. Ann Intern Med. 2018;169:467–473. doi: 10.7326/M18-0850.

For peer review only

BMJ Open

Surgical care in District Hospitals in sub-Saharan Africa: a Scoping Review

Journal:	*BMJ Open*
Manuscript ID	bmjopen-2020-042862.R3
Article Type:	Original research
Date Submitted by the Author:	10-Feb-2021

Complete List of Authors:	Bentounsi, Zineb; University of Oxford, Nuffield Department of Orthopaedics, Rheumatology and Musculoskeletal Sciences<br>Sheik-Ali, Sharaf; Oxford University Hospitals NHS Foundation Trust,<br>Drury, Grace; University of Oxford, Nuffield Department of Orthopaedics, Rheumatology and Musculoskeletal Sciences<br>Lavy, Chris; University of Oxford, Nuffiled Department of Orthopaedics, Rheumatology and Musculoskeletal Sciences

**Primary Subject Heading**:	Surgery
Secondary Subject Heading:	Anaesthesia
Keywords:	SURGERY, HEALTH SERVICES ADMINISTRATION & MANAGEMENT, ANAESTHETICS

SCHOLARONE™
Manuscripts

I, the Submitting Author has the right to grant and does grant on behalf of all authors of the Work (as defined in the below author licence), an exclusive licence and/or a non-exclusive licence for contributions from authors who are: i) UK Crown employees; ii) where BMJ has agreed a CC-BY licence shall apply, and/or iii) in accordance with the terms applicable for US Federal Government officers or employees acting as part of their official duties; on a worldwide, perpetual, irrevocable, royalty-free basis to BMJ Publishing Group Ltd ("BMJ") its licensees and where the relevant Journal is co-owned by BMJ to the co-owners of the Journal, to publish the Work in this journal and any other BMJ products and to exploit all rights, as set out in our licence.

The Submitting Author accepts and understands that any supply made under these terms is made by BMJ to the Submitting Author unless you are acting as an employee on behalf of your employer or a postgraduate student of an affiliated institution which is paying any applicable article publishing charge ("APC") for Open Access articles. Where the Submitting Author wishes to make the Work available on an Open Access basis (and intends to pay the relevant APC), the terms of reuse of such Open Access shall be governed by a Creative Commons licence – details of these licences and which Creative Commons licence will apply to this Work are set out in our licence referred to above.

Other than as permitted in any relevant BMJ Author's Self Archiving Policies, I confirm this Work has not been accepted for publication elsewhere, is not being considered for publication elsewhere and does not duplicate material already published. I confirm all authors consent to publication of this Work and authorise the granting of this licence.

For peer review only

Surgical care in District Hospitals in sub-Saharan Africa: a Scoping Review

Authors:

Zineb Bentounsi, corresponding author, Botnar Research Centre, Nuffield Department of Orthopaedics, Rheumatology and Musculoskeletal Sciences, University of Oxford, Old Road, OX3 7LD, Oxford, UK. 07727064357, zineb.bentounsi@ndorms.ox.ac.uk

Sharaf Sheik Ali, sharaf554@gmail.com, Botnar Research Centre, Nuffield Department of Orthopaedics, Rheumatology and Musculoskeletal Sciences, University of Oxford, UK.

Grace Drury, grace.drury@ndorms.ox.ac.uk Botnar Research Centre, Nuffield Department of Orthopaedics, Rheumatology and Musculoskeletal Sciences, University of Oxford, UK.

Chris Lavy, chris.lavy@ndorms.ox.ac.uk Botnar Research Centre, Nuffield Department of Orthopaedics, Rheumatology and Musculoskeletal Sciences, University of Oxford, UK.

Keywords:

Global Surgery, Surgery, District Hospitals, Africa

Word count: 3002

For peer review only

Abstract:

Objective. To provide a general overview of the reported current surgical capacity and delivery in order to advance current knowledge and suggest targets for further development and research within the region of sub-Saharan Africa.

Design: Scoping Review

Setting: District hospitals in sub-Saharan Africa

Data sources: PubMed and Ovid EMBASE from January 2000 to December 2019

Study selection: Studies were included if they contained information about types of surgical procedures performed, number of operations per year, types of anaesthesia delivered, cadres of surgical/anaesthesia providers and/or patients' outcomes.

Results: The 52 articles included in analysis provided information about 16 countries. District hospitals were a group of diverse institutions ranging from 21 to 371 beds. The 3 most frequently reported procedures were caesarean section, laparotomy and hernia repair but a wide range of orthopaedics, plastic surgery and neurosurgery procedures were also mentioned. The number of operations performed per year per district hospital ranged from 239 to 5233. The most mentioned anaesthesia providers were non-physician clinicians trained in anaesthesia. They deliver mainly general and spinal anaesthesia. Depending on countries, articles referred to different surgical care providers: specialist surgeons, medical officers and non-physician clinicians. 15 articles reported peri-operative complications among which surgical site infection was the most frequent. 15 articles reported perioperative deaths of which the leading causes were sepsis, haemorrhage and anaesthesia complications.

Conclusion: District hospitals play a significant role in sub-Saharan Africa, providing both emergency and elective surgery. Most procedures are done under general or spinal anaesthesia, often administered by non-physician clinicians. Depending on countries, surgical care may be provided by medical officers, specialist surgeons and/or non-physician clinicians. Research on safety, quality and volume of surgical and anaesthesia care in this setting is scarce and more attention to these questions is required.

Article Summary:

Strengths and limitations:

- This is the first review (of which we are aware) to systematically assess the literature for the scope, volume and quality of surgery in district hospitals in sub-Saharan Africa.
- Article selection and data extraction were conducted independently by two authors.
- Our search was conducted in English, French and Portuguese but work in other languages may have been omitted.

For peer review only - http://bmjopen.bmj.com/site/about/guidelines.xhtml

- This study is prone to publication bias as it includes only data from published literature, and findings that were never published will have been omitted.
- There was a lot of heterogeneity in how articles reported surgical volume and quality.

Introduction:

The burden of surgical disease in sub-Saharan Africa is estimated at 38 disability adjusted life years (DALYs) per 1,000 population. District hospitals bear a large burden of this surgical care delivery.(1) According to The World Health Organization (WHO), district hospitals are defined as the first level of healthcare facilities that provide in-patient surgery and anaesthesia.(2) These hospitals which mainly serve rural populations, face challenges in terms of workforce, infrastructure and other essential resources. In 2010, it was estimated that there was only 1 surgeon for 25 million people in rural sub-Saharan Africa (3) and although training programs in the region are increasing the number of surgeons, the main burden in several countries remains on non-physician clinicians to manage surgical cases.(4)

District hospitals offer a unique opportunity to improve access to surgical care in sub-Saharan Africa as they are the first point of access to surgical care for patients and relieve referral hospitals from undue burden, allowing them to focus on complicated cases. Delivering timely surgical care in district hospitals has been shown to be highly cost effective(5) which should provide encouragement for governments and global health programs to invest in them. The Lancet Commission on Global Surgery,(6) which defines scalable solutions for the provision of quality surgical and anaesthesia care for all, reinforces this emphasis on district hospitals and echoes other authors' calls to consider district hospitals as a central component in strengthening surgical care in sub-Saharan Africa.(7)(8)

We considered a systematic review of district hospital surgery, however there were a limited number of existing studies in this field and a wide range of methodology used. Therefore, we chose to change our review methodology and undertake a scoping review of surgical care delivery in district hospitals in sub-Saharan Africa. Our aim is to provide a general overview of the reported current surgical capacity and delivery in order to advance current knowledge and suggest targets for further development and research within the region of sub-Saharan Africa.

Methods:

This scoping review follows the five step methodology designed by Arksey and O'Malley(9) that offers a rigorous framework for reporting findings. We have also considered the recommendations of Levac, Colquhoun and O'Brien(10) that supplement this framework.

1. Identifying the research question

To guide our search of the literature, we defined the following questions:

Primary outcome:
In district hospitals in sub-Saharan Africa, what types of surgical procedures are provided?

Secondary outcomes:

For peer review only - http://bmjopen.bmj.com/site/about/guidelines.xhtml

In district hospitals in sub-Saharan Africa:

- ❖ How many surgical procedures are performed per year?
- ❖ Are there any measures of quality, safety or outcomes of surgery?
- ❖ Who provides anaesthesia and/or surgery?
- ❖ What type of anaesthesia is delivered?

2. Identifying relevant studies

We searched PubMed and Ovid EMBASE on 18/12/19 to retrieve literature about surgery in district hospitals in sub-Saharan Africa. The search strategy combined the exploded thesaurus terms “surgery”, “surgical procedures, operative” and “specialties, surgical” with the exploded thesaurus terms “hospitals, district” and “Africa south of the Sahara”. We searched references in English, French and Portuguese. We limited the search to references published after January 1st, 2000 in order to provide a recent overview and therefore studies published before 2000 were not relevant for this purpose. We also searched Google Scholar and the Cochrane Library with the same search terms, but they did not contribute any relevant new references.

3. Study selection

The most important inclusion criterion was the setting: a district-level hospital in sub-Saharan Africa. We included articles that provided information about either type of surgical procedures performed, number of operations per year, types of anaesthesia delivered, cadres of surgical/anaesthesia providers and/or patient outcomes. We excluded articles if the setting was unclear or was not a district hospital, and if they were strictly about costs or burden of disease. We excluded articles if the full text could not be retrieved. We included all surgical procedures except those typically done in outpatient departments, such as dental and ophthalmic procedures.

Z.B. and G.D. independently screened the abstracts using the web application Rayyan,(11) resolving any conflicts by dialogue between Z.B., G.D. and C.L. The process of selection followed the Preferred Reporting of Items for Systematic Reviews and Meta-Analyses (PRISMA) Statement.

4. Charting the data

Z.B. developed the data charting form after consultation with all other authors. The form was piloted and then updated iteratively during the extraction process (see supplementary file). The form contained 20 fields of data entry covering information about articles (e.g. name of first author, year of publication, research method) and information about district hospitals, surgical and anaesthesia providers, surgical procedures as well as other relevant information. Z.B. and S.S. extracted the data independently.

5. Collating, summarizing and reporting the results

S.S. produced descriptive numerical summaries of surgical volume, types of surgical procedures and types of anaesthesia using Excel. Z.B. performed a thematic analysis in

For peer review only - http://bmjopen.bmj.com/site/about/guidelines.xhtml

NVivo(12) with surgical providers, quality of care and surgical outcomes as themes. We reported results to answer the primary and secondary outcomes of the review. All authors discussed and reported implications for research and policy.

Results:

In this section, we first present characteristics of the included articles and an analysis of the district hospitals. Thereafter, we present our findings on the scope and volume of surgery, types of anaesthesia, surgical and anaesthesia providers, and finally safety and quality of care.

1. Study Selection

We identified 302 articles and after removing duplicates, 267 articles remained. We screened the titles and abstracts, and, using our criteria noted above, excluded 167 articles. Then, we assessed full texts of the remaining 100 articles and excluded 48. Therefore, we included 52 articles for analysis. A PRISMA diagram is presented in Figure 1.

Figure 1: PRISMA diagram illustrating the selection process

2. Study Characteristics

Thirty three per cent of articles (17 articles) were cross-sectional surveys, 17% were prospective studies (nine articles) and 11.5% (six articles) were based on retrospective review of hospital records (Table 1). 93 % of articles (48 articles) described the surgical care provided by staff based at the hospital, and the rest (four articles) reported external support from Non-Governmental Organisations (NGO) or visiting surgical teams (13–17)

Table 1: Summary of studies selected

First Author	Year of Publication	Study setting	Study Design	Objective
Abdullah(18)	2011	Ghana	Mixed methods	Assess surgical and obstetrical capacity
Cheelo(19)	2018	Zambia	Cross-sectional survey	Assess surgical capacity
Choo(20)	2013	Ghana	Cross-sectional survey	Appraise quality improvement activities
Choo(21)	2011	Ghana	Qualitative methods	Explore the training of medical officers providing surgery

For peer review only

Compaoré(22)	2014	Burkina Faso	Cross-sectional survey	Assess the readiness to provide caesarean sections
Damien(23)	2011	Ghana	Review of hospital records	Evaluate surgical volume
De Brouwere(24)	2009	Senegal	Mixed methods	Evaluate the task shifting policy
Dossche(14)	2014	Burkina Faso	Case series	Analyse technical problems and post-operative complications
Dresser(25)	2017	Uganda	Review of hospital records	Evaluate the management of surgical patients by non-physician emergency clinicians
Fehr(26)	2006	Tanzania	Prospective cohort study	Identify risk factors for surgical site infection
Fehr(27)	2006	Tanzania	Prospective cohort study	Evaluate peri-operative antimicrobial prophylaxis
Fenton(28)	2003	Malawi	Prospective cohort study	Identify risk factors associated with maternal death after caesarean section
Gajewski(29)	2017	Malawi	Baseline and follow up patients' surveys	Compare surgical outcomes between central and district hospitals
Gajewski(30)	2019	Malawi, Tanzania, Zambia	Mixed-methods	Investigate anaesthesia capacity

For peer review only - http://bmjopen.bmj.com/site/about/guidelines.xhtml

Gajewski(31)	2018	Malawi	Qualitative methods	Explore providers' perspectives on obstacles to surgery
Gajewski(32)	2019	Malawi	Randomised Control Trial	Evaluate surgical training programme for clinical officers
Galukande(33)	2010	Uganda, Tanzania, Mozambique	Review of hospital records	Assess the scope of surgery provided
Grimes(34)	2012	Sub-Saharan Africa	Systematic Review	Identify the met and unmet needs of surgical disease
Gyedu(35)	2018	Ghana	Cross-sectional survey	Evaluate the operation rate in Ghana
Hall(36)	2015	Ghana	Review of hospital records	Evaluate the impact of a newly placed obstetrician on health outcomes
Harfouche(37)	2015	Malawi	Prospective cohort study	Assess the quality of emergency caesarean sections
Henry(38)	2015	Sub-Saharan Africa	Consensus meeting+ interviews	Identify a package of essential surgical operations
Henry(39)	2015	Malawi	Cross-sectional survey	Assess surgical capacity
Kenfack(40)	2012	Cameroon	Case series	Describe the management of ectopic pregnancy
Koigi-Kamau(41)	2005	Kenya	Prospective cohort study	Describe the incidence of post-caesarean

For peer review only

				wound infection
Lavy(42)	2007	Malawi	Review of hospital records	Estimate surgical volume
LeBrun(43)	2014	Ethiopia, Liberia, Rwanda, Uganda	Cross-sectional survey	Assess surgical capacity
Lofgren(44)	2015	Uganda	Cross-sectional survey	Investigate indications, interventions and peri-operative mortality
Lonnée (40)	2018	Zimbabwe	Cross-sectional survey	Describe anaesthesia care for caesarean sections
Luo(45)	2018	Ghana	Qualitative methods	Explore the impact of a newly placed obstetrician in a district hospital
McCord(46)	2012	Ghana	Commentary	Describe surgical care in district hospitals in Ghana
McCord(47)	2009	Tanzania	Review of hospital records	Evaluate the quality of emergency obstetric surgery done by assistant medical officers
Mehtsun(48)	2012	Ghana	Observational study	Assess surgical workload of medical officers
Mpirimbanyi(17)	2017	Rwanda	Cross-sectional survey	Describe the spectrum, management and patient outcomes of

For peer review only

				emergency surgery
Muhirwa(15)	2016	Rwanda	Cohort study	Describe the presentation, management and outcomes of surgical patients
Ngaroua(49)	2017	Cameroon	Case series	Describe the management of urethral stenosis
Nkurunziza(16)	2019	Rwanda	Cross-sectional survey	Estimate the prevalence of surgical site infection after caesarean section
Nordberg(50)	2002	Kenya	Cross-sectional survey	Estimate the surgical volume
Notrica(51)	2011	Rwanda	Cross-sectional survey	Assess surgical and anaesthesia infrastructure
Ottaway(52)	2018	Namibia	Prospective Observational Study	Determine the prevalence of anaesthetic adverse events
Ouédraogo(53)	2015	Burkina Faso	Prospective Observational Study	Describe task shifting in obstetric care
Ouédraogo(54)	2015	Burkina Faso	Case series	Describe prognosis of patients undergoing caesarean sections
Petroze(55)	2012	Rwanda	Cross-sectional survey	Evaluate the Ratio of caesarean sections to total procedures as a marker of trauma capacity

For peer review only

Petroze(56)	2012	Rwanda	Mixed methods	Assess emergency and essential surgical capacity
Ramos(13)	2013	Ethiopia	Case series	Describe thyroid surgery cases
Rutgers(57)	2008	Zimbabwe	Cross-sectional survey	Describe the mortality associated with caesarean sections
Sani(58)	2009	Niger	Cross-sectional survey	Assess surgical care provided by general practitioners
Sherman(59)	2011	Liberia	Cross-sectional survey	Assess emergency and essential surgical care
Smiley(60)	2019	Ghana	Surveys + process mapping	Evaluate patients and staff perception of quality of care + Analyse peri-operative process
Stewart(61)	2015	Ghana	Cross-sectional survey	Assess trauma capacity
Van Amelsfoort(62)	2010	Malawi	Mixed methods	Describe the training and experiences of clinical officers
Van den Akker(63)	2009	Malawi	Mixed methods	Evaluate the impact of audits in reducing the incidence of uterine ruptures

For peer review only - http://bmjopen.bmj.com/site/about/guidelines.xhtml

Of the 52 articles included for analysis, covering 16 countries, three articles presented data from multiple countries(30,33,43) and two gave a regional perspective.(34,38) Ghana, Malawi and Rwanda were the three countries most represented in the articles selected, partly because of research projects in collaboration with researchers from High Income Countries (HIC) (Table 2)

Table 2: Distribution of articles per country

Country	Number of articles
Burkina Faso	3
Cameroon	2
Ethiopia	2
Ghana	11
Kenya	2
Liberia	2
Malawi	10
Mozambique	1
Namibia	1
Niger	1
Rwanda	7
Senegal	1
Sub Saharan Africa (regional perspective)	2
Tanzania	5
Uganda	4
Zambia	2
Zimbabwe	2

3. Characteristics of district hospitals:

District hospitals described in this review included a variety of institutions (Table 3). In our review, the two smallest district hospitals had 21 beds, reported in Liberia and Ghana by Sherman et al(59) and Choo et al(21) respectively. The biggest district hospital had 371 beds, reported by Fehr et al in Tanzania.(26) District hospitals served rural or urban populations, with some serving both groups (for example if they were situated on the edge of towns). Although most were owned by the government, mission linked district hospitals also featured in several papers. On some occasions, hospitals received support from visiting surgeons or NGOs, e.g. in Rwanda, government owned district hospitals received support from Partners in Health.(15)

Table 3: Distribution of articles per size of district hospitals

Size of district hospitals (in number of beds)	Number of articles
<20	0
20-50	2
51-100	8
101-300	9
>300	2
Not specified	33

4. Scope of surgical operations

For peer review only - http://bmjopen.bmj.com/site/about/guidelines.xhtml

For peer review only

The majority of articles mentioned obstetrics/gynaecology and general surgery operations (Table 4). Indeed, the three most frequently reported procedures were caesarean section, laparotomy and hernia repair. However, a wide range of other surgical procedures were mentioned across the literature (Supplementary File). These were mainly in orthopaedics, plastic surgery or neurosurgery and may have involved visiting specialists (Table 4). District hospitals reported in the literature handled both emergency procedures (such as caesarean sections, appendicectomies and treatment of burns); and elective procedures (such as hernia repairs) and some even more complex procedures such as direct vision internal urethrotomy(49) and hip replacement.(14) Despite the high prevalence of traumatic brain injury in sub Saharan Africa(64) and the fact that cranial burr holes are included in the WHO book "Surgical Care at the District Hospital",(65) only three articles mentioned burr holes.

Table 4: Distribution of articles per surgical operations

Surgical Procedures	Number of articles
General Surgery	
Hernia Repair	26
Laparotomy	21
Appendicectomy	10
Abscess	7
Hydrocele repair	7
Thyroid surgery	5
Colectomy	4
Chest tube insertion	4
Prostatectomy	4
Cricothyroidotomy	3
Colostomy	2
Lipoma	2
Tracheostomy	2
Fibroma	1
Urethrotomy	1
Vascular repair	1
Bladder stone surgery	1
Obstetrics and Gynaecology	
Caesarean section	30
Salpingectomy (ectopic pregnancy)	10
Hysterectomy	9
Evacuation of Retained Products of Conception	7
Tubal Ligation	5
Fistula Repair	3
Cervical tear repair	2
Breast surgery	1

For peer review only - http://bmjopen.bmj.com/site/about/guidelines.xhtml

Orthopaedics	
Amputation	10
Open Fracture repair	8
Closed Fracture repair	5
Arthrotomy	3
Osteotomy	2
Total Hip replacement	1
Bipolar Hemiarthroplasty	1
Neurosurgery	
Major neurosurgery	1
Burr holes	3
Plastics	
Skin grafting	7
Debridement	5
Burn	4
Cleft lip/palate surgery	3
Contracture release	2
Escharotomy	1

5. Surgical volume

Only 13 articles provided enough data to be able to estimate the number of operations per year per district hospital performed. In some cases, there was not enough information to discriminate between major and minor operations, therefore the choice was made to present all operations combined. Using a simple formula; the number of operations, period of years and number of district hospitals, an average of the number of operations per district hospital per year was calculated (Table 5). Galukande et al(33) and LeBrun et al(43) contributed several reports on different district hospitals while Van Amelsfoort et al(62) contributed two reports on the same district hospital during two different time periods.

The surgical volume of district hospitals varied from circa 200 to circa 5000 operations per year, with the majority of hospitals performing between 1001 and 5000 operations per year (Tables 5 and 6). Clear limitations to the use of these data are the sample size and selection bias. Of the 13 studies, only six analysed more than five district hospitals in a given area. These data, therefore, cannot solely be used as a general indicator for the productivity of district hospitals in the countries represented.

Table 5: Surgical volume (all reported cases) in number of operations per year per district hospital

First author	Study setting	Total number of operations	Years	Number of district hospitals	Estimated number of operations per district hospital per

For peer review only - http://bmjopen.bmj.com/site/about/guidelines.xhtml

					year *(to the nearest unit)*
Abdullah~ (18)	Ghana	-	1	10	774
Damien (23)	Ghana	1,391	5	1	278
Galukande	Tanzania	980	1	1	980
Galukande	Tanzania	2,045	1	1	2,045
Galukande	Mozambique	601	1	1	601
Galukande	Mozambique	256	1	1	256
Galukande	Uganda	1,484	1	1	1,484
Galukande	Uganda	248	1	1	248
Galukande	Uganda	239	12	1	239
Galukande	Uganda	1,835	1	1	1,835
Gyedu	Ghana	143,750	1	48	2,995
Lofgren $	Uganda	-	-	2	5,018
Lavy (42)	Malawi	25,053	1	21	1,193
LeBrun*	Uganda	-	1	1	1,296
LeBrun*	Rwanda	-	1	1	2,052
LeBrun*	Liberia	-	1	1	696
LeBrun*	Ethiopia	-	1	1	538
Nordberg~	Kenya	-	1	5	5,233
Notrica*	Rwanda	-	-	-	2,052
Ottaway	Namibia	737	0.58	4	316
Petroze	Rwanda	71,432	1	41	1,742
Sani	Niger	544	1	3	181
Van Amelsfoort	Malawi	19,644	1	17	1,156
Van Amelsfoort	Malawi	18,524	1	17	1,090

* Reported in the article as average number per year per district hospital
~ The article reported numbers for both major and minor operations and we calculated the total number of procedures.
$ This article reported that the number of major operations was estimated over a 4 month period while the number of minor operations was estimated over a 3 month period.

Table 6: Number of publications reporting the number of operations per year per district hospital

Number of operations on average per district hospital over 1 year	Number of publications
<100	0
100-500	6
501-1000	5
1001-5000	11
>5000	2

For peer review only - http://bmjopen.bmj.com/site/about/guidelines.xhtml

6. Types of anaesthesia

Out of 52 articles, 16 contained information about the type of anaesthesia used in the district hospitals studied. General anaesthesia was mentioned by 15 articles(13,15,17,18,22,23,29–37) and spinal anaesthesia by 10 articles(14,17,21,28,44,52–54,59,66). Local anaesthesia and regional blocks were mentioned by 4 articles(15,17,44,59).

7. Surgical and anaesthesia providers

In each country, different cadres of healthcare professionals are responsible for providing surgical care in district hospitals (Table 7). In Ethiopia, Ramos et al reported the presence of a specialist surgeon.(13) In Ghana(18,21,35,36,45,46,48,60), Kenya(50), Rwanda(15–17,51,55,56) and Zimbabwe(57) medical officers worked alongside specialist surgeons or obstetrician/gynaecologists to provide surgical care. In Namibia(52) and Niger(58), the presence of medical officers as sole surgical providers was reported. Non-physician clinicians provided surgical care in Burkina Faso,(14,22,53) Liberia,(59) Malawi,(28,29,31,32,39) Senegal,(24) Tanzania,(30,47) Uganda(25,44) and Zambia.(19)

Similarly, different cadres performed anaesthesia in different countries.
Articles reported the presence of a physician anaesthetist in district hospitals in Burkina Faso(14) and Ghana.(60) Medical officers providing anaesthesia on their own were reported in Namibia.(52) Otherwise, the most mentioned anaesthesia providers are non-physician clinicians trained in anaesthesia (Table 8). Their presence is reported in Burkina Faso,(22) Ethiopia,(13) Ghana,(21) Liberia,(59) Malawi,(28)(20) Rwanda,(51)(56) Senegal,(24) Tanzania,(30) Uganda,(44) Zambia(19) and Zimbabwe.(57)(66) There are also untrained non-physician clinicians providing anaesthesia in Liberia,(59) Malawi,(28)(20) Rwanda,(51)(56) Zimbabwe(57)(66) alongside trained personnel.

Table 7: Number of publications reporting surgical providers

Surgical Providers	Number of Publications
Specialist Surgeon/Obstetrician	5
Specialist Surgeon/Obstetrician & Medical Officers	9
Medical Officers	8
Medical Officers & Non-Physician Clinicians	4
Specialist Surgeon/Obstetrician & Non-Physician Clinicians	1
Non-Physician Clinicians	5
Specialists & Medical Officers & Non-Physician Clinicians	8

Table 8: Number of publications reporting anaesthesia providers

Anaesthesia Providers	Number of Publications
Physician Anaesthetists	2
Medical Officers	1

For peer review only - http://bmjopen.bmj.com/site/about/guidelines.xhtml

Medical Officer & Non-Physician Clinicians	1
Non-Physician Clinicians (trained)	10
Non-Physician Clinicians (trained & untrained)	6

8. Quality of care and surgical outcomes

8.1. Quality of care

Of all articles in our review, only nine articles evaluated the quality of care as part of their study. Choo et al surveyed 10 district hospitals in Ghana about quality improvement activities.(20) None of the hospitals surveyed reported an organised quality improvement programme (although this does not mean it was absent), and the only quality improvement activity related to surgery was found in one hospital and related to sterilisation of instruments.(20) Other authors echoed these findings by reporting a lack of clinical guidelines for surgery, anaesthesia and labour management(22,41,59) and a lack of staff trained in using them when they were available.(22)

To evaluate the quality of caesarean sections, Harfouche et al measured the "decision to incision time" in a district hospital in Malawi and found that it reached "1.69 hours per caesarean section, far exceeding the recommended 30 minutes".(37) They explained their findings by a "lack of operating theatre space and high patient volume" but also by an inefficient system to transfer patients to the operating theatre.(37)

Another major challenge to quality care in district hospitals in sub-Saharan Africa is the shortage of trained surgical and anaesthesia staff. Stewart et al(61) explained that shortage in staff resulted in unavailability of service during nights and weekends. Choo et al found that medical officers in Ghana had limited exposure to surgery during their training.(21) Harfouche et al reported that while clinical officers performed most caesarean sections at district level in Malawi, they were not sufficiently trained in performing emergency hysterectomies.(37) Positive outcomes were observed in cases where trained staff were present: Hall et al reported an "association between the presence of OBGYNs in district hospitals in Ghana and an increase in evidence-based maternal care practices, as well as moderate improvement in maternal mortality and stillbirth rates".(36)

Some authors suggested interventions to improve the quality of care. Van den Akker et al suggested audit as a "simple intervention requiring little technology" that is able to improve quality of care.(63) They reported a decrease in 68% of uterine ruptures in a district hospital in Malawi after implementing recommendations inferred from an audit. Recommendations included training of staff, better documentation and involvement of hospital management.(63) Other authors insisted on the need for monitoring safety and quality of care in district hospitals as a first step toward improving quality.(20)(44)

However, Smiley et al reported care of exceptional quality in a "63 bed district hospital" named the "Volta River Authority Hospital" in Ghana(60). In their study they surveyed staff and patients about their perception of quality of care and conducted process mapping of surgical and obstetric care. They found that "over 80% of employees across a variety of perioperative roles held a positive view of teamwork and safety climate within the

For peer review only - http://bmjopen.bmj.com/site/about/guidelines.xhtml

institution" and "surgical patients who were surveyed gave similarly positive indications of overall satisfaction".(60) The perception of staff and patients was complemented by the observations of researchers during the process mapping. The WHO safety checklist was used in all operations witnessed and the authors reported the existence of a hospital wide morbidity and mortality conference as well as a safety training for new staff.(60)

8.2. Surgical outcomes

The most commonly used indicators of outcome were peri-operative complications and peri-operative mortality. 15 articles reported information about peri-operative complications,(14–17,26–29,32,37,40,41,52,54,63) the most reported being surgical site infection. 15 articles included information about peri-operative mortality;(10-12,15,16,18,20,22,23,26,32,36,41,49,50) the most reported causes of death were sepsis, haemorrhage and anaesthesia complications.

In surgical obstetric care, the most reported complications were ruptured uterus, post-partum haemorrhage and wound dehiscence.(28,37,63) The most reported cause of maternal mortality was ruptured uterus.(15,18,20,23,26,30,32,49)

Discussion:

Although it varies by country, our review found that district hospitals provide mainly emergency obstetric and general surgery and, in some cases, elective surgery covering a wide range of surgical specialties. The literature reports that, most often, surgical procedures are done under general or spinal anaesthesia.

Many of the procedures performed are by trained non-physician clinicians and medical officers. There is a paucity of data regarding quality of surgical care, surgical volume and peri-operative morbidity and mortality.

Our study is a snapshot of two decades of published surgical activity in district hospitals. We had aimed to compare it with other similar studies, but we found none that were appropriate, either in terms of similar methodology, scope or research question. We suggest that there is a challenge and an imperative for district hospitals to collect and publish information on key indicators so that standards can be measured, and quality improvement encouraged. Our preference is to start with the agreed indicators recommended by the Lancet Commission on Global Surgery.(6)

Based on the findings of this study, our recommendation is for Ministries of Health in sub-Saharan Africa to consider policies and resources that prioritize quality improvement practices for district-level surgical care in the region, including standardization and quality improvement in surgical information systems.

We have reflected on the strengths and limitations of this study. When we designed the study protocol, we followed the WHO terminology regarding 'district hospitals'.(65) If we had used the term 'rural' in our search strategy, it would have added confusion as it includes a variety of structures, many of which are health centres or dispensaries without in-patient facilities. However, we could have missed relevant literature that did not specifically use the

For peer review only - http://bmjopen.bmj.com/site/about/guidelines.xhtml

terminology 'district hospital' but instead used other terms such as 'rural hospital'. Our search strategy used the term 'Africa south of the Sahara' and this may have omitted studies that mentioned a country name but not the term 'Africa or 'south of the Sahara'. Our search included English, French and Portuguese texts but work in other languages may have been omitted. We sought to minimise subjectivity, therefore two authors independently conducted the article selection and data extraction. Our study is prone to some publication bias as it is based only on information present in published literature, and findings that were never published will have been omitted. It is unlikely that institutions that are not actively auditing activity would present it for publication, and even those district hospitals that do collect information of relevance to this study are unlikely to submit it for publication.(67) Indeed, these institutions face several barriers to publish, such as the cost of submissions to journals and overwhelming clinical demands reduce the time available for academic work. Furthermore, there is usually a cost of running trials/data collection and, if the research is published, there is often a pay wall burden resulting in a limited distribution of results for the readership in LMICs. Finally, our results are limited by the heterogeneity in how articles reported indicators such as personnel training level, surgical volume in terms of minor and major operations, complications, outcomes, and peri-operative mortality. Some articles used surgical indicators but these were usually non-standard, and the core surgical indicators (geographical access to surgical care within 2 hours, surgical providers density, total operative volume, in-hospital postoperative mortality, and impoverishing and catastrophic cost burden) recommended by the Lancet Commission on Global Surgery(6) were not routinely used. However, this is the first review to systematically assess the literature for the scope, volume and quality of surgery in district hospitals in sub-Saharan Africa, to our knowledge.

Conclusion:

District hospitals play a significant role in surgical care in sub-Saharan African countries. This scoping review has provided a broad but incomplete data set on the current provision of surgical care in district hospitals in sub-Saharan Africa as reported in published studies. Based on the findings of this study, we recommend to researchers to conduct more primary surgical systems research in district hospitals to report on surgical activity, using core indicators suggested by the Lancet Commission on Global Surgery that will improve comparability.

Authors Contribution:

Zineb Bentounsi, MD, MSc: investigation, data curation, methodology, formal analysis, writing-original draft, writing-review and editing

Sharaf Sheik Ali, MRCS (ENT), MBBS, BSc: investigation, formal analysis, writing-original draft, writing- review and editing

Grace Drury, MA: project administration, investigation, writing-review and editing

Chris Lavy, MBBS, MD: conceptualization, supervision, writing-review and editing

For peer review only - http://bmjopen.bmj.com/site/about/guidelines.xhtml

Data Sharing Statement: Data available upon request

Patient and public involvement: Patients and the public were not involved in the design or conduct of the study.

Conflict of Interest: Authors have no conflict of interest.

Funding Statement: This work was supported by SURG Africa through the European Union Horizon 2020 programme Grant Agreement no. 733391. This work was also supported by the NIHR Biomedical Research Centre, Oxford, UK.

References:

1. Luboga S, Macfarlane SB, von Schreeb J, Kruk ME, Cherian MN, Bergström S, et al. Increasing access to surgical services in sub-saharan Africa: priorities for national and international agencies recommended by the Bellagio Essential Surgery Group. PLoS Med [Internet]. 2009/12/22. 2009 Dec;6(12):e1000200–e1000200. Available from: https://pubmed.ncbi.nlm.nih.gov/20027218
2. Mulligan J-A, Fox-Rushby JA, Adam T, Johns B, Mills A. Unit Costs of Health Care Inputs in Low and Middle Income Regions. 2003.
3. Pollock JD, Love TP, Steffes BC, Thompson DC, Mellinger J, Haisch C. Is it possible to train surgeons for rural Africa? A report of a successful international program. World J Surg. 2011 Mar 30;35(3):493–9.
4. Falk R, Taylor R, Kornelsen J, Virk R. Surgical Task-Sharing to Non-specialist Physicians in Low-Resource Settings Globally: A Systematic Review of the Literature [Internet]. Vol. 44, World Journal of Surgery. Springer; 2020 [cited 2021 Feb 2]. p. 1368–86. Available from: https://pubmed.ncbi.nlm.nih.gov/31915975/
5. Roberts G, Roberts C, Jamieson A, Grimes C, Conn G, Bleichrodt R. Surgery and Obstetric Care are Highly Cost-Effective Interventions in a Sub-Saharan African District Hospital: A Three-Month Single-Institution Study of Surgical Costs and Outcomes. World J Surg. 2016;40:14–20.
6. Meara, J. G., Leather, A. J., Hagander, L., Alkire, B. C., Alonso, N., Ameh EA. Global Surgery 2030: evidence and solutions for achieving health, welfare, and economic development. Lancet. 2015;386(9993):569–624.
7. Rajbhandari R, McMahon DE, Rhatigan JJ, Farmer PE. The neglected hospital - The district hospital's central role in global health care delivery [Internet]. Vol. 382, New England Journal of Medicine. Massachussetts Medical Society; 2020 [cited 2020 Jun 3]. p. 397–400. Available from: http://www.nejm.org/doi/10.1056/NEJMp1911298
8. Grimes C, Lavy C. A plea for investment in district hospitals [Internet]. Vol. 376, The Lancet. Elsevier; 2010 [cited 2020 Jun 30]. p. 2073. Available from: http://www.thelancet.com/article/S0140673610622933/fulltext
9. Arksey H, O'Malley L. Scoping studies: towards a methodological framework. Int J Soc Res Methodol. 2005;8(1):19–32.
10. Levac D, Colquhoun H, O'Brien KK. Scoping studies: Advancing the methodology. Implement Sci. 2010;5(1):1–9.
11. Ouzzani M, Hammady H, Fedorowicz Z, Elmagarmid A. Rayyan-a web and mobile app for systematic reviews. Syst Rev [Internet]. 2016;5(1):210. Available from: http://dx.doi.org/10.1186/s13643-016-0384-4
12. QSR International. NVivo Qualitative Data Analysis Software- Version 12. 2018.

For peer review only - http://bmjopen.bmj.com/site/about/guidelines.xhtml

13. Ramos JM, Abate N, Reyes F, Belate W, Mohammed F, Gorgolas M. Thyroid surgery in a district hospital in Southern Ethiopia: experience from a rural center. 2013;37(7). Available from: http://www.ncbi.nlm.nih.gov/pubmed/23564217
14. Dossche L, Noyez JF, Ouedraogo W, Kalmogho E. Establishment of a hip replacement project in a district hospital in Burkina Faso: analysis of technical problems and peri-operative complications. Bone Joint J [Internet]. 2014;96(2):177–80. Available from: http://www.ncbi.nlm.nih.gov/pubmed/24493181
15. Muhirwa E, Habiyakare C, Hedt-Gauthier BL, Odhiambo J, Maine R, Gupta N, et al. Non-Obstetric Surgical Care at Three Rural District Hospitals in Rwanda: More Human Capacity and Surgical Equipment May Increase Operative Care. World J Surg. 2016;40(9):2109–16.
16. Nkurunziza T, Kateera F, Sonderman K, Gruendl M, Nihiwacu E, Ramadhan B, et al. Prevalence and predictors of surgical-site infection after caesarean section at a rural district hospital in Rwanda. Br J Surg [Internet]. 2019;106(2):e121–8. Available from: http://www.ncbi.nlm.nih.gov/pubmed/30620071
17. Mpirimbanyi C, Nyirimodoka A, Lin Y, Hedt-Gauthier BL, Odhiambo J, Nkurunziza T, et al. Emergency general surgery in Rwandan district hospitals: a cross-sectional study of spectrum, management, and patient outcomes. 2017;17(1):121.
18. Abdullah F, Choo S, Hesse AAJ, Abantanga F, Sory E, Osen H, et al. Assessment of surgical and obstetrical care at 10 district hospitals in Ghana using on-site interviews. J Surg Res [Internet]. 2011;171(2):461–6. Available from: http://www.ncbi.nlm.nih.gov/pubmed/20691981
19. Cheelo M, Brugha R, Bijlmakers L, Kachimba J, McCauley T, Gajewski J. Surgical Capacity at District Hospitals in Zambia: From 2012 to 2016. World J Surg. 2018 Nov 1;42(11):3508–13.
20. Choo S, Papandria D, Goldstein SD, Perry H, Hesse AAJ, Abatanga F, et al. Quality improvement activities for surgical services at district hospitals in developing countries and perceived barriers to quality improvement: findings from Ghana and the scientific literature. World J Surg [Internet]. 2013;37(11):2512–9. Available from: http://www.ncbi.nlm.nih.gov/pubmed/23897444
21. Choo S, Perry H, Hesse AAJ, Abantanga F, Sory E, Osen H, et al. Surgical training and experience of medical officers in Ghana's district hospitals. 2011;86(4). Available from: http://www.ncbi.nlm.nih.gov/pubmed/21346502
22. Compaoré GD, Sombié I, Ganaba R, Hounton S, Meda N, Brouwere V De, et al. Readiness of district and regional hospitals in Burkina Faso to provide caesarean section and blood transfusion services: a cross-sectional study. BMC Pregnancy Childbirth. 2014;14:158.
23. Damien P, Nabare C, Baiden F, Kwara E, Apanga S, Etego-Amengo S. How are surgical theatres in rural Africa utilized? A review of five years of services at a district hospital in Ghana. Trop Doct [Internet]. 2011;41(2):91–5. Available from: http://www.ncbi.nlm.nih.gov/pubmed/21421886
24. De Brouwere V, Dieng T, Diadhiou M, Witter S, Denerville E. Task shifting for emergency obstetric surgery in district hospitals in Senegal. Reprod Health Matters. 2009;17(33):32–44.
25. Dresser, Caleb; Periyanayagam, Usha; Dreifuss, Brad; Wangoda, Robert; Luyimbaazi, Julius; Bisanzo MGECCIGECC. Management and Outcomes of Acute Surgical Patients at a District Hospital in Uganda with Non-physician Emergency Clinicians. World J Surg [Internet]. 2017 [cited 2020 Apr 28]; Available from: https://rayyan.qcri.org/fulltexts/320248
26. Fehr J, Hatz C, Soka I, Kibatala P, Urassa H, Smith T, et al. Risk Factors for Surgical

For peer review only - http://bmjopen.bmj.com/site/about/guidelines.xhtml

Site Infection in a Tanzanian District Hospital: A Challenge for the Traditional National Nosocomial Infections Surveillance System Index. Infect Control Hosp Epidemiol [Internet]. 2006;27(12):1401–4. Available from: http://ovidsp.ovid.com/ovidweb.cgi?T=JS&PAGE=reference&D=ovfth&NEWS=N&AN=00004848-200612000-00019

27. Fehr J, Hatz C, Soka I, Kibatala P, Urassa H, Battegay M, et al. Antimicrobial prophylaxis to prevent surgical site infections in a rural sub-Saharan hospital. Clin Microbiol Infect [Internet]. 2006;12(12):1224–7. Available from: http://ovidsp.ovid.com/ovidweb.cgi?T=JS&PAGE=reference&D=ovfth&NEWS=N&AN=00125357-200612000-00012

28. Fenton PM, Whitty CJM, Reynolds F. Caesarean section in Malawi: prospective study of early maternal and perinatal mortality. BMJ. 2003;327(7415):587.

29. Gajewski J, Conroy R, Bijlmakers L, Mwapasa G, McCauley T, Borgstein E, et al. Quality of Surgery in Malawi: Comparison of Patient-Reported Outcomes After Hernia Surgery Between District and Central Hospitals. World J Surg. 2018;42(6):1610–6.

30. Gajewski J, Pittalis C, Lavy C, Borgstein E, Bijlmakers L, Mwapasa G, et al. Anesthesia Capacity of District-Level Hospitals in Malawi, Tanzania, and Zambia: A Mixed-Methods Study. Anesth Analg [Internet]. 2019 Apr 1 [cited 2020 Apr 28];130(4):845–53. Available from: http://ovidsp.ovid.com/ovidweb.cgi?T=JS&PAGE=reference&D=ovftu&NEWS=N&AN=00000539-900000000-96017

31. Gajewski J, Bijlmakers L, Mwapasa G, Borgstein E, Pittalis C, Brugha R. “I think we are going to leave these cases”. Obstacles to surgery in rural Malawi: a qualitative study of provider perspectives. Trop Med Int Heal [Internet]. 2018 Oct 1 [cited 2020 Apr 21];23(10):1141–7. Available from: http://ovidsp.ovid.com/ovidweb.cgi?T=JS&PAGE=reference&D=ovftt&NEWS=N&AN=00060771-201810000-00013

32. Gajewski J, Borgstein E, Bijlmakers L, Mwapasa G, Aljohani Z, Pittalis C, et al. Evaluation of a surgical training programme for clinical officers in Malawi. BJS [Internet]. 2019 Jan 1 [cited 2020 Nov 18];106(2):e156–65. Available from: https://pubmed.ncbi.nlm.nih.gov/30620067/

33. Galukande M, von Schreeb J, Wladis A, Mbembati N, de Miranda H, Kruk ME, et al. Essential surgery at the district hospital: a retrospective descriptive analysis in three African countries. PLoS Med. 2010 Mar;7(3):1–10.

34. Grimes CE, Law RSL, Borgstein ES, Mkandawire NC, Lavy CBD. Systematic review of met and unmet need of surgical disease in rural sub-Saharan Africa. World J Surg [Internet]. 2012;36(1):8–23. Available from: http://www.ncbi.nlm.nih.gov/pubmed/22057752

35. Gyedu A, Stewart B, Gaskill C, Boakye G, Appiah-Denkyira E, Donkor P, et al. Improving Benchmarks for Global Surgery: Nationwide Enumeration of Operations Performed in Ghana. Ann Surg. 2018;268(2):282–8.

36. Hall E, Sevugu J, Danso K, Adomako J, Peltzman T, Anderson FJ. A retrospective analysis of the impact of an obstetrician on delivery and care outcomes at four district hospitals in Ghana. Int J Gynaecol Obstet [Internet]. 2015;131(1):100–1. Available from: http://www.ncbi.nlm.nih.gov/pubmed/26143230

37. Harfouche M, Hosseinipour M, Kaliti S, Wilkinson J. Quality indicators and outcomes of emergency caesarean deliveries at a district-level maternity hospital. Afr J Reprod Health. 2015;19(3):61–7.

38. Henry JA, Bem C, Grimes C, Borgstein E, Mkandawire N, Thomas WEGG, et al.

For peer review only

For peer review only - http://bmjopen.bmj.com/site/about/guidelines.xhtml

Essential surgery: the way forward. World J Surg. 2015;39(4):822–32.
39. Henry J, Frenkel E, Borgstein E, Mkandawire N, Goddia C. Surgical and anaesthetic capacity of hospitals in Malawi: key insights. Heal Policy Plan [Internet]. 2015;30(8):985–94. Available from: http://ovidsp.ovid.com/ovidweb.cgi?T=JS&PAGE=reference&D=ovftq&NEWS=N&AN=00003845-201510000-00005
40. Kenfack B, Noubom M, Bongoe A, Tsatedem FA, Ngono M, Tsague GN, et al. [Ectopic pregnancy in a semi-rural area in Africa: Epidemiological, clinical and therapeutic aspects about a series of 74 cases treated at the District Hospital of Sangmelima in southern Cameroon]. Pan Afr Med J. 2012;13:71.
41. Koigi-Kamau R, Kabare LW, Wanyoike-Gichuhi J. Incidence of wound infection after Caesarean delivery in a district hospital in Central Kenya. East Afr Med J. 2005;82(7):357–61.
42. Lavy C, Tindall A, Steinlechner C, Mkandawire N, Chimangeni S. Surgery in Malawi - a national survey of activity in rural and urban hospitals. Ann R Coll Surg Engl [Internet]. 2007;89(7):722–4. Available from: http://ovidsp.ovid.com/ovidweb.cgi?T=JS&PAGE=reference&D=ovfti&NEWS=N&AN=00000639-200710000-00025
43. LeBrun DG, Chackungal S, Chao TE, Knowlton LM, Linden AF, Notrica MR, et al. Prioritizing essential surgery and safe anesthesia for the Post-2015 Development Agenda: operative capacities of 78 district hospitals in 7 low- and middle-income countries. Surgery [Internet]. 2014;155(3):365–73. Available from: http://www.ncbi.nlm.nih.gov/pubmed/24439745
44. Löfgren J, Kadobera D, Forsberg BC, Mulowooza J, Wladis A, Nordin P. District-level surgery in Uganda: Indications, interventions and perioperative mortality. Surg (United States). 2015;158(1):7–16.
45. Luo EM, Opare-Ado HS, Adomako J, Danso KA, Peltzman T, Anderson FWJJ. Completing the Maternal Care Team: OB/GYN Expertise at Rural District Hospitals in Ghana, a Qualitative Study. 2018;22(7):1085–91. Available from: http://www.ncbi.nlm.nih.gov/pubmed/29520728
46. McCord C. Facilities for Surgery Exist in Ghana, but Skills Are Lacking: Comment on "Assessing the Surgical and Obstetrics-Gynecology Workload of Medical Officers". Arch Surg [Internet]. 2012;147(6):548–9. Available from: http://ovidsp.ovid.com/ovidweb.cgi?T=JS&PAGE=reference&D=ovftm&NEWS=N&AN=00000853-201206000-00013
47. McCord C, Mbaruku G, Pereira C, Nzabuhakwa C, Bergstrom S. The quality of emergency obstetrical surgery by assistant medical officers in Tanzanian district hospitals. Health Aff (Millwood) [Internet]. 28(5):w876-85. Available from: http://www.ncbi.nlm.nih.gov/pubmed/19661113
48. Mehtsun WT, Weatherspoon K, McElrath L, Chima A, Torsu VEK, Obeng ENB, et al. Assessing the surgical and obstetrics-gynecology workload of medical officers: findings from 10 district hospitals in Ghana. Arch Surg [Internet]. 2012;147(6):542–8. Available from: http://www.ncbi.nlm.nih.gov/pubmed/22786541
49. Ngaroua, Eloundou NJ, Djibrilla Y, Asmaou O, Mbo AJ. Aspects épidémiologiques, cliniques et prise en charge de sténose urétrale chez l'adulte dans un Hôpital de District de Ngaoundéré, Cameroun. Pan Afr Med J. 2017;26.
50. Nordberg E, Mwobobia I, Muniu E. Major and minor surgery output at district level in Kenya: review and issues in need of further research. Afr J Health Sci. 2002;9(1):17–25.
51. Notrica MR, Evans FM, Knowlton LM, Kelly McQueen KA. Rwandan surgical and

For peer review only - http://bmjopen.bmj.com/site/about/guidelines.xhtml

anesthesia infrastructure: A survey of district hospitals. World J Surg. 2011;35(8):1770–80.

52. Ottaway AJ, Kabongo L. Prospective Observational Study of Intraoperative Anesthetic Events in District Hospitals in Namibia. 2018;126(2):632–8. Available from: http://www.ncbi.nlm.nih.gov/pubmed/29261548
53. Ouédraogo CM, Ouattara A, Ouédraogo A, Bikienga M, Lankoandé J. Amélioration de l'offre des soins obstétricaux par la délégation des tâches en milieu Africain Exemple de l'hôpital du district sanitaire de Bogodogo à Ouagadougou, Burkina Faso. Med Sante Trop. 2015;25(3):280–4.
54. Ouédraogo CM, Ouédraogo A, Ouattara A, Lankoandé J. [Cesarean deliveries in a district hospital in Ouagadougou. Epidemiological, clinical, and prognostic study of 3381 cases]. Med Sante Trop [Internet]. 25(2):194–9. Available from: http://www.ncbi.nlm.nih.gov/pubmed/26081260
55. Petroze RT, Mehtsun W, Nzayisenga A, Ntakiyiruta G, Sawyer RG, Calland JF, et al. Ratio of cesarean sections to total procedures as a marker of district hospital trauma capacity. World J Surg. 2012;36(9):2074–9.
56. Petroze RT, Nzayisenga A, Rusanganwa V, Ntakiyiruta G, Calland JF. Comprehensive national analysis of emergency and essential surgical capacity in Rwanda. 2012;99(3). Available from: http://www.ncbi.nlm.nih.gov/pubmed/22237597
57. Rutgers RAK, Van Eygen L. Mortality related to caesarean section in rural Matebeleland North Province, Zimbabwe. Cent Afr J Med [Internet]. 54(5):24–7. Available from: http://www.ncbi.nlm.nih.gov/pubmed/21650076
58. Sani R, Nameoua B, Yahaya A, Hassane I, Adamou R, Hsia RY, et al. The impact of launching surgery at the district level in Niger. World J Surg. 2009;33(10):2063–8.
59. Sherman L, Clement P, Cherian M, Ndayimirije N, MD W-L, Noel L, et al. Implementing Liberia's Poverty Reduction Strategy: An Assessment of Emergency and Essential Surgical Care. Arch Surg [Internet]. 2011;146(1):35–9. Available from: http://ovidsp.ovid.com/ovidweb.cgi?T=JS&PAGE=reference&D=ovftl&NEWS=N&AN=00000853-201101000-00008
60. Smiley K, Ofori L, Spangler C, Acquaah-Arhin R, Deh D, Enos J, et al. Safety Culture and Perioperative Quality at the Volta River Authority Hospital in Akosombo, Ghana. 2019;43(1):16–23. Available from: http://www.ncbi.nlm.nih.gov/pubmed/30109388
61. Stewart BT, Quansah R, Gyedu A, Ankomah J, Donkor P, Mock C. Strategic Assessment of Trauma Care Capacity in Ghana. World J Surg. 2015;39(10):2428–40.
62. Van Amelsfoort JJC, Van Leeuwen PAM, Jiskoot P, Ratsma YEC. Surgery in Malawi – the training of clinical officers. Trop Doct. 2010;40(2):74–6.
63. van den Akker T, Mwagomba B, Irlam J, van Roosmalen J. Using audits to reduce the incidence of uterine rupture in a Malawian district hospital. Int J Gynaecol Obstet [Internet]. 2009;107(3):289–94. Available from: http://www.ncbi.nlm.nih.gov/pubmed/19846089
64. Hyder AA, Wunderlich CA, Puvanachandra P, Gururaj G, Kobusingye OC. The impact of traumatic brain injuries: A global perspective. Vol. 22, NeuroRehabilitation. IOS Press; 2007. p. 341–53.
65. World Health Organization. Surgical Care at the District Hospital. 2003.
66. Lonnee H, Madzimbamuto F, Erlandsen O, Vassenden A, Chikumba E, Dimba R, et al. Anesthesia for Cesarean Delivery: A Cross-Sectional Survey of Provincial, District, and Mission Hospitals in Zimbabwe. Anesth Analg [Internet]. 2018;126(6):2056–64. Available from: http://ovidsp.ovid.com/ovidweb.cgi?T=JS&PAGE=reference&D=ovftt&NEWS=N&AN=00000539-201806000-00040

For peer review only - http://bmjopen.bmj.com/site/about/guidelines.xhtml

67. Kelaher M, Ng L, Knight K, Rahadi A. Equity in global health research in the new millennium: Trends in first-authorship for randomized controlled trials among low and middle-income country researchers 1990-2013. Int J Epidemiol [Internet]. 2016 Dec 1 [cited 2021 Feb 2];45(6):2174–83. Available from: https://pubmed.ncbi.nlm.nih.gov/28199646/

For peer review only

For peer review only - http://bmjopen.bmj.com/site/about/guidelines.xhtml

PRISMA 2009 Flow Diagram

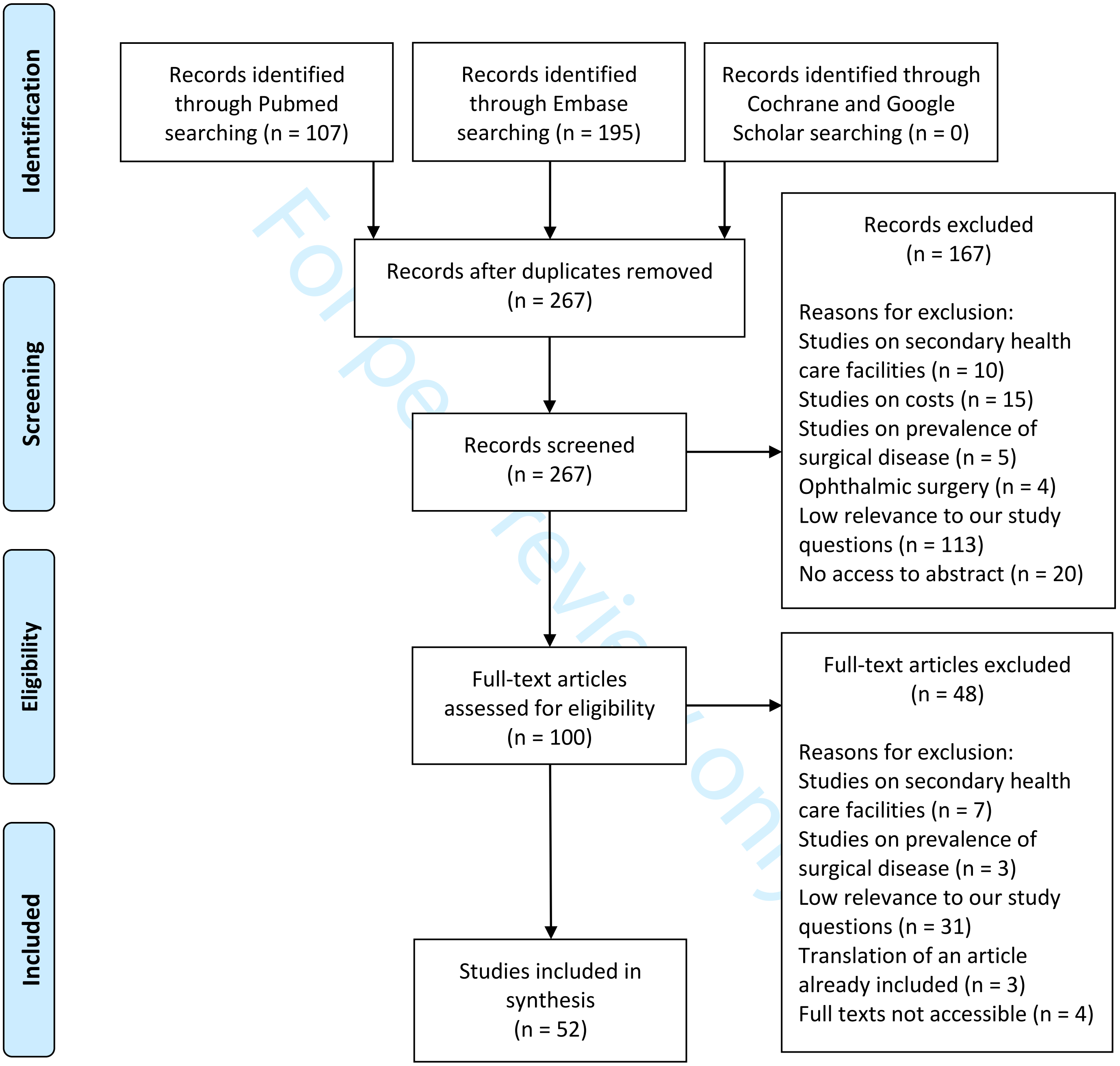

From: Moher D, Liberati A, Tetzlaff J, Altman DG, The PRISMA Group (2009). *P*referred *R*eporting *I*tems for *S*ystematic Reviews and *M*eta-*A*nalyses: The PRISMA Statement. PLoS Med 6(7): e1000097. doi:10.1371/journal.pmed1000097

For more information, visit www.prisma-statement.org.

For peer review only - http://bmjopen.bmj.com/site/about/guidelines.xhtml

Data extraction form for the scoping review

Surgical care in District Hospitals in sub Saharan Africa: a Scoping Review

* Required

1. First Author *

2. Year of publication *

3. Year of data collection (latest date to be chosen) *

4. Type of study/Research Method *

5. Country *

Check all that apply.

- [ ] Angola
- [ ] Burundi
- [ ] DRC
- [ ] Cameroon
- [ ] Central African Republic
- [ ] Chad
- [ ] Republic of the Congo
- [ ] Equatorial Guinea
- [ ] Gabon
- [ ] Kenya
- [ ] Nigeria
- [ ] Rwanda
- [ ] Sao Tome
- [ ] Tanzania
- [ ] Uganda
- [ ] Sudan
- [ ] South Sudan
- [ ] Djibouti
- [ ] Eritrea
- [ ] Ethiopia
- [ ] Somalia
- [ ] Botswana
- [ ] Comores
- [ ] Lesotho
- [ ] Madagascar
- [ ] Malawi
- [ ] Mauritius
- [ ] Mozambique
- [ ] Namibia
- [ ] Seychelles
- [ ] South Africa
- [ ] Swaziland
- [ ] ZAMBIA
- [ ] Zimbabwe
- [ ] Benin
- [ ] Mali
- [ ] Burkina Faso
- [ ] Cape Verde

For peer review only - http://bmjopen.bmj.com/site/about/guidelines.xhtml

- [ ] Ivory Cost
- [ ] Gambia
- [ ] Ghana
- [ ] Guinea
- [ ] Guinea-Bissau
- [ ] Liberia
- [ ] Mauritania
- [ ] Niger
- [ ] Senegal
- [ ] Sierra Leone
- [ ] Togo

Other: [ ] ____________________

6. Specialty *

Check all that apply.

- [ ] Obstetrics and Gynecology
- [ ] General Surgery
- [ ] Trauma and Orthopedics
- [ ] Head and Neck
- [ ] Urology
- [ ] Anesthesia
- [ ] Neurosurgery
- [ ] Paediatric surgery

Other: [ ] ____________________

7. Name of surgical procedures (please write all procedures mentioned) or write NA if none *

8. Surgical Provider *

Check all that apply.

- [ ] Specialist Surgeon (including OBGY)
- [ ] Medical Officer/GP
- [ ] Non Physician Clinician
- [ ] Nurse/Midwife
- [ ] Not specified

Other: [ ] ______________________

9. Type of Anaesthesia *

Check all that apply.

- [ ] General
- [ ] Regional Block
- [ ] Local
- [ ] Spinal
- [ ] Not specified

10. Anaesthesia Provider *

Check all that apply.

- [ ] Physician (anaesthesiologist)
- [ ] Non Physician- trained
- [ ] Non Physician- untrained
- [ ] Not specified
- [ ] Medical officer/Physician non anaesthesiologist

11. Number of operations per year/period (if available)

____

12. Catchment population (if available)

____

For peer review only - http://bmjopen.bmj.com/site/about/guidelines.xhtml

13. Equipment (if in a table, just write "table X" here)

14. Patients Outcomes (complications, survival...)

15. What do authors use as indicators for safety? (just list them)

16. What do authors use as indicators for surgical capacity?

17. Was the surgical care provided as a result of a temporary surgical mission or international partnership? *

Mark only one oval.

- Yes
- No
- It is unclear

18. Characteristics of the District Hospital(s) if given: number of bed, number of staff

19. Other important results

20. Recommendations made by authors

This content is neither created nor endorsed by Google.

Google Forms

Surgical Operations mentioned:

First Author	Country	Name of surgical procedures mentioned in text
Compaoré	Burkina Faso	caesarean section
Dossche	Burkina Faso	Total hip replacement, bipolar hemiarthroplasty
Ouedraogo C.M	Burkina Faso	Caesarean section
Ouedraogo	Burkina Faso	Caesarean section, laparotomy, cervical and perineal surgical repair
Kenfack	Cameroon	laparotomy for ectopic pregnancy
Ngaroua	Cameroon	Optical internal urethrotomy
Ramos	Ethiopia	subtotal thyroidectomy, hemi-thyroidectomy
Abdullah	Ghana	caesarean section, hernia repair, evacuation of retained products of conception from the uterus, Abdominal hysterectomy, Arthrotomy, Hand surgery, laparotomy, amputation, appendectomy, salpingectomy (e.g. ectopic pregnancy), internal fixation of fracture, other uterine procedure
Choo	Ghana	caesarean section, laparotomy, hernia repair, appendectomy, abortion
Choo	Ghana	Not specified
Damien	Ghana	caesarean sections; hernia repair and other laparotomies
Gyedu	Ghana	obstetric fistula repair, hysterectomy for uterine rupture, or intractable postpartum haemorrhage, bowel obstruction, construction of colostomy, trauma laparotomy, and skin grafting
Hall	Ghana	Caesarean section
Luo	Ghana	Caesarean section, hysterectomy
McCord C	Ghana	NA
Mehtsun	Ghana	Caesarean section, hernia, ulcer requiring debridement, cellulitis requiring debridement, abscess, trauma, burn
Smiley	Ghana	breast lumpectomy, circumcision, hernia repair, caesarean section,
Stewart	Ghana	basic general surgery, major general surgery, vascular repair, skin grafting, closed reduction, skin or skeletal traction, external fixation, internal fixation, basic neurosurgery, major neurosurgery, spine fixation
Koigi-Kamau R	Kenya	Caesarean section
Nordberg	Kenya	Caesarean section, hernia repair, exploratory laparotomy, operation on stomach, tubal ligation, prostatectomy, amputation, hydrocele, hysterectomy, myomectomy, adnexectomy, mastectomy, appendectomy, ectopic pregnancy, cleft lip/palate, club foot, thyroidectomy, open reduction of fracture, eyes/lens removal,

For peer review only

		orchidectomy, haemorrhoidectomy, splenectomy, vesico-vaginal fistula repair.
Sherman	Liberia	incision and drainage of abscesses, suturing, dilatation and curettage, male circumcision, burn care, wound debridement, appendectomy, caesarean section, hernia repair, laparotomy, cricothyroidotomy, chest tube insertion , foreign body removal, congenital hernia repair, contracture release, closed fracture repair, open fracture repair, management of osteomyelitis, amputation, biopsy, tubal ligation, dilation, curettage.
Fenton	Malawi	Caesarean section
Gajewski	Malawi	hysterectomy, ectopic pregnancy–salpingectomy, inguinal herniotomy, herniorrhaphy, prostatectomy, appendicectomy, repair of stab wounds, repair of gastrointestinal perforation, gastrointestinal resection with anastomosis or colostomy, other laparotomy, amputation of arm or leg, hydrocele excision and repair, cataract removal
gajewski	Malawi	hernia repair
Gajewski J	Malawi	not provided
Harfouche	Malawi	Caesarean section
Henry J	Malawi	resuscitation, cricothyroidotomy, tracheostomy, chest tube insertion, burn management, skin grafting, contracture release, splinting and casting, traction for closed fracture, open fracture, osteomyelitis management, amputation, appendectomy, hernia repair, bowel resection, laparotomy, biopsy, cholecystectomy,
lavy	Malawi	Dilatation and curettage, Caesarean section, Suture of wound, Manipulation of fracture, Hernia repair, Debridement of wound, Laparotomy, osteomyelitis surgery, Arthrotomy, Debridement of open fracture, Insertion of skeletal traction pin, Tenotomy, Skin graft, Osteotomy, Internal fixation of fracture, Club foot correction, Burr hole
Van Amelsfoort	Malawi	NA
van den Akker	Malawi	uterine rupture
Ottaway	Namibia	Caesarean section
Sani	Niger	Emergency: caesarean section, laparotomy for acute peritonitis, repair of strangulated hernia, laparotomy for intestinal obstruction, Elective: Hydrocele, Inguinal hernia, Bladder stone, Umbilical hernia, Lipoma, Ovarian Cyst, uterine fibroma, ectopic testicle, uterine prolapse
Mpirimbanyi C	Rwanda	The most common primary diagnoses were soft tissue infections followed by acute abdominal conditions, complicated hernias, urological emergencies and thoracic emergencies. Within soft tissue infections, abscesses followed by pyomyositis were the most common diagnoses. Bowel obstruction followed by volvulus were the most common type of acute abdominal conditions.

For peer review only

Muhirwa	Rwanda	excision of cysts, lipomas, keloids, and masses abscess, incision and drainage hernia/hydrocele repair, laparotomy, closed reduction of fracture, biopsy, amputation, Haemorrhoidectomy, mastectomy
Nkurunziza	Rwanda	Lower (uterine) segment caesarean section
Notrica	Rwanda	NA
Petroze	Rwanda	caesarean section, herniorrhaphy, amputation, closed fracture repair, chest tube insertion, laparotomy, emergency airway, wound debridement,
Petroze	Rwanda	NA
Le Brun	Rwanda, Uganda, Ethiopia, Liberia	NA
De Brouwere	Senegal	Caesarean section
Henry	SSA	Caesarean section symphysiotomy, assisted or manipulative delivery. ERPC, B-Lynch suture, repair of uterine perforation. Incision and drainage of abscess, fasciotomy, dental extraction, tympanotomy, bone drilling, arthrotomy. Debridement, haemostasis, suturing, escharotomy, skin grafting. Management of head injury, cranial burr holes, elevation of depressed skull fracture. Management of compromised airway, tracheostomy, cricothyroidotomy, removal of foreign body. Intercostal drainage, thoracostomy. Emergency laparotomy including appendectomy. Reduction of fractures and dislocations, casting and splinting, external Fixation. Amputations. Suprapubic catheterization. Hernia repair. Cataract extraction and IOL insertion. Casting and splinting, tenotomy. Cleft lip repair.
grimes	Sub Saharean Africa	Hysterectomy, Ectopic Pregnancy, Tubal Ligation, Caesarean section, Uterine evacuation, Wound-related procedures, Herniorrhaphy, Laparotomy, Appendectomy, Skin graft, Prostatectomy, Hydrocele, Strangulated hernia, Chest drain, Burr holes/craniotomy, Thoracotomy, Incision and drainage of abscess, Circumcision, Amputation
Fehr	Tanzania	Caesarean sections were the surgical interventions performed most frequently, followed by other gynaecological procedures.
Fehr	Tanzania	Caesarean Section, hysterectomy, laparotomy, hernia repair, hydrocele repair, orthopaedics
McCord	Tanzania	emergency obstetrical operations
Gajewski, Jakub	Tanzania, Malawi, ZAMBIA	Anaesthesia care
Galukande	Tanzania, Uganda, Mozambiq ue	caesarean section, wound related procedures, hernia repair, uterine evacuation, laparotomy, open fracture, tubal ligation, hysterectomy, hydrocele, circumcision, appendectomy, biopsy of lump, salpingectomy, limb amputation, skin graft, extraction of placenta, cystostomy, osteotomy/sequestrectomy, foreign body removal, orchiectomy, haemorrhoidectomy, splenectomy, mastectomy, prostatectomy, cervical tear repair, vesico-vaginal fistula repair

For peer review only

dresser	Uganda	lacerations, hernias, gastrointestinal perforation, bowel obstruction, ectopic pregnancy, hemoperitoneum, fracture, peritonitis, burns, splenic lacerations, degloving injury.
Lofgren	Uganda	Caesarean delivery, Evacuation of uterus, Herniorrhaphy, Explorative laparotomy, hysterectomy, Salpingo/oophorectomy or removal of tubal/ovarian mass, appendectomy, tubal ligation, repair of cleft palate, laparotomy for ectopic pregnancy, circumcision, repair of cuts and tears, incision and drainage of abscess, reduction of fracture, excision of granuloma of the umbilical cord, excision of mass, incision of tongue tie, reduction of dislocated joint, biopsy, removal of foreign body. The most invasive orthopaedic procedure was insertion of attraction pin in the tibial tuberosity in femur fractures
Cheelo	ZAMBIA	Caesarean section and general surgical procedures
Lonnée,	Zimbabwe	NA
RUTGERS	Zimbabwe	caesarean section

For peer review only

Preferred Reporting Items for Systematic reviews and Meta-Analyses extension for Scoping Reviews (PRISMA-ScR) Checklist

SECTION	ITEM	PRISMA-ScR CHECKLIST ITEM	REPORTED ON PAGE #
**TITLE**			
Title	1	Identify the report as a scoping review.	Title
**ABSTRACT**			
Structured summary	2	Provide a structured summary that includes (as applicable): background, objectives, eligibility criteria, sources of evidence, charting methods, results, and conclusions that relate to the review questions and objectives.	Page 2
**INTRODUCTION**			
Rationale	3	Describe the rationale for the review in the context of what is already known. Explain why the review questions/objectives lend themselves to a scoping review approach.	Page 3
Objectives	4	Provide an explicit statement of the questions and objectives being addressed with reference to their key elements (e.g., population or participants, concepts, and context) or other relevant key elements used to conceptualize the review questions and/or objectives.	Page 3
**METHODS**			
Protocol and registration	5	Indicate whether a review protocol exists; state if and where it can be accessed (e.g., a Web address); and if available, provide registration information, including the registration number.	Prospero didn't accept to register a scoping review protocol
Eligibility criteria	6	Specify characteristics of the sources of evidence used as eligibility criteria (e.g., years considered, language, and publication status), and provide a rationale.	Page 4
Information sources*	7	Describe all information sources in the search (e.g., databases with dates of coverage and contact with authors to identify additional sources), as well as the date the most recent search was executed.	Page 4
Search	8	Present the full electronic search strategy for at least 1 database, including any limits used, such that it could be repeated.	Page 4 +supplementary material
Selection of sources of evidence†	9	State the process for selecting sources of evidence (i.e., screening and eligibility) included in the scoping review.	Page 4
Data charting process‡	10	Describe the methods of charting data from the included sources of evidence (e.g., calibrated forms or forms that have been tested by the team before their use, and whether data charting was done independently or in duplicate) and any processes for obtaining and confirming data from investigators.	Page 4
Data items	11	List and define all variables for which data were sought and any assumptions and simplifications made.	NA
Critical appraisal	12	If done, provide a rationale for conducting a critical	NA

For peer review only - http://bmjopen.bmj.com/site/about/guidelines.xhtml

SECTION	ITEM	PRISMA-ScR CHECKLIST ITEM	REPORTED ON PAGE #
of individual sources of evidence§		appraisal of included sources of evidence; describe the methods used and how this information was used in any data synthesis (if appropriate).	
Synthesis of results	13	Describe the methods of handling and summarizing the data that were charted.	Page 4
**RESULTS**			
Selection of sources of evidence	14	Give numbers of sources of evidence screened, assessed for eligibility, and included in the review, with reasons for exclusions at each stage, ideally using a flow diagram.	Page 5
Characteristics of sources of evidence	15	For each source of evidence, present characteristics for which data were charted and provide the citations.	Pages 5-17
Critical appraisal within sources of evidence	16	If done, present data on critical appraisal of included sources of evidence (see item 12).	NA
Results of individual sources of evidence	17	For each included source of evidence, present the relevant data that were charted that relate to the review questions and objectives.	Pages 5-17
Synthesis of results	18	Summarize and/or present the charting results as they relate to the review questions and objectives.	Page 5-17
**DISCUSSION**			
Summary of evidence	19	Summarize the main results (including an overview of concepts, themes, and types of evidence available), link to the review questions and objectives, and consider the relevance to key groups.	Page 17
Limitations	20	Discuss the limitations of the scoping review process.	Page 17
Conclusions	21	Provide a general interpretation of the results with respect to the review questions and objectives, as well as potential implications and/or next steps.	Page 18
**FUNDING**			
Funding	22	Describe sources of funding for the included sources of evidence, as well as sources of funding for the scoping review. Describe the role of the funders of the scoping review.	Page 19

JBI = Joanna Briggs Institute; PRISMA-ScR = Preferred Reporting Items for Systematic reviews and Meta-Analyses extension for Scoping Reviews.

* Where *sources of evidence* (see second footnote) are compiled from, such as bibliographic databases, social media platforms, and Web sites.

† A more inclusive/heterogeneous term used to account for the different types of evidence or data sources (e.g., quantitative and/or qualitative research, expert opinion, and policy documents) that may be eligible in a scoping review as opposed to only studies. This is not to be confused with *information sources* (see first footnote).

‡ The frameworks by Arksey and O'Malley (6) and Levac and colleagues (7) and the JBI guidance (4, 5) refer to the process of data extraction in a scoping review as data charting.

§ The process of systematically examining research evidence to assess its validity, results, and relevance before using it to inform a decision. This term is used for items 12 and 19 instead of "risk of bias" (which is more applicable to systematic reviews of interventions) to include and acknowledge the various sources of evidence that may be used in a scoping review (e.g., quantitative and/or qualitative research, expert opinion, and policy document).

For peer review only - http://bmjopen.bmj.com/site/about/guidelines.xhtml

From: Tricco AC, Lillie E, Zarin W, O'Brien KK, Colquhoun H, Levac D, et al. PRISMA Extension for Scoping Reviews (PRISMAScR): Checklist and Explanation. Ann Intern Med. 2018;169:467–473. doi: 10.7326/M18-0850.

For peer review only
